# Supplementary material for: Cost-effectiveness of physical activity interventions in adolescents: model development and illustration using two exemplar interventions
Source: BMJ Open. 2019 Aug 18;9(8):e027566. doi: 10.1136/bmjopen-2018-027566 (PMC6701678; doi:10.1136/bmjopen-2018-027566)
Supplement: Supplementary data [file bmjopen-2018-027566supp001.pdf]

Online Supplemental File

A. Model input parameters

Table S1: Physical activity at baseline and estimates of disease incidence

Parameters	Male				Female				Distribution	Source
	Inactive	Low	Moderate	High	Inactive	Low	Moderate	High		
Baseline PA levels	12%	7%	25%	56%	23%	35%	20%	22%	Fixed	[1]
Relative risk (95% CI) estimates used to adjust disease incidence										
CHD	1.0	0.87 (0.80–0.95)	0.78 (0.74–0.82)	0.70 (0.66–0.75)	1.0	0.87 (0.80–0.95)	0.78 (0.80–0.95)	0.70 (0.80–0.95)	lognormal	[2]
Stroke	1.0	0.85 (0.80–0.91)	0.81 (0.74–0.88)	0.76 (0.68–0.85)	1.0	0.85 (0.80–0.91)	0.81 (0.74–0.88)	0.76 (0.68–0.85)	lognormal	[2]
Heart failure	1.0	0.85 (0.79–0.92)	0.78 (0.75–0.82)	0.70 (0.67–0.73)	1.0	0.85 (0.79–0.92)	0.78 (0.75–0.82)	0.70 (0.67–0.73)	lognormal	[3]
Type 2 diabetes	1.0	0.93 (0.92–0.95)	0.75 (0.69–0.80)	0.60 (0.51–0.70)	1.0	0.93 (0.92–0.95)	0.75 (0.69–0.80)	0.60 (0.51–0.70)	lognormal	[4]
Breast cancer	-	-	-	-	1.0	0.97 (0.94–0.998)	0.94 (0.90–0.98)	0.86 (0.83–0.90)	lognormal	[5]
Colorectal cancer	1.0	0.90 (0.85–0.95)	0.83 (0.77–0.97)	0.79 (0.74–0.85)	1.0	0.90 (0.85–0.95)	0.83 (0.77–0.97)	0.79 (0.74–0.85)	lognormal	[5]

Gc VS, *et al. BMJ Open* 2019; 9:e027566. doi: 10.1136/bmjopen-2018-027566

Parameters	Male				Female				Distribution	Source
	Inactive	Low	Moderate	High	Inactive	Low	Moderate	High		
CHD incidence										[6]
<35	<0.01%	<0.01%	<0.01%	<0.01%	<0.01%	<0.01%	<0.01%	<0.01%	beta	
35-44	0.01%	0.00%	<0.01%	<0.01%	<0.01%	<0.01%	<0.01%	<0.01%	beta	
45-54	0.10%	0.09%	0.08%	0.07%	0.02%	0.01%	0.01%	0.01%	beta	
55-64	0.41%	0.36%	0.32%	0.29%	0.11%	0.10%	0.09%	0.08%	beta	
65-74	1.08%	0.94%	0.84%	0.76%	0.44%	0.38%	0.34%	0.31%	beta	
75+	1.67%	1.45%	1.30%	1.17%	0.67%	0.59%	0.53%	0.47%	beta	
Stroke incidence										[7]
<35	<0.01%	<0.01%	<0.01%	<0.01%	<0.01%	<0.01%	<0.01%	<0.01%	beta	
35-44	<0.01%	<0.01%	<0.01%	<0.01%	0.01%	0.01%	0.01%	0.01%	beta	
45-54	0.01%	0.01%	<0.01%	<0.01%	0.01%	0.01%	0.01%	0.01%	beta	
55-64	0.03%	0.03%	0.03%	0.02%	0.02%	0.02%	0.02%	0.02%	beta	
65-74	0.08%	0.07%	0.07%	0.06%	0.05%	0.05%	0.04%	0.04%	beta	
75-84	0.41%	0.35%	0.33%	0.31%	0.28%	0.23%	0.22%	0.21%	beta	
Heart failure incidence										[8]
<35	<0.01%	<0.01%	<0.01%	<0.01%	<0.01%	<0.01%	<0.01%	<0.01%	beta	
35-44	0.03%	0.02%	0.02%	0.02%	0.03%	0.02%	0.02%	0.02%	beta	
45-54	0.04%	0.03%	0.03%	0.03%	0.01%	0.01%	0.01%	0.01%	beta	
55-64	0.21%	0.18%	0.16%	0.14%	0.08%	0.07%	0.07%	0.06%	beta	
65-74	0.48%	0.41%	0.37%	0.33%	0.28%	0.24%	0.22%	0.19%	beta	
75-84	1.14%	0.97%	0.89%	0.80%	0.64%	0.55%	0.50%	0.45%	beta	

Gc VS, *et al. BMJ Open* 2019; 9:e027566. doi: 10.1136/bmjopen-2018-027566

Parameters	Male				Female				Distribution	Source
	Inactive	Low	Moderate	High	Inactive	Low	Moderate	High		
Type 2 diabetes incidence										[9]
<20	0.01%	0.01%	0.01%	0.01%	0.03%	0.03%	0.03%	0.03%	beta	
20–29	0.05%	0.04%	0.04%	0.03%	0.14%	0.12%	0.11%	0.10%	beta	
30–39	0.17%	0.15%	0.13%	0.13%	0.24%	0.20%	0.18%	0.17%	beta	
40–49	0.51%	0.44%	0.38%	0.37%	0.38%	0.32%	0.28%	0.27%	beta	
50–59	0.99%	0.85%	0.74%	0.71%	0.67%	0.57%	0.50%	0.48%	beta	
60–69	1.43%	1.23%	1.07%	1.03%	1.02%	0.88%	0.76%	0.74%	beta	
70–79	1.53%	1.31%	1.15%	1.11%	1.23%	1.06%	0.92%	0.89%	beta	
80–89	1.05%	0.90%	0.79%	0.76%	0.87%	0.74%	0.65%	0.63%	beta	
Breast cancer incidence										[10]
<25					<0.01%	<0.01%	<0.01%	<0.01%	beta	
25 to 29					0.01%	0.01%	0.01%	0.01%	beta	
30 to 34					0.03%	0.03%	0.03%	0.02%	beta	
35 to 39					0.07%	0.07%	0.06%	0.05%	beta	
40 to 44					0.13%	0.13%	0.12%	0.10%	beta	
45 to 49					0.24%	0.23%	0.21%	0.19%	beta	
50 to 54					0.30%	0.29%	0.27%	0.23%	beta	
55 to 59					0.29%	0.28%	0.27%	0.23%	beta	
60 to 64					0.38%	0.36%	0.34%	0.29%	beta	
65 to 69					0.44%	0.42%	0.40%	0.34%	beta	
70 to 74					0.37%	0.35%	0.33%	0.29%	beta	
75 to 79					0.40%	0.39%	0.37%	0.32%	beta	
80 to 84					0.44%	0.42%	0.40%	0.34%	beta	

Gc VS, *et al. BMJ Open* 2019; 9:e027566. doi: 10.1136/bmjopen-2018-027566

Parameters	Male				Female				Distribution	Source
	Inactive	Low	Moderate	High	Inactive	Low	Moderate	High		
Colorectal cancer incidence										[11]
<30	<0.01%	<0.01%	<0.01%	<0.01%	<0.01%	<0.01%	<0.01%	<0.01%	beta	
30 to 34	0.01%	0.01%	0.01%	0.01%	0.01%	0.01%	0.01%	0.01%	beta	
35 to 39	0.01%	0.01%	0.01%	0.01%	0.01%	0.01%	0.01%	0.01%	beta	
40 to 44	0.01%	0.01%	0.01%	0.01%	0.01%	0.01%	0.01%	0.01%	beta	
45 to 49	0.03%	0.03%	0.02%	0.02%	0.02%	0.02%	0.02%	0.02%	beta	
50 to 54	0.06%	0.05%	0.05%	0.05%	0.04%	0.04%	0.04%	0.03%	beta	
55 to 59	0.10%	0.09%	0.08%	0.08%	0.07%	0.06%	0.06%	0.06%	beta	
60 to 64	0.19%	0.17%	0.15%	0.15%	0.11%	0.10%	0.09%	0.09%	beta	
65 to 69	0.26%	0.23%	0.21%	0.20%	0.15%	0.13%	0.12%	0.12%	beta	
70 to 74	0.37%	0.34%	0.31%	0.29%	0.22%	0.20%	0.18%	0.17%	beta	
75 to 79	0.44%	0.40%	0.37%	0.35%	0.26%	0.24%	0.22%	0.21%	beta	
80 to 84	0.54%	0.49%	0.45%	0.43%	0.34%	0.31%	0.28%	0.27%	beta	

Inactive: <30; Low: 30-149; Moderate: 150-420 and High: ≥421 MVPA min per week

Table S2: Estimates of mortality parameters

Parameter	Value				Distribution	Source
	Male Mean	Male SE	Female mean	Female SE		
CHD case fatality rate						[6]
<54 years	0.129	0.013	0.1245	0.0125	beta	
55-64 years	0.132	0.013	0.1597	0.0160	beta	
65-74 years	0.177	0.018	0.2235	0.0224	beta	
75-84 years	0.244	0.024	0.3009	0.0301	beta	
85+ years	0.315	0.032	0.3668	0.0367	beta	
Stroke case fatality rate	0.1730	0.0152	0.1730	0.0152	beta	[12]
Heart failure case fatality rate						[13]
<35 years	0.0000	0.0000	0.0000	0.0000	beta	
35-55 years	0.0989	0.0050	0.0306	0.0001	beta	
55-64 years	0.1253	0.0037	0.1094	0.0001	beta	
65-74 years	0.1666	0.0028	0.1197	0.0001	beta	
75-84 years	0.1989	0.0027	0.1262	0.0001	beta	
85+ years	0.2279	0.0041	0.1240	0.0001	beta	
Death rate after 1st nonfatal CHD						[14]
<45 years	0.0130	0.0020	0.0130	0.0050	beta	
45-54 years	0.0170	0.0010	0.0260	0.0030	beta	
55-64 years	0.0380	0.0020	0.0350	0.0020	beta	
65-74 years	0.0830	0.0020	0.0820	0.0030	beta	
75-84 years	0.1430	0.0040	0.1590	0.0040	beta	
85+ years	0.2620	0.0110	0.2660	0.0080	beta	
SMR for patients after a 1st nonfatal stroke	2.40	0.08	2.21	0.10	Lognormal	[15]
Death rate after 1st nonfatal heart failure						[13]
<55 years	0.0306	0.0001	0.0306	0.0001	beta	
55-64 years	0.1094	0.0001	0.1094	0.0001	beta	
65-74 years	0.1197	0.0001	0.1197	0.0001	beta	
75-84 years	0.1262	0.0001	0.1262	0.0001	beta	
85+ years	0.1240	0.0001	0.1240	0.0001	beta	
HR for death among patients with type 2 diabetes	1.80	0.0269	1.80	0.0269	lognormal	[16]
Breast cancer mortality	–	–	0.0290	0.0004	beta	[17]
Colorectal cancer mortality	0.1038	0.0006	0.1035	0.0006	beta	[17]
Lung cancer mortality	0.3523	0.0004	0.3112	0.0005	beta	[17]

HR = Hazard ratio; SMR = Standardised mortality ratio

Gc VS, *et al. BMJ Open* 2019; 9:e027566. doi: 10.1136/bmjopen-2018-027566

B. Model validation results

1. Cohort profile

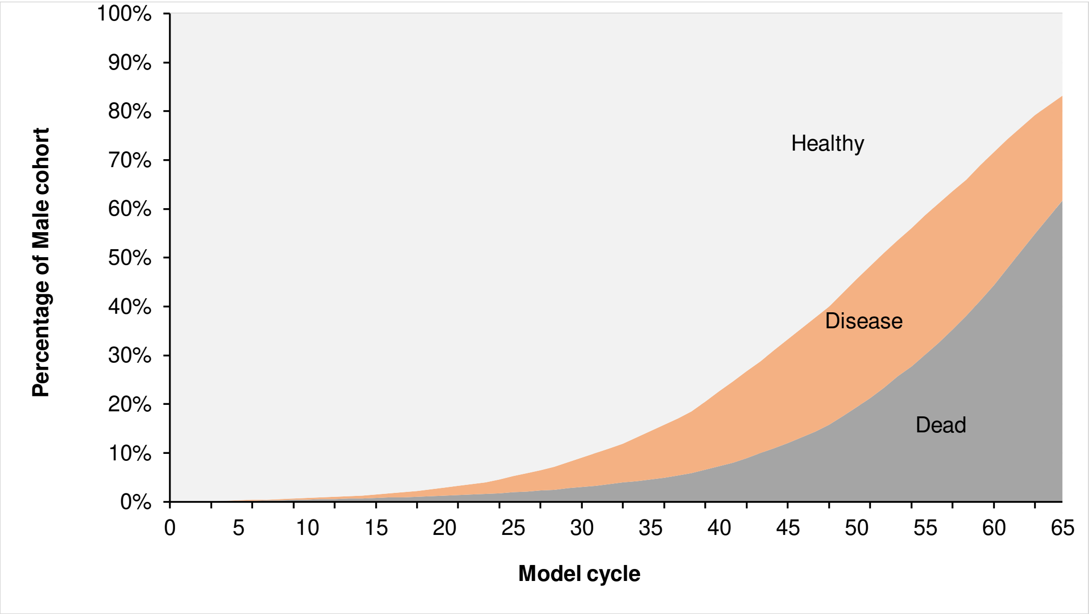

Figure S1: Cohort profile (male)

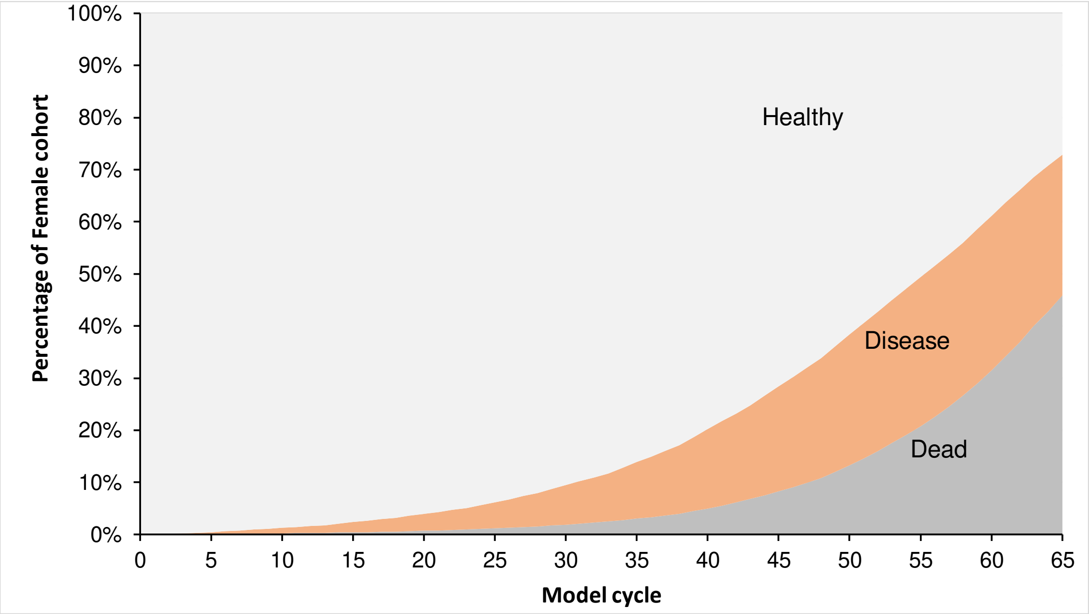

Figure S2: Cohort profile (female)

Gc VS, *et al. BMJ Open* 2019; 9:e027566. doi: 10.1136/bmjopen-2018-027566

2. Prevalence of physical activity levels by age group and gender

Figure S3: Physical activity levels by age group in Male (HSE 2012 data vs no intervention 'control')

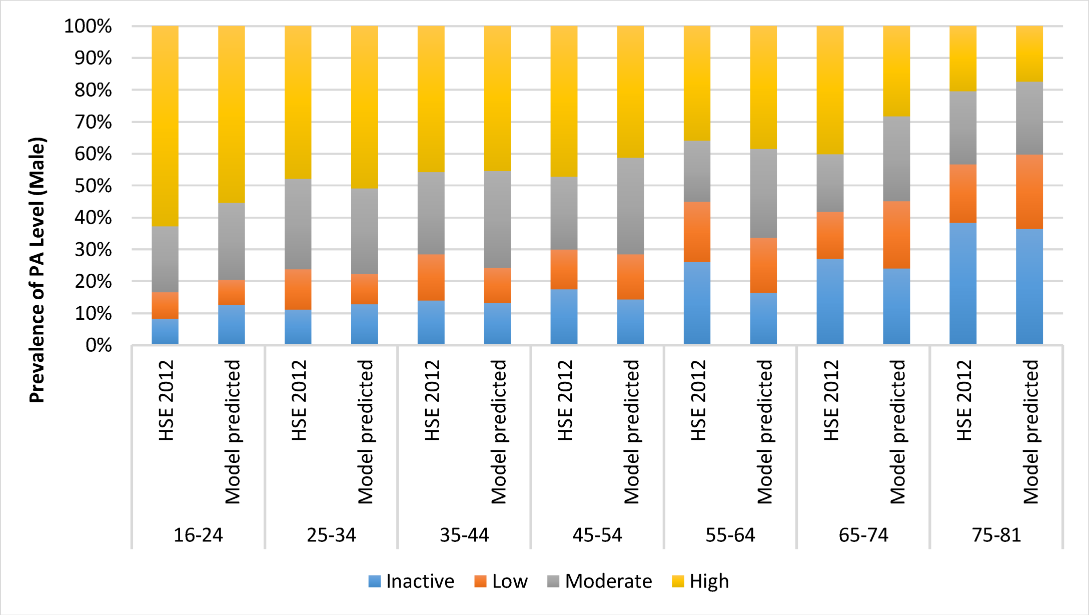

Figure S4: Physical activity levels by age group in Female (HSE 2012 data vs no intervention 'control')

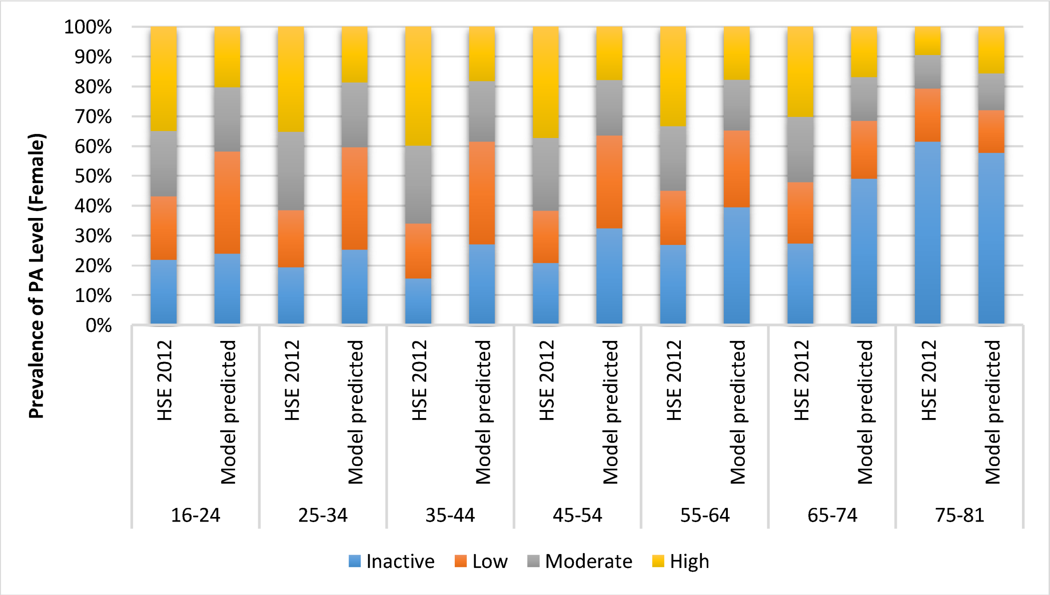

Gc VS, *et al. BMJ Open* 2019; 9:e027566. doi: 10.1136/bmjopen-2018-027566

3. Risk of disease events

Figure S5: CHD incidence: model predicted versus data informing model parameter by age-group and gender

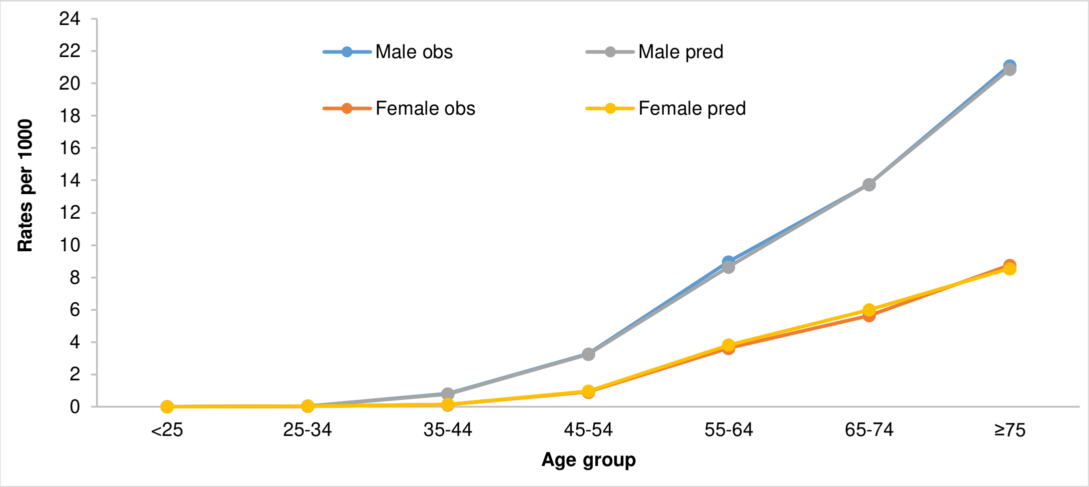

Figure S6: Stroke incidence: model predicted versus data informing model parameter by age-group and gender

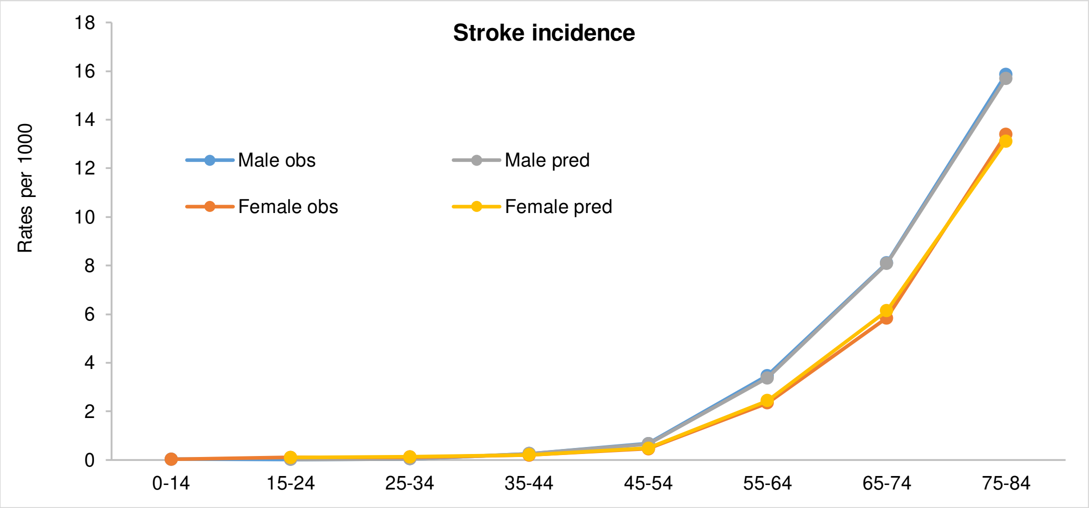

Gc VS, *et al. BMJ Open* 2019; 9:e027566. doi: 10.1136/bmjopen-2018-027566

Figure S7: Heart failure incidence: model predicted versus data informing model parameter by age-group and gender

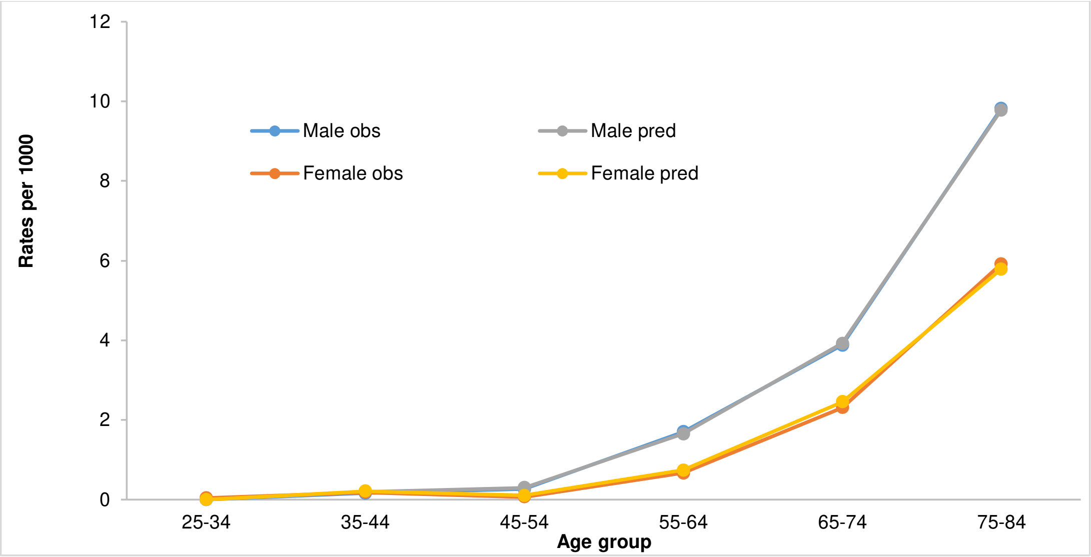

Figure S8: Incidence of type 2 diabetes: model predicted versus data informing model parameter by age-group and gender

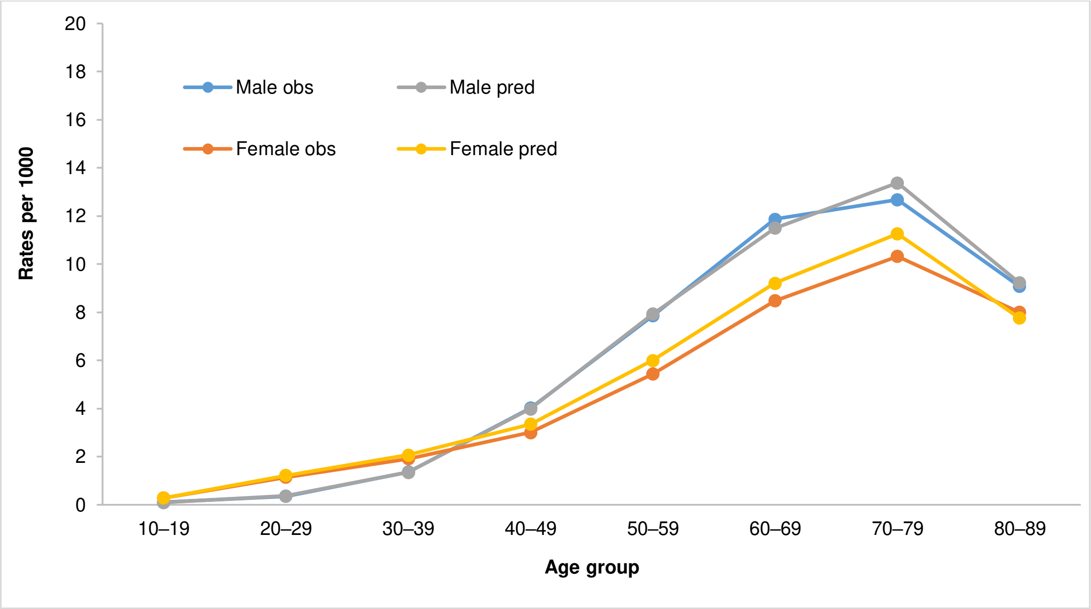

Gc VS, *et al. BMJ Open* 2019; 9:e027566. doi: 10.1136/bmjopen-2018-027566

Figure S9: Breast cancer (female) incidence: model predicted versus data informing model parameter by age-group and gender

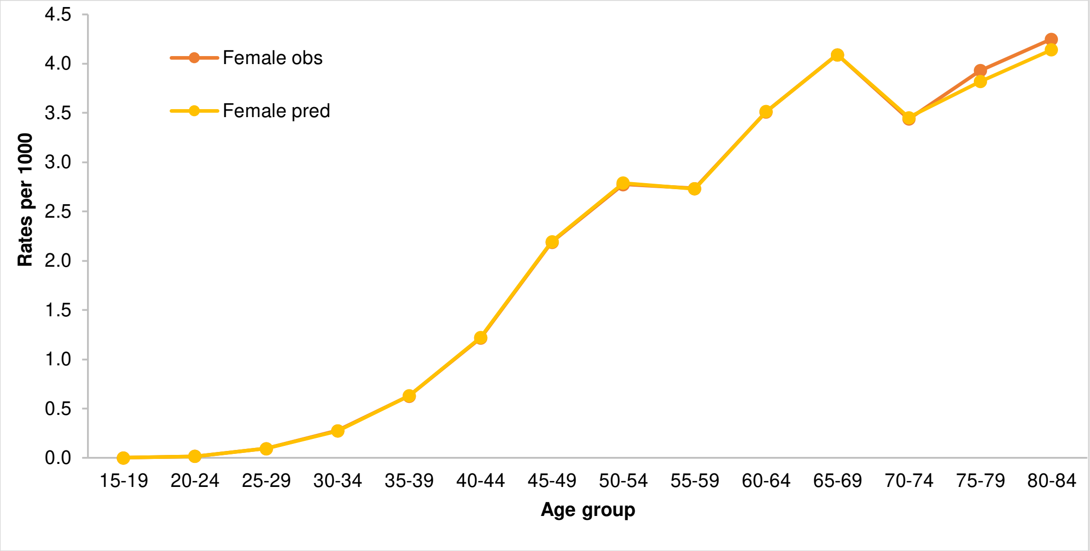

Figure S10: Colorectal cancer incidence: model predicted versus data informing model parameter by age-group and gender

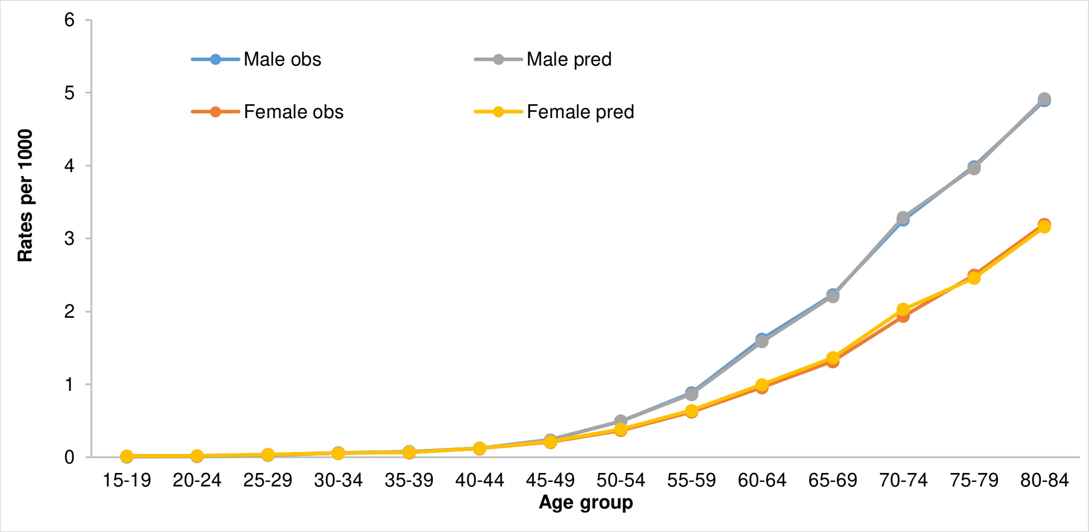

Gc VS, *et al. BMJ Open* 2019; 9:e027566. doi: 10.1136/bmjopen-2018-027566

References

1. Scholes S, Mindell J (2013) *Physical activity in children*. In: Craig R, Mindell J, editors. Health Survey for England 2012.(2013) London: The Health and Social Care Information Centre
2. Wahid A, Manek N, Nichols M *et al.* Quantifying the Association Between Physical Activity and Cardiovascular Disease and Diabetes: A Systematic Review and Meta-Analysis. *J Am Heart Assoc* 2016; 5.
3. Pandey A, Garg S, Khunger M *et al.* Dose-Response Relationship Between Physical Activity and Risk of Heart Failure: A Meta-Analysis. *Circulation* 2015; 132: 1786-1794.
4. Smith AD, Crippa A, Woodcock J *et al.* Physical activity and incident type 2 diabetes mellitus: a systematic review and dose-response meta-analysis of prospective cohort studies. *Diabetologia* 2016.
5. Kyu HH, Bachman VF, Alexander LT *et al.* Physical activity and risk of breast cancer, colon cancer, diabetes, ischemic heart disease, and ischemic stroke events: systematic review and dose-response meta-analysis for the Global Burden of Disease Study 2013. *BMJ* 2016; 354: i3857.
6. Smolina K, Wright FL, Rayner M *et al.* Determinants of the decline in mortality from acute myocardial infarction in England between 2002 and 2010: linked national database study. *BMJ* 2012; 344: d8059.
7. Bamford J, Sandercock P, Dennis M *et al.* A prospective study of acute cerebrovascular disease in the community: the Oxfordshire Community Stroke Project 1981-86. 1. Methodology, demography and incident cases of first-ever stroke. *J Neurol Neurosurg Psychiatry* 1988; 51: 1373-1380.
8. Cowie MR, Wood DA, Coats AJ *et al.* Incidence and aetiology of heart failure; a population-based study. *Eur Heart J* 1999; 20: 421-428.
9. Sharma M, Nazareth I, Petersen I Trends in incidence, prevalence and prescribing in type 2 diabetes mellitus between 2000 and 2013 in primary care: a retrospective cohort study. *BMJ Open* 2016; 6: e010210.
10. Cancer Research UK. Breast cancer statistics. 2016. [accessed 26 October 2016.] Available from: http://www.cancerresearchuk.org/sites/default/files/cstream-node/cases_crude_f_breast_2.xls.
11. Cancer Research UK. Bowel cancer incidence. 2016. [accessed 26 October 2016.] Available from: http://www.cancerresearchuk.org/sites/default/files/cstream-node/cases_crude_bowel_2.xls.
12. Bamford J, Sandercock P, Dennis M *et al.* A prospective study of acute cerebrovascular disease in the community: the Oxfordshire Community Stroke Project--1981-86. 2. Incidence, case fatality rates and overall outcome at one year of cerebral infarction, primary intracerebral and subarachnoid haemorrhage. *J Neurol Neurosurg Psychiatry* 1990; 53: 16-22.

13. MacIntyre K, Capewell S, Stewart S *et al.* Evidence of improving prognosis in heart failure: trends in case fatality in 66 547 patients hospitalized between 1986 and 1995. *Circulation* 2000; 102: 1126-1131.

14. Turner D, Raftery J, Cooper K *et al.* The CHD challenge: comparing four cost-effectiveness models. *Value Health* 2011; 14: 53-60.

15. Bronnum-Hansen H, Davidsen M, Thorvaldsen P *et al.* Long-term survival and causes of death after stroke. *Stroke* 2001; 32: 2131-2136.

16. Seshasai SR, Kaptoge S, Thompson A *et al.* Diabetes mellitus, fasting glucose, and risk of cause-specific death. *N Engl J Med* 2011; 364: 829-841.

17. Office for National Statistics. Cancer survival in England - adults diagnosed. 2016. [accessed 26 October 2016.] Available from: http://www.ons.gov.uk/file?uri=/peoplepopulationandcommunity/healthandsocialcare/conditionsanddiseases/datasets/cancersurvivalratescancersurvivalinenglandadultsdiagnosed/20102014/finalreferencetablev2.xls.

Gc VS, *et al. BMJ Open* 2019; 9:e027566. doi: 10.1136/bmjopen-2018-027566
