# Supplementary material for: Cost-effectiveness of physical activity interventions in adolescents: model development and illustration using two exemplar interventions
Source: BMJ Open. 2019 Aug 18;9(8):e027566. doi: 10.1136/bmjopen-2018-027566 (PMC6701678; doi:10.1136/bmjopen-2018-027566)
Supplement: Author's manuscript [file bmjopen-2018-027566.draft_revisions.pdf]

BMJ Open

BMJ Open is committed to open peer review. As part of this commitment we make the peer review history of every article we publish publicly available.

When an article is published we post the peer reviewers' comments and the authors' responses online. We also post the versions of the paper that were used during peer review. These are the versions that the peer review comments apply to.

The versions of the paper that follow are the versions that were submitted during the peer review process. They are not the versions of record or the final published versions. They should not be cited or distributed as the published version of this manuscript.

BMJ Open is an open access journal and the full, final, typeset and author-corrected version of record of the manuscript is available on our site with no access controls, subscription charges or pay-per-view fees (http://bmjopen.bmj.com).

If you have any questions on BMJ Open's open peer review process please email info.bmjopen@bmj.com

BMJ Open

An economic evaluation of interventions to improve physical activity in adolescents: a modelling study

Journal:	*BMJ Open*
Manuscript ID	bmjopen-2018-027566
Article Type:	Research
Date Submitted by the Author:	29-Oct-2018

Complete List of Authors:	Gc, Vijay; University of York Centre for Health Economics, ; University of East Anglia Norwich Medical School,<br>Suhrcke, Marc; University of York Centre for Health Economics,<br>Atkin, Andrew; University of East Anglia Faculty of Medicine and Health Sciences,<br>van Sluijs, Esther; University of Cambridge, MRC Epidemiology Unit<br>Turner, David; University of East Anglia, Public Health and Primary Care

Keywords:	physical activity, young adult, HEALTH ECONOMICS, cost-effectiveness

SCHOLARONE™
Manuscripts

An economic evaluation of interventions to improve physical activity in adolescents: a modelling study

Vijay S Gc[1,2], Marc Suhrcke[2], Andrew J Atkin[3,4], Esther van Sluijs[3], David Turner[1]

[1] Norwich Medical School, University of East Anglia, Norwich, UK

[2] Centre for Health Economics, University of York, York, UK

[3] MRC Epidemiology Unit and UKCRC Centre for Diet and Activity Research (CEDAR), University of Cambridge School of Clinical Medicine, Cambridge, UK

[4] School of Health Sciences, University of East Anglia, Norwich, UK

Corresponding author:

Mr Vijay S Gc, MPH

Centre for Health Economics

University of York

York, YO10 5DD

United Kingdom

Phone: +44 (0)1904 321973, Email: vijay.gc@york.ac.uk

Keywords: physical activity; adolescent; young adult; cost-effectiveness; economic model

Word count excluding title page, abstract, references, figures and tables: *3,617*

Number of tables and figures: Figures *3*, Tables *3*

For peer review only

For peer review only - http://bmjopen.bmj.com/site/about/guidelines.xhtml

Abstract

Objective: To develop a model to assess the long-term costs and health outcomes of physical activity interventions targeting adolescents.

Design: A Markov cohort simulation model was constructed with the intention of being capable of estimating long-term costs and health impacts of changes in activity levels during adolescence. The model parameters were informed by published literature, and the analysis took a National Health Service perspective over a lifetime horizon. Univariate and probabilistic sensitivity analyses were undertaken.

Setting: Schools and community

Participants: A hypothetical cohort of adolescents aged 16 years.

Interventions: Two exemplar school-based: a comparatively simple, after-school intervention and a more complex multi-component intervention compared to usual care.

Primary and secondary outcome measures: Incremental cost-effectiveness ratio as measured by cost per quality-adjusted life year gained.

Results: The model gave plausible estimates of the long-term effect of changes in physical activity. The use of two exemplar interventions suggests that the model could potentially be used to evaluate a number of different physical activity intervention in adolescents. The key model driver was the degree to which intervention effects were maintained over time.

Conclusions: The model developed here has the potential to assess long-term value for money of physical activity interventions in adolescents. The two applications of the model indicate that complex interventions may not necessarily be the ones considered the most cost-effective when longer-term costs and consequences are taken into account.

For peer review only - http://bmjopen.bmj.com/site/about/guidelines.xhtml
For peer review only

Word count: 228 words

Article summary

Strengths and limitations of this study

- A Markov cohort model was developed based on currently available evidence to simulate the long-term impacts in terms of costs and quality-adjusted life years of physical activity interventions for adolescents.
- The study incorporates the most recent evidence on the effect of increased physical activity in long-term chronic disease conditions.
- The model builds on previously published cohort models and includes additional health states. In addition, extensive sensitivity analyses have been performed to reflect uncertainty in model structure and parameter assumptions.
- A limitation of the present study is that the change in activity level over time were estimated using population-level prevalence data due to unavailability of longitudinal data describing the lifetime trajectory of physical activity and exclusion of long-term impacts on other conditions, for example, mental health.

For peer review only

For peer review only - http://bmjopen.bmj.com/site/about/guidelines.xhtml

Introduction

Insufficient physical activity is a key risk factor for chronic diseases, such as cardiovascular disease (CVD), type 2 diabetes, and some types of cancer, and in the general population.[1,2] Physical activity in young people is associated with many health benefits including improved cardiovascular and mental health,[3,4] academic performance[5] and bone health.[6] Whilst physical activity typically declines with age, active children are more likely to become active adults.[7,8] Although the short- and long-term health benefits of physical activity are well-documented,[9,10] in England, nearly half of all young people fail to achieve the recommended levels of physical activity, based on self-reports.[11,12] When measured objectively using accelerometers, the prevalence of inactivity is higher still (91% boys and 98% girls).[13]

The high prevalence of physical inactivity in young people places a significant burden on health care services and the wider economy. A 2014 report estimated a lifetime cost of £53.3 billion related to inactivity among today's 11 to 25 year olds,[12] taking into consideration the fact that physical activity levels in childhood predict adult activity levels.[14] This estimate includes direct healthcare costs of treating the burden of type 2 diabetes, chronic heart disease, stroke and colon cancer, and the risk of premature death and morbidity associated with these illnesses.

In recent years, there has been increasing interest in identifying interventions to improve young people's activity levels. Although some school-based physical activity interventions show promising effects[15,16] the existing evidence is very limited in both quantity and quality.[17] While improvements in physical activity may have long-term health benefits, the evidence on the longer-term costs and health benefits of interventions in adolescence is particularly sparse. Trials generally do not have sufficient follow-up to capture associated longer-term costs and consequences directly.[18] Therefore, to assess whether these interventions are an efficient use of limited healthcare resources, a long-term simulation model is required.

For peer review only - http://bmjopen.bmj.com/site/about/guidelines.xhtml

In this paper, we begin to fill this critical gap by, first, developing a purpose-built decision analytic model aimed at quantifying the potential long-term costs and quality-adjusted life-year (QALY) implications of changes in activity levels during adolescence. Second, to illustrate some of the practical implications of taking a longer-term perspective, we apply the model to two exemplar intervention programmes.

Methods

We developed a probabilistic, age- and gender-dependant state-transition Markov model to simulate a cohort of healthy adolescents. The model estimates the risk of cardiovascular, type 2 diabetes and oncological events over a lifetime, and associated costs and quality of life. The model structure was based on the previous published models[19,20] that assessed the cost-effectiveness of physical activity interventions in the adult population. The model combines information from a variety of sources relating to disease and physical activity epidemiology, mortality, effectiveness, health-related quality of life, and costs.

Structuring the model

Model and population type

A simulated cohort of 10,000 healthy adolescents aged 16 entered the model. The intervention is assumed to have been delivered at the start of the first model cycle. At the end of the first cycle, based on the intervention effectiveness evidence, a proportion of cohort members move to a higher activity level. Depending on the sustainability of the intervention effect, in subsequent years cohort members obtain an annual probability of remaining at the new activity level or moving to a lower physical activity state or to a disease state or death.

For peer review only - http://bmjopen.bmj.com/site/about/guidelines.xhtml

Model states

The health states included in the model are 'healthy' (disease-free), 'having a chronic disease' and 'dead'. At the beginning of the simulation, we assumed that all cohort members start out as healthy, i.e. disease free. Within the model, physical activity is classified into four activity levels (inactive, low, moderate and high) based on weekly moderate-to-vigorous physical activity (MVPA). The model has 11 health states in total: four physical activity levels, six chronic disease conditions and death (Figure 1). Among the healthy, the risk of developing one of the six diseases depends on age, gender and activity level. For simplicity, we assumed that health states included in the model were mutually exclusive, and cohort members did not move between disease states.

The selection of the disease conditions was based on currently available evidence describing the association between physical activity and disease risk,[21-26] and economic and health burden of diseases related to physical inactivity in the UK.[27] Disease conditions are all associated with costs and impact upon quality of life. For each of the selected exemplar interventions we re-ran the model changing appropriate data to reflect the costs and effectiveness of these interventions. Costs and QALYs were discounted at 3.5% as recommended for the National Institute for Health and Care Excellence (NICE) reference case.[28]

Time horizon

The time horizon of the model is 65 years, i.e. the model follows the cohort of 16 year olds until they reach 81 years – the average life expectancy in the UK.[29] A half-cycle correction was applied under the assumption that each transition happened halfway during the cycle.[30]

For peer review only - http://bmjopen.bmj.com/site/about/guidelines.xhtml
For peer review only

Populating the model

Baseline population and activity levels

Data on age and gender distribution of the initial population were obtained from the Office for National Statistics (ONS).[31] An estimate of baseline activity level (weekly MVPA) was taken from the 2012 Health Survey for England (HSE).[32] Participants were divided into four levels (<30, 30-149, 150-420 and ≥421 MVPA min per week) of activity by age and gender, based on the UK Department of Health's physical activity recommendations.[11] The moderate activity category equates to the current recommended level of physical activity.

Transition probabilities

To estimate the annual probability of developing each disease, the annual incidence rates for the disease conditions included in the model were taken from population-based studies in the UK.[33-39] These are probabilities for the general adult population and included all four activity levels. In order to adjust the differential risk of developing these disease conditions by activity level, we first derived the probability of developing that disease among inactive people, using the method presented by Hurley et al.[40] The probabilities for each condition among low, moderate and high activity levels in the cohort were estimated by multiplying the probabilities for the inactive population by corresponding relative risks (RRs) for low, moderate and high activity.[21-24,26] Supplementary Table S1 provides the transitional parameters**.**

Mortality rates

All-cause mortality rates by age and gender were derived from the ONS.[29] Mortality consists of disease-specific mortality and mortality due to other causes. We estimated other-cause mortality by subtracting the total number of deaths due to the six disease conditions included in the model from the all-cause mortality total. The other-cause mortality rates by age and gender were assumed constant in

For peer review only - http://bmjopen.bmj.com/site/about/guidelines.xhtml

the sensitivity analysis. The model assumes that a given proportion of coronary heart disease (CHD), stroke and heart failure (HF) events would be immediately fatal and people who survived one of these events had an increased subsequent risk of death (Supplementary Table S2). Case fatality rates for these health states were taken from published population-based studies in the UK.[41-43]

Individuals with type 2 diabetes were assigned an increased risk of mortality using data from a published meta-analysis.[44] Based on standardised mortality ratios reported in long-term follow-up studies of first-ever patients' stroke, a 2-fold increase in the risk of death after one year[45] was applied to the general mortality rates from the life tables to reflect the higher mortality burden post-vascular event. Annual mortality rates following first HF event were estimated from ten-year case fatality rates in patients admitted with a principal diagnosis of HF in Scotland.[43] The age-adjusted five-year net survival rates from the ONS[46] were used to estimate an annual risk of cancer death. It was assumed that the mortality rates do not increase due to cancer beyond five years after cancer diagnosis.

Interventions

We reviewed the literature to identify physical activity interventions targeting the adolescent population. For primarily illustrative purposes, we selected two interventions to test the model and explore the health and economic impact of smaller and greater changes in physical activity. The first was a more simple after-school intervention, not costly but likely with smaller benefits[47], the second a more complex, multi-component intervention – more costly intervention but with higher expected benefits[15]:

After school intervention programme. Mears and Jago[47] included six after-school interventions in their review and reported the pooled mean difference of 4.84 (-0.94 to 10.61) min of MVPA per day. These programmes typically included structured or unstructured play, planned MVPA, single or

For peer review only - http://bmjopen.bmj.com/site/about/guidelines.xhtml

multisport physical activity programme or adhering to specific instructions (e.g. maintaining sufficient intensity of exercise during a session).

Multi-component intervention. The intervention effect for school-based multi-component intervention was taken from a cluster-randomised trial[15] implemented in secondary schools in Australia. The intervention included multiple intervention strategies (e.g. active physical education lessons, enhanced school sport, supportive school physical activity policies) targeting physical activity. The reported difference in min of MVPA per day between the intervention and control arm at follow-up was 7.0 (2.7 to 11.4).

Costs, currency, price date and conversion

The annual costs incurred in each disease health state were based on previously published studies (Table 1). First-year costs and subsequent year costs are assigned for each of the health states modelled. Costs of CHD and stroke were taken from the statins health technology assessment (HTA).[48] Costs for HF were taken from the UKPDS study.[49] Costs for type 2 diabetes were based on Diabetes Glycaemic Education and Monitoring (DiGEM) trial and included medication and other healthcare costs.[50] Cost for breast cancer was taken from a screening appraisal for breast cancer.[51] The estimated cost is the weighted average treatment costs depending on the prognosis at diagnosis. Cost for colorectal cancer was based on a screening appraisal.[52] The appraisal reported the lifetime cost of colorectal cancer according to the cancer stage, and a weighted average cost was estimated using the proportion of cancers identified at each stage. All the costs were inflated to 2013-14.[53]

Health State Utility Values

Utility weights were used to value a year spent in each of the health states used in the model. A value of 1 means that the health state would be equivalent to full health and one year in that state would generate 1 QALY. For example, if an individual spent ten years in the CHD state with a utility of 0.65,

For peer review only - http://bmjopen.bmj.com/site/about/guidelines.xhtml
For peer review only

they would accrue 6.5 QALYs. The same number of QALYs could be generated by spending 6.5 years in a health state of 1. The lower the utility value, the worse the health state is considered to be. The weights used to value disease health states are given in Table 1 and were taken from Sullivan et al.[54], who used UK based community preferences to derive EQ-5D scores from the Medical Expenditure Panel Survey (MEPS). Utility weights for activity level by age- and gender were extracted from the HSE 2012 (Table 2).[55]

Table 1: Health state utilities and costs used in the model

Parameter	Value	Standard error	Distribution	Source
Health state utilities				[54]
CHD	0.65	0.0203	Beta (α=357, β=191)	
Stroke	0.52	0.0192	Beta (α=355, β=323)	
Heart failure	0.49	0.0194	Beta (α=326, β=335)	
Type 2 diabetes	0.66	0.0054	Beta (α=5032, β=2548)	
Breast cancer	0.76	0.0133	Beta (α=791, β=256)	
Colorectal cancer	0.67	0.0314	Beta (α=150, β=73)	
**Costs of health states**				
CHD 1st event	£5,562	£556	Gamma (α=100, β=56)	[48]
CHD subsequent	£214	£21	Gamma (α=100, β=2)	[48]
CHD fatal	£1,458	£146	Gamma (α=100, β=15)	[48]
Stroke 1st event	£10,062	£1,006	Gamma (α=100, β=101)	[48]
Stroke subsequent	£2,705	£270	Gamma (α=100, β=27)	[48]
Stroke fatal	£8,805	£881	Gamma (α=100, β=88)	[48]

For peer review only - http://bmjopen.bmj.com/site/about/guidelines.xhtml

Heart failure	£2,402	£240	Gamma (α=100, β=24)	[49]
HF subsequent	£815	£82	Gamma (α=100, β=8)	[49]
Type 2 diabetes	£1,257	£126	Gamma (α=100, β=13)	[50]
Breast cancer	£12,155	£1,215	Gamma (α=100, β=122)	[51]
Colorectal cancer	£16,978	£1,698	Gamma (α=100, β=170)	[52]

For peer review only

Table 2: Baseline utilities associated with physical activity level

Age-group	Utility values by activity level				Distribution	Source
	Inactive	Low	Moderate	High		
16-34	0.897	0.918	0.937	0.943	beta	[55]
35-44	0.770	0.889	0.914	0.927	beta	[55]
45-54	0.696	0.852	0.899	0.921	beta	[55]
55-64	0.648	0.861	0.863	0.907	beta	[55]
65-74	0.657	0.823	0.870	0.897	beta	[55]
65-74	0.701	0.829	0.850	0.876	beta	[55]

Inactive: <30; Low: 30-149; Moderate: 150-420 and High: ≥421 MVPA min per week

Modelling health benefits

We estimated the probability of moving to a higher activity level after intervention by adding intervention-specific MVPA minutes to baseline levels. We then calculated the proportion of cohort members that moved from one activity level to another, and we used this proportion as a transition

For peer review only - http://bmjopen.bmj.com/site/about/guidelines.xhtml

probability. Members of the cohort who improved physical activity level at the end of cycle 0 were assumed to have a lower probability of developing CHD, stroke, HF or any of the cancers.

Estimation of sustainability of intervention effect

The decline in activity levels over time in the 'no intervention'-group was modelled based on data from a recent meta-analysis examining the change in activity level from adolescence to adulthood[8] as well as using prevalence data from the 2012 HSE. The meta-analysis showed a decrease of 6.5 min/day of MVPA in boys and 5.5 min/day of MVPA in girls from adolescence to adulthood. As the review included studies reporting at least one measurement between both 13-19 years and 16-30 years, we assumed that the decrease in MVPA minutes was for a seven-year period. For the intervention group, a 50% decline in intervention effect per year post-intervention was assumed. Therefore, the activity levels decreased towards the control activity level after seven years. The effect of this was that a number of individuals in the intervention group were re-categorised into higher activity groups immediately after the intervention. Over time, many of these individuals would fall back into lower activity groups, and at the end of seven-year, there was no real difference between intervention and control groups. To account for the decline in physical activity occurring with age across the life course, we estimated age-related activity levels using 2012 HSE data on physical activity prevalence by age and gender. Activity levels were estimated in three broad age groups: 24-44, 45-64 and ≥65 years to reflect activity-level differences in adulthood, middle-age and retirement.

Estimation of costs of intervention

Costs of delivering after-school intervention were taken from a cluster randomised feasibility study in the UK.[56] The authors reported cost estimates (£49 per participant, 2012-13 price) of a teaching assistant led extra-curricular physical activity intervention. We took that as an indicative cost of the after-school intervention, inflated to 2014 prices (£51 per participant). The cost includes intervention

For peer review only - http://bmjopen.bmj.com/site/about/guidelines.xhtml

delivery costs, one-off training and non-recurrent costs such as consultation and intervention development work.

Sutherland et al. performed an economic evaluation alongside the multi-component intervention [57] and reported that the intervention costed AU$ 394 per participant. This cost includes opportunity costs for delivery of strategies by school staff and community sport and fitness providers, materials and printing. We converted this cost to 2014 pound sterling (£190 per participant) by applying the gross domestic product deflator index (GDP values) and purchasing power parities conversion rates using the CCEMG-EPPI-Centre Cost Converter (V1.5).[58] We added the intervention cost in the first year for the intervention groups.

Validation

The model structure, data sources and the effectiveness evidence used in the exemplar interventions and model results were validated by the study team comprising health economists, behavioural epidemiologists and trialists. Internal validity of the model code was ensured using several tests and by assuming a constant total population throughout the calculations. Furthermore, model predictions were examined to make sure that results from the model were consistent with the model's specifications. We specifically checked lifetime incidence and mortality, as well as physical activity prevalence by age and gender. Details can be found in the validation section in the Supplemental Materials (Section B).

Cost-effectiveness analysis

We used the NICE reference case[28] and followed existing guidelines for modelling.[59] The analysis was performed from the perspective of the NHS and personal social services. Costs and health outcomes were discounted at 3.5% per year.[28] We estimated the cost-effectiveness ratio for each intervention compared to 'no intervention'. The incremental costs and QALYs gained by the intervention were estimated and averaged across the simulated cohort. The incremental cost-effectiveness ratio (ICER)

For peer review only - http://bmjopen.bmj.com/site/about/guidelines.xhtml
For peer review only

was estimated as a ratio between the additional expected cost of the intervention, and the additional expected QALYs gained, both relative to the 'no intervention' alternative. The intervention was considered cost-effective if the ICER was no more than the lower NICE recommended threshold of £20,000 per QALY. The uncertainty surrounding the estimates of cost-effectiveness is presented using cost-effectiveness acceptability curves (CEACs). A CEAC shows the probability that an intervention is cost-effective compared with alternative intervention for a range of cost-effectiveness thresholds.[60]

Sensitivity analyses

We performed sensitivity analyses by changing intervention decay rates and time horizon that affect cost-effectiveness results. Deterministic one-way, scenario and extreme value analyses were undertaken. This was complemented by probabilistic sensitivity analysis (PSA) to assess the combined effects of uncertainty in the input parameters by simultaneously sampling input parameter values from within a specified distribution using Monte Carlo simulations (2,000 iterations). Uncertainty about the sustainability of the intervention effects was assessed by varying the decay rates between 0% and 100%. In our base-case analysis, we assumed that the intervention effects are sustained for the first year but decay exponentially at a rate of 50% per annum thereafter, resulting in virtually no intervention effect after five years.

Probabilities of disease events and utilities were assumed to follow a beta distribution, costs followed a gamma distribution and risk reductions/hazard ratios a lognormal distribution.[61] The model was developed and implemented in Microsoft Excel.

Patient and public involvement

Public involvement informed the questions addressed in the overarching research project of which this study is a part. No further public involvement was sought with regards to the development of the research question, the outcome measures or the study design.

For peer review only - http://bmjopen.bmj.com/site/about/guidelines.xhtml
For peer review only

Results

Base-case analysis

In our base-case analysis, both after-school and multi-component interventions were associated with higher costs and were more effective than no intervention (

Table 3). The multi-component intervention was associated with a QALY gain of 0.002 at an incremental cost of £138, compared to the after-school intervention, yielding an ICER of £68,056.

Table 3: Incremental cost-effectiveness ratios in the base-case and sensitivity analyses

	Total cost (£)	Total QALYs	Incremental cost (£)	Incremental QALY	ICER
**Base-case analysis**					
No intervention	4,441	21.705	–	–	–
After-school intervention	4,491	21.710	50.64	0.004	11,486
Multi-component intervention	4,629	21.712	137.89	0.002	68,056
**No (0%) decay of intervention effects**					
No intervention	4,437	21.707	–	–	–
After-school intervention	4,451	21.898	14.32	0.191	75
Multi-component intervention	4,571	21.987	119.59	0.089	1,342
**33% decay of intervention effects**					
No intervention	4,430	21.705	–	–	–
After-school intervention	4,479	21.718	48.86	0.013	3,661
Multi-component intervention	4,616	21.726	136.94	0.008	17,661
**100% decay of intervention effects**					
No intervention	4,432	21.705	–	–	–
After-school intervention	4,484	21.707	51.42	0.002	28,838
Multi-component intervention	4,621	21.708	137.61	0.001	189,897

For peer review only - http://bmjopen.bmj.com/site/about/guidelines.xhtml

	Total cost (£)	Total QALYs	Incremental cost (£)	Incremental QALY	ICER
**Time horizon (10 years)**					
No intervention	41	7.167	–	–	–
After-school intervention	92	7.170	51.47	0.004	14,204
Multi-component intervention	231	7.172	138.60	0.002	75,100
**Time horizon (20 years)**					
No intervention	219	12.835	–	–	–
After-school intervention	270	12.838	51.09	0.004	13,414
Multi-component intervention	408	12.840	138.19	0.002	72,346

ICER, incremental cost-effectiveness ratio (incremental cost/incremental QALY); QALY, quality-adjusted life year.

Sensitivity analyses

Sensitivity analyses were implemented, varying the base-case assumptions and inputs, as outlined in the methods section (

Table 3). Both the after-school and multi-component interventions had more favourable ICERs at lower decay rates, indicating that cost-effectiveness of physical activity interventions depends on the sustainability of intervention effects over time. The results of PSA are presented on the cost-effectiveness plane (Figure 2), with simulations found to lie predominantly in the northeast quadrant indicating – as expected – an improved health outcome but also at higher spending on physical activity interventions.

Figure 3 depicts the probability of the interventions being cost-effective. At a threshold value of £20,000 per QALY gained, the after-school intervention has the highest probability of being cost-effective (59%) while for the multi-component intervention this probability is at 8%.

For peer review only

For peer review only - http://bmjopen.bmj.com/site/about/guidelines.xhtml

Discussion

Main findings

We found that modelling the long-term effects of physical activity among adolescent is feasible and that early interventions probably make only small differences to lifetime costs and QALYs. Although more complex and costly interventions may have bigger health gains, that need not mean they are automatically better value for money in the longer-term. Our results underline that cost-effectiveness estimates are critically sensitive to assumptions around sustainability of intervention effects. Our base-case analysis assumed that intervention effects decay exponentially at a rate of 50% per year post-intervention, such that intervention group participants revert towards the control activity levels at the end of a seven-year period.

Comparison with previous models

Two previous UK-based modelling studies evaluating the cost-effectiveness of community based physical activity interventions included young people. Although Frew et al[20] used the same basic modelling approach, their model included only three activity categories and study participants included both young people and adults (16-70 year olds). By contrast, our model has four physical activity categories, included two more health states and health effects are modelled over a lifetime (with a time horizon of 65 years). Pringle et al[62] evaluated seven broad categories of community based physical activity interventions (one of which was related to the interventions considered here). Their analysis was based on the NICE/Matrix model[63] which used two activity categories (active or inactive) with 4 disease states. However, they did not focus on young people. Recently, Lee and colleagues[64] modelled the economic and health impact of increasing children's physical activity in the US. However, unlike in the current model, their model specifically looked at the influence of physical activity on weight status

For peer review only - http://bmjopen.bmj.com/site/about/guidelines.xhtml

For peer review only

and metabolic profiles, and ignored decay in intervention effects or the naturally occurring decline in physical activity associated with ageing.

Strengths and limitations

Although our model used a similar modelling framework as previous models,[19,20] we included additional health states and focused on adolescent physical activity interventions – this approach has hitherto been neglected, despite the potential importance of intervening at this key stage. We also include the most up to date available evidence on disease conditions. UK-specific incidence rates were used to ensure that patients entering the model match the likely distribution of events in the UK. We chose not to include sedentary behaviour, as there is ongoing debate around its impact on health independent of physical activity.[65]

As with all models, assumptions were required for the analysis. We included six disease conditions that have established links with physical (in)activity. This might underestimate the potential impact of physical activity on other disease conditions, most notably mental health. The effect of physical activity on prevention of depression is still a subject of debate[66], and a clear dose-response relationship between physical activity and reduced depression is not readily apparent.[67] Further empirical evidence is required to facilitate its inclusion in a future iteration of the model. The current model does not allow for transitions between disease states as this requires more complex modelling. However, this may underestimate the potential impact of physical activity. For example, participants with type 2 diabetes tend to have a higher risk of developing cardiovascular conditions.[68] Although the intervention was aimed at adolescents, due to the nature of the disease conditions included in the model, it would mainly be older adults who develop these diseases.

Our assumption on intervention decay rates, which was based on previous modelling studies,[19,69,70] would mean that there would be very little difference in activity between groups at time points when

For peer review only - http://bmjopen.bmj.com/site/about/guidelines.xhtml

individuals are starting to develop these diseases. We tested different assumptions on maintenance of intervention effect to examine influence in cost-effectiveness results. Further research into maintenance of intervention effect would provide valuable information.

Conclusion

Interventions to promote physical activity among adolescents represent a potentially promising public health measure to reduce the burden of cardiovascular and other non-communicable diseases. Faced with limited resources, governments need to carefully weigh the costs of any proposed interventions against the associated health benefits expected to be realised over the longer term, in order to ensure that net health gains are maximised. The model developed here has potential to assess the long-term, beyond trial duration, value-for-money of such interventions. The two purely illustrative applications of the model convey the notion that complex, resource intensive interventions may not necessarily be the ones considered the better buys compared to cheaper, more targeted ones.

Author contributions

VG designed the model, performed analysis and wrote the first draft of the manuscript. DT and MS supervised the process and provided input into interpretation of results. AA and EvS provided critical comments on model structure and data analysis. All authors contributed to the critical revision of the manuscript and approved the final version of the manuscript.

Funding

This report is an independent research commissioned and funded by the Department of Health Policy Research Programme (opportunities within the school environment to shift the distribution of activity intensity in adolescents, PR-R5-0213-25001). The views expressed in this publication are those of the

For peer review only - http://bmjopen.bmj.com/site/about/guidelines.xhtml

author(s) and not necessarily those of the Department of Health. This work was also supported by the Medical Research Council (unit programme number: MC_UU_12015/7). The work was undertaken under the auspices of the Centre for Diet and Activity Research (CEDAR), a UKCRC Public Health Research Centre of Excellence which is funded by the British Heart Foundation, Cancer Research UK, Economic and Social Research Council, Medical Research Council, the National Institute for Health Research, and the Wellcome Trust (MR/K023187/1).

Availability of data and material

The model was developed using data from publicly available sources and all the model inputs are described in the paper.

Competing interests statement

Nothing to report.

Ethics approval and consent to participate

Not applicable.

Acknowledgements

We thank Dr Katie Morton and Dr Kirsten Corder for their comments in the early stage of model development work.

For peer review only - http://bmjopen.bmj.com/site/about/guidelines.xhtml

References

1. Reiner M, Niermann C, Jekauc D *et al.* Long-term health benefits of physical activity--a systematic review of longitudinal studies. *BMC Public Health* 2013; 13: 813.

2. Lee IM, Shiroma EJ, Lobelo F *et al.* Effect of physical inactivity on major non-communicable diseases worldwide: an analysis of burden of disease and life expectancy. *Lancet* 2012; 380: 219-229.

3. Ekelund U, Luan J, Sherar LB *et al.* Moderate to vigorous physical activity and sedentary time and cardiometabolic risk factors in children and adolescents. *JAMA* 2012; 307: 704-712.

4. Biddle SJ, Asare M Physical activity and mental health in children and adolescents: a review of reviews. *Br J Sports Med* 2011; 45: 886-895.

5. Singh A, Uijtdewilligen L, Twisk JW *et al.* Physical activity and performance at school: a systematic review of the literature including a methodological quality assessment. *Arch Pediatr Adolesc Med* 2012; 166: 49-55.

6. Hallal PC, Victora CG, Azevedo MR *et al.* Adolescent Physical Activity and Health. *Sports Medicine* 2006; 36: 1019-1030.

7. Telama R, Yang X, Viikari J *et al.* Physical activity from childhood to adulthood: a 21-year tracking study. *Am J Prev Med* 2005; 28: 267-273.

8. Corder K, Winpenny E, Love R *et al.* Change in physical activity from adolescence to early adulthood: a systematic review and meta-analysis of longitudinal cohort studies. *Br J Sports Med* 2017; doi:10.1136/bjsports-2016-097330.

9. Department of Health. *UK physical activity guidelines*. London: Department of Health, 2011.

10. Warburton DE, Nicol CW, Bredin SS Health benefits of physical activity: the evidence. *CMAJ* 2006; 174: 801-809.

11. Department of Health. *Start active, stay active: a report on physical activity from the four home countries' Chief Medical Officers*. Department of Health, 2011.

For peer review only - http://bmjopen.bmj.com/site/about/guidelines.xhtml

For peer review only

12. Evans K. The inactivity time bomb. The economic cost of physical inactivity in young people: a StreetGames/Cebr report. 2014. [accessed 13 January 2017.] Available from: http://www.streetgames.org/sites/default/files/The-Inactivity-TimeBomb-StreetGames-Cebr-report-April-2014.pdf.

13. Cooper AR, Goodman A, Page AS *et al.* Objectively measured physical activity and sedentary time in youth: the International children's accelerometry database (ICAD). *Int J Behav Nutr Phys Act* 2015; 12: 113.

14. Telama R Tracking of physical activity from childhood to adulthood: a review. *Obes Facts* 2009; 2: 187-195.

15. Sutherland RL, Campbell EM, Lubans DR *et al.* The Physical Activity 4 Everyone Cluster Randomized Trial: 2-Year Outcomes of a School Physical Activity Intervention Among Adolescents. *Am J Prev Med* 2016; 51: 195-205.

16. Grydeland M, Bergh IH, Bjelland M *et al.* Intervention effects on physical activity: the HEIA study - a cluster randomized controlled trial. *Int J Behav Nutr Phys Act* 2013; 10: 17.

17. Borde R, Smith JJ, Sutherland R *et al.* Methodological considerations and impact of school-based interventions on objectively measured physical activity in adolescents: a systematic review and meta-analysis. *Obes Rev* 2017; 18: 476-490.

18. Buxton MJ, Drummond MF, Van Hout BA *et al.* Modelling in economic evaluation: an unavoidable fact of life. *Health Econ* 1997; 6: 217-227.

19. Roux L, Pratt M, Tengs TO *et al.* Cost effectiveness of community-based physical activity interventions. *Am J Prev Med* 2008; 35: 578-588.

20. Frew EJ, Bhatti M, Win K *et al.* Cost-effectiveness of a community-based physical activity programme for adults (Be Active) in the UK: an economic analysis within a natural experiment. *Br J Sports Med* 2014; 48: 207-212.

For peer review only - http://bmjopen.bmj.com/site/about/guidelines.xhtml

For peer review only

21. Wahid A, Manek N, Nichols M *et al.* Quantifying the Association Between Physical Activity and Cardiovascular Disease and Diabetes: A Systematic Review and Meta-Analysis. *J Am Heart Assoc* 2016; 5.

22. Pandey A, Garg S, Khunger M *et al.* Dose-Response Relationship Between Physical Activity and Risk of Heart Failure: A Meta-Analysis. *Circulation* 2015; 132: 1786-1794.

23. Smith AD, Crippa A, Woodcock J *et al.* Physical activity and incident type 2 diabetes mellitus: a systematic review and dose-response meta-analysis of prospective cohort studies. *Diabetologia* 2016.

24. Kyu HH, Bachman VF, Alexander LT *et al.* Physical activity and risk of breast cancer, colon cancer, diabetes, ischemic heart disease, and ischemic stroke events: systematic review and dose-response meta-analysis for the Global Burden of Disease Study 2013. *BMJ* 2016; 354: i3857.

25. Brenner DR, Yannitsos DH, Farris MS *et al.* Leisure-time physical activity and lung cancer risk: A systematic review and meta-analysis. *Lung Cancer* 2016; 95: 17-27.

26. Tardon A, Lee WJ, Delgado-Rodriguez M *et al.* Leisure-time physical activity and lung cancer: a meta-analysis. *Cancer Causes Control* 2005; 16: 389-397.

27. Allender S, Foster C, Scarborough P *et al.* The burden of physical activity-related ill health in the UK. *J Epidemiol Community Health* 2007; 61: 344-348.

28. National Institute for Health and Care Excellence. *Guide to the methods of technology appraisal 2013*. London: National Institute for Health and Care Excellence, 2013.

29. Office for National Statistics. National Life Tables: United Kingdom. 2016. [accessed 3 November 2016.] Available from: http://www.ons.gov.uk/file?uri=/peoplepopulationandcommunity/birthsdeathsandmarriages/lifeexpectancies/datasets/nationallifetablesenglandreferencetables/current/nlteng1315reg.xls.

For peer review only - http://bmjopen.bmj.com/site/about/guidelines.xhtml

30. Naimark DM, Bott M, Krahn M The half-cycle correction explained: two alternative pedagogical approaches. *Med Decis Making* 2008; 28: 706-712.

31. Office for National Statistics. MYE1: Population Estimates Summary for the UK, mid-2014. 2016. [accessed 20 October 2016.] Available from: https://www.ons.gov.uk/file?uri=/peoplepopulationandcommunity/populationandmigration/populationestimates/datasets/populationestimatesanalysistool/mid2014/analysistoolmid2014uk.zip.

32. Scholes S, Mindell J (2013) *Physical activity in children*. In: Craig R, Mindell J, editors. Health Survey for England 2012.(2013) London: The Health and Social Care Information Centre

33. Sutcliffe SJ, Fox KF, Wood DA *et al.* Incidence of coronary heart disease in a health authority in London: review of a community register. *BMJ* 2003; 326: 20.

34. Bamford J, Sandercock P, Dennis M *et al.* A prospective study of acute cerebrovascular disease in the community: the Oxfordshire Community Stroke Project 1981-86. 1. Methodology, demography and incident cases of first-ever stroke. *J Neurol Neurosurg Psychiatry* 1988; 51: 1373-1380.

35. Cowie MR, Wood DA, Coats AJ *et al.* Incidence and aetiology of heart failure; a population-based study. *Eur Heart J* 1999; 20: 421-428.

36. Sharma M, Nazareth I, Petersen I Trends in incidence, prevalence and prescribing in type 2 diabetes mellitus between 2000 and 2013 in primary care: a retrospective cohort study. *BMJ Open* 2016; 6: e010210.

37. Cancer Research UK. Lung cancer incidence. 2016. [accessed 26 October 2016.] Available from: http://www.cancerresearchuk.org/sites/default/files/cstream-node/cases_crude_lung_2.xls.

38. Cancer Research UK. Bowel cancer incidence. 2016. [accessed 26 October 2016.] Available from: http://www.cancerresearchuk.org/sites/default/files/cstream-node/cases_crude_bowel_2.xls.

39. Cancer Research UK. Breast cancer statistics. 2016. [accessed 26 October 2016.] Available from: http://www.cancerresearchuk.org/sites/default/files/cstream-node/cases_crude_f_breast_2.xls.

For peer review only - http://bmjopen.bmj.com/site/about/guidelines.xhtml

40. Hurley SF, Matthews JP The Quit Benefits Model: a Markov model for assessing the health benefits and health care cost savings of quitting smoking. *Cost Eff Resour Alloc* 2007; 5: 2.

41. Smolina K, Wright FL, Rayner M *et al.* Determinants of the decline in mortality from acute myocardial infarction in England between 2002 and 2010: linked national database study. *BMJ* 2012; 344: d8059.

42. Bamford J, Sandercock P, Dennis M *et al.* A prospective study of acute cerebrovascular disease in the community: the Oxfordshire Community Stroke Project--1981-86. 2. Incidence, case fatality rates and overall outcome at one year of cerebral infarction, primary intracerebral and subarachnoid haemorrhage. *J Neurol Neurosurg Psychiatry* 1990; 53: 16-22.

43. MacIntyre K, Capewell S, Stewart S *et al.* Evidence of improving prognosis in heart failure: trends in case fatality in 66 547 patients hospitalized between 1986 and 1995. *Circulation* 2000; 102: 1126-1131.

44. Seshasai SR, Kaptoge S, Thompson A *et al.* Diabetes mellitus, fasting glucose, and risk of cause-specific death. *N Engl J Med* 2011; 364: 829-841.

45. Bronnum-Hansen H, Davidsen M, Thorvaldsen P *et al.* Long-term survival and causes of death after stroke. *Stroke* 2001; 32: 2131-2136.

46. Office for National Statistics. Cancer survival in England - adults diagnosed. 2016. [accessed 26 October 2016.] Available from: http://www.ons.gov.uk/file?uri=/peoplepopulationandcommunity/healthandsocialcare/conditionsanddiseases/datasets/cancersurvivalratescancersurvivalinenglandadultsdiagnosed/20102014/finalreferencetablev2.xls.

47. Mears R, Jago R Effectiveness of after-school interventions at increasing moderate-to-vigorous physical activity levels in 5- to 18-year olds: a systematic review and meta-analysis. *Br J Sports Med* 2016.

For peer review only - http://bmjopen.bmj.com/site/about/guidelines.xhtml

48. Ward S, Lloyd Jones M, Pandor A *et al.* A systematic review and economic evaluation of statins for the prevention of coronary events. *Health Technol Assess* 2007; 11: 1-160, iii-iv.

49. Clarke P, Gray A, Legood R *et al.* The impact of diabetes-related complications on healthcare costs: results from the United Kingdom Prospective Diabetes Study (UKPDS Study No. 65). *Diabet Med* 2003; 20: 442-450.

50. Farmer AJ, Wade AN, French DP *et al.* Blood glucose self-monitoring in type 2 diabetes: a randomised controlled trial. *Health Technol Assess* 2009; 13: iii-iv, ix-xi, 1-50.

51. Madan J, Rawdin A, Stevenson M *et al.* A rapid-response economic evaluation of the UK NHS Cancer Reform Strategy breast cancer screening program extension via a plausible bounds approach. *Value Health* 2010; 13: 215-221.

52. Tappenden P, Chilcott J, Eggington S *et al.* Option appraisal of population-based colorectal cancer screening programmes in England. *Gut* 2007; 56: 677-684.

53. Curtis L, Burns A, editors (2015) Unit Costs of Health and Social Care 2015. Canterbury: Personal Social Services Research Unit, University of Kent.

54. Sullivan PW, Slejko JF, Sculpher MJ *et al.* Catalogue of EQ-5D scores for the United Kingdom. *Med Decis Making* 2011; 31: 800-804.

55. Craig R, Mindell J (2013) *Health Survey for England 2012*. In: Craig R, Mindell J, editors.(2013) London: The Health and Social Care Information Centre

56. Jago R, Sebire SJ, Davies B *et al.* Randomised feasibility trial of a teaching assistant led extracurricular physical activity intervention for 9 to 11 year olds: Action 3:30. *Int J Behav Nutr Phys Act* 2014; 11: 114.

57. Sutherland R, Reeves P, Campbell E *et al.* Cost effectiveness of a multi-component school-based physical activity intervention targeting adolescents: the 'Physical Activity 4 Everyone' cluster randomized trial. *Int J Behav Nutr Phys Act* 2016; 13: 94.

For peer review only - http://bmjopen.bmj.com/site/about/guidelines.xhtml
For peer review only

58. The Campbell and Cochrane Economics Methods Group (CCEMG) and the Evidence for Policy and Practice Information and Coordinating Centre (EPPI-Centre). CCEMG - EPPI-Centre Cost Converter (v1.5). [accessed 31 January 2017.] Available from: http://eppi.ioe.ac.uk/costconversion/default.aspx.

59. Siebert U, Alagoz O, Bayoumi AM *et al.* State-transition modeling: a report of the ISPOR-SMDM Modeling Good Research Practices Task Force--3. *Value Health* 2012; 15: 812-820.

60. Fenwick E, Marshall DA, Levy AR *et al.* Using and interpreting cost-effectiveness acceptability curves: an example using data from a trial of management strategies for atrial fibrillation. *BMC Health Serv Res* 2006; 6: 52.

61. Briggs A, Claxton K, Sculpher MJ (2006) *Chapter 4: Making decision models probabilistic*. Decision Modelling for Health Economic Evaluation.(2006): Oxford University Press. pp. 77-120.

62. Pringle A, Cooke C, Gilson N *et al.* Cost-effectiveness of interventions to improve moderate physical activity: A study in nine UK sites. *Health Education Journal* 2010; 69: 211-224.

63. Matrix Research and Consulting. *Modeling the cost effectiveness of physical activity interventions*. London: National Institute for Health and Clinical Excellence, 2006.

64. Lee BY, Adam A, Zenkov E *et al.* Modeling The Economic And Health Impact Of Increasing Children's Physical Activity In The United States. *Health Aff (Millwood)* 2017; 36: 902-908.

65. Pearson N, Braithwaite RE, Biddle SJ *et al.* Associations between sedentary behaviour and physical activity in children and adolescents: a meta-analysis. *Obes Rev* 2014; 15: 666-675.

66. Teychenne M, Ball K, Salmon J Physical activity and likelihood of depression in adults: a review. *Prev Med* 2008; 46: 397-411.

67. Mammen G, Faulkner G Physical activity and the prevention of depression: a systematic review of prospective studies. *Am J Prev Med* 2013; 45: 649-657.

For peer review only - http://bmjopen.bmj.com/site/about/guidelines.xhtml

68. Shah AD, Langenberg C, Rapsomaniki E *et al.* Type 2 diabetes and incidence of a wide range of cardiovascular diseases: a cohort study in 1.9 million people. *Lancet* 2015; 385 Suppl 1: S86.

69. Cobiac LJ, Vos T, Barendregt JJ Cost-effectiveness of interventions to promote physical activity: a modelling study. *PLoS Med* 2009; 6: e1000110.

70. Gc VS, Suhrcke M, Hardeman W *et al.* Cost-Effectiveness and Value of Information Analysis of Brief Interventions to Promote Physical Activity in Primary Care. *Value Health* 2018; 21: 18-26.

For peer review only

For peer review only - http://bmjopen.bmj.com/site/about/guidelines.xhtml

Figure title and legend

Figure 1: Conceptual overview of the model

Figure 2: Scatter plot of incremental costs and QALYs for each intervention, relative to no intervention

Figure 3: Cost-effectiveness acceptability curves

For peer review only

For peer review only - http://bmjopen.bmj.com/site/about/guidelines.xhtml

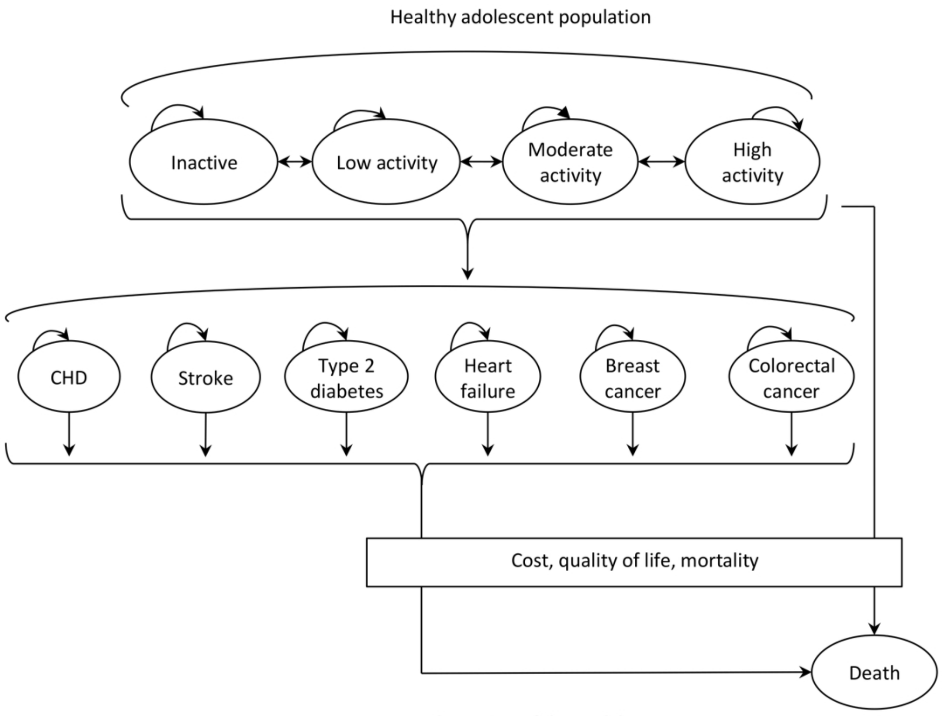

Conceptual overview of the model

168x125mm (300 x 300 DPI)

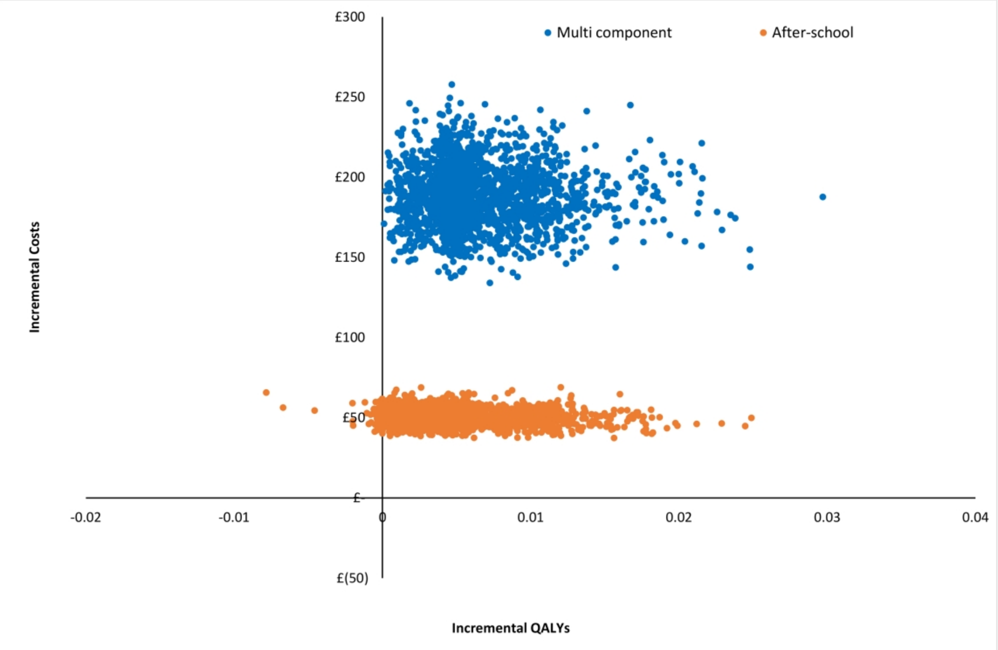

Scatter plot of incremental costs and QALYs for each intervention, relative to no intervention

259x168mm (300 x 300 DPI)

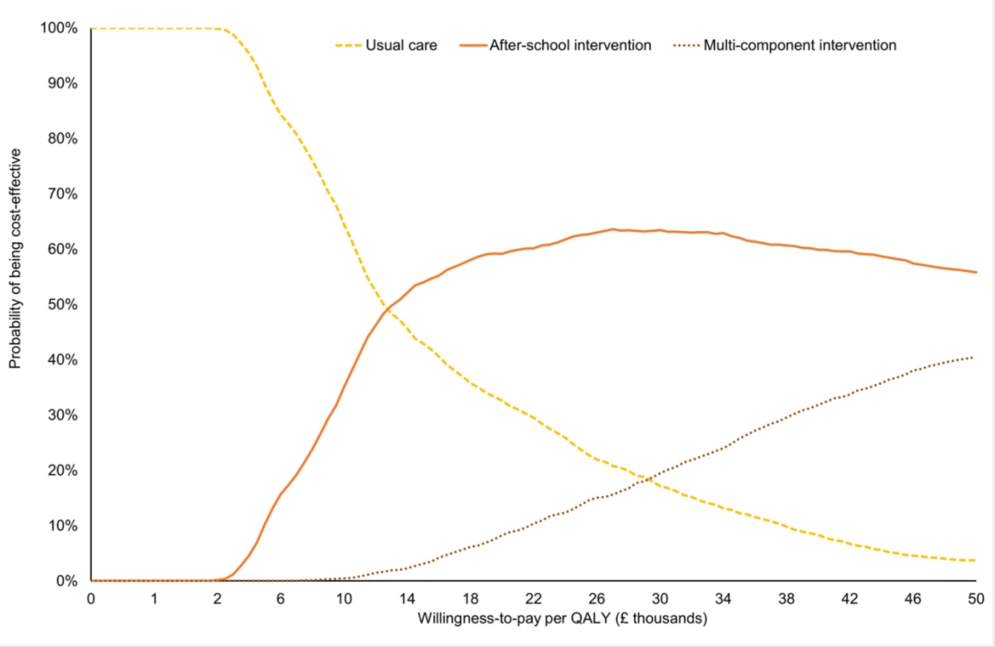

Cost-effectiveness acceptability curves

111x72mm (300 x 300 DPI)

Online Supplemental file

A. Model input parameters

Table S1: Physical activity at baseline and estimates of disease incidence

Parameters	Male				Female				Distribution	Source
	Inactive	Low	Moderate	High	Inactive	Low	Moderate	High		
Baseline PA levels	12%	7%	25%	56%	23%	35%	20%	22%	Fixed	[1]
Relative risk estimates used to adjust disease incidence										
CHD	1.0	0.87	0.78	0.70	1.0	0.87	0.78	0.70	lognormal	[2]
Stroke	1.0	0.85	0.81	0.76	1.0	0.85	0.81	0.76	lognormal	[2]
Heart failure	1.0	0.85	0.78	0.70	1.0	0.85	0.78	0.70	lognormal	[3]
Type 2 diabetes	1.0	0.93	0.75	0.60	1.0	0.93	0.75	0.60	lognormal	[4]
Breast cancer	-	-	-	-	1.0	0.97	0.94	0.86	lognormal	[5]
Colorectal cancer	1.0	0.90	0.83	0.79	1.0	0.90	0.83	0.79	lognormal	[5]
CHD incidence										[6]
<35	<0.01%	<0.01%	<0.01%	<0.01%	<0.01%	<0.01%	<0.01%	<0.01%	beta	
35-44	0.01%	0.00%	<0.01%	<0.01%	<0.01%	<0.01%	<0.01%	<0.01%	beta	
45-54	0.10%	0.09%	0.08%	0.07%	0.02%	0.01%	0.01%	0.01%	beta	
55-64	0.41%	0.36%	0.32%	0.29%	0.11%	0.10%	0.09%	0.08%	beta	
65-74	1.08%	0.94%	0.84%	0.76%	0.44%	0.38%	0.34%	0.31%	beta	
75+	1.67%	1.45%	1.30%	1.17%	0.67%	0.59%	0.53%	0.47%	beta	
Stroke incidence										[7]
<35	<0.01%	<0.01%	<0.01%	<0.01%	<0.01%	<0.01%	<0.01%	<0.01%	beta	
35-44	<0.01%	<0.01%	<0.01%	<0.01%	0.01%	0.01%	0.01%	0.01%	beta	
45-54	0.01%	0.01%	<0.01%	<0.01%	0.01%	0.01%	0.01%	0.01%	beta	
55-64	0.03%	0.03%	0.03%	0.02%	0.02%	0.02%	0.02%	0.02%	beta	
65-74	0.08%	0.07%	0.07%	0.06%	0.05%	0.05%	0.04%	0.04%	beta	
75-84	0.41%	0.35%	0.33%	0.31%	0.28%	0.23%	0.22%	0.21%	beta	
Heart failure incidence										[8]

For peer review only - http://bmjopen.bmj.com/site/about/guidelines.xhtml

Parameters	Male				Female				Distribution	Source
	Inactive	Low	Moderate	High	Inactive	Low	Moderate	High		
<35	<0.01%	<0.01%	<0.01%	<0.01%	<0.01%	<0.01%	<0.01%	<0.01%	beta	
35-44	0.03%	0.02%	0.02%	0.02%	0.03%	0.02%	0.02%	0.02%	beta	
45-54	0.04%	0.03%	0.03%	0.03%	0.01%	0.01%	0.01%	0.01%	beta	
55-64	0.21%	0.18%	0.16%	0.14%	0.08%	0.07%	0.07%	0.06%	beta	
65-74	0.48%	0.41%	0.37%	0.33%	0.28%	0.24%	0.22%	0.19%	beta	
75-84	1.14%	0.97%	0.89%	0.80%	0.64%	0.55%	0.50%	0.45%	beta	
Type 2 diabetes incidence										[9]
<20	0.01%	0.01%	0.01%	0.01%	0.03%	0.03%	0.03%	0.03%	beta	
20–29	0.05%	0.04%	0.04%	0.03%	0.14%	0.12%	0.11%	0.10%	beta	
30–39	0.17%	0.15%	0.13%	0.13%	0.24%	0.20%	0.18%	0.17%	beta	
40–49	0.51%	0.44%	0.38%	0.37%	0.38%	0.32%	0.28%	0.27%	beta	
50–59	0.99%	0.85%	0.74%	0.71%	0.67%	0.57%	0.50%	0.48%	beta	
60–69	1.43%	1.23%	1.07%	1.03%	1.02%	0.88%	0.76%	0.74%	beta	
70–79	1.53%	1.31%	1.15%	1.11%	1.23%	1.06%	0.92%	0.89%	beta	
80–89	1.05%	0.90%	0.79%	0.76%	0.87%	0.74%	0.65%	0.63%	beta	
Breast cancer incidence										[10]
<25					<0.01%	<0.01%	<0.01%	<0.01%	beta	
25 to 29					0.01%	0.01%	0.01%	0.01%	beta	
30 to 34					0.03%	0.03%	0.03%	0.02%	beta	
35 to 39					0.07%	0.07%	0.06%	0.05%	beta	
40 to 44					0.13%	0.13%	0.12%	0.10%	beta	
45 to 49					0.24%	0.23%	0.21%	0.19%	beta	
50 to 54					0.30%	0.29%	0.27%	0.23%	beta	
55 to 59					0.29%	0.28%	0.27%	0.23%	beta	
60 to 64					0.38%	0.36%	0.34%	0.29%	beta	
65 to 69					0.44%	0.42%	0.40%	0.34%	beta	
70 to 74					0.37%	0.35%	0.33%	0.29%	beta	
75 to 79					0.40%	0.39%	0.37%	0.32%	beta	
80 to 84					0.44%	0.42%	0.40%	0.34%	beta	
Colorectal cancer incidence										[11]
<30	<0.01%	<0.01%	<0.01%	<0.01%	<0.01%	<0.01%	<0.01%	<0.01%	beta	
30 to 34	0.01%	0.01%	0.01%	0.01%	0.01%	0.01%	0.01%	0.01%	beta	
35 to 39	0.01%	0.01%	0.01%	0.01%	0.01%	0.01%	0.01%	0.01%	beta	
40 to 44	0.01%	0.01%	0.01%	0.01%	0.01%	0.01%	0.01%	0.01%	beta	

For peer review only - http://bmjopen.bmj.com/site/about/guidelines.xhtml

Parameters	Male				Female				Distribution	Source
	Inactive	Low	Moderate	High	Inactive	Low	Moderate	High		
45 to 49	0.03%	0.03%	0.02%	0.02%	0.02%	0.02%	0.02%	0.02%	beta	
50 to 54	0.06%	0.05%	0.05%	0.05%	0.04%	0.04%	0.04%	0.03%	beta	
55 to 59	0.10%	0.09%	0.08%	0.08%	0.07%	0.06%	0.06%	0.06%	beta	
60 to 64	0.19%	0.17%	0.15%	0.15%	0.11%	0.10%	0.09%	0.09%	beta	
65 to 69	0.26%	0.23%	0.21%	0.20%	0.15%	0.13%	0.12%	0.12%	beta	
70 to 74	0.37%	0.34%	0.31%	0.29%	0.22%	0.20%	0.18%	0.17%	beta	
75 to 79	0.44%	0.40%	0.37%	0.35%	0.26%	0.24%	0.22%	0.21%	beta	
80 to 84	0.54%	0.49%	0.45%	0.43%	0.34%	0.31%	0.28%	0.27%	beta	

Inactive: <30; Low: 30-149; Moderate: 150-420 and High: ≥421 MVPA min per week

Table S2: Estimates of mortality parameters

Parameter	Value				Distribution	Source
	Male Mean	Male SE	Female mean	Female SE		
CHD case fatality rate						[6]
<54 years	0.129	0.013	0.1245	0.0125	beta	
55-64 years	0.132	0.013	0.1597	0.0160	beta	
65-74 years	0.177	0.018	0.2235	0.0224	beta	
75-84 years	0.244	0.024	0.3009	0.0301	beta	
85+ years	0.315	0.032	0.3668	0.0367	beta	
Stroke case fatality rate	0.1730	0.0152	0.1730	0.0152	beta	[12]
Heart failure case fatality rate						[13]
<35 years	0.0000	0.0000	0.0000	0.0000	beta	
35-55 years	0.0989	0.0050	0.0306	0.0001	beta	
55-64 years	0.1253	0.0037	0.1094	0.0001	beta	
65-74 years	0.1666	0.0028	0.1197	0.0001	beta	
75-84 years	0.1989	0.0027	0.1262	0.0001	beta	
85+ years	0.2279	0.0041	0.1240	0.0001	beta	
Death rate after 1st nonfatal CHD						[14]
<45 years	0.0130	0.0020	0.0130	0.0050	beta	
45-54 years	0.0170	0.0010	0.0260	0.0030	beta	
55-64 years	0.0380	0.0020	0.0350	0.0020	beta	

For peer review only - http://bmjopen.bmj.com/site/about/guidelines.xhtml

Parameter	Value				Distribution	Source
	Male Mean	Male SE	Female mean	Female SE		
65-74 years	0.0830	0.0020	0.0820	0.0030	beta	
75-84 years	0.1430	0.0040	0.1590	0.0040	beta	
85+ years	0.2620	0.0110	0.2660	0.0080	beta	
SMR for patients after a 1st nonfatal stroke	2.40	0.08	2.21	0.10	Lognormal	[15]
Death rate after 1st nonfatal heart failure						[13]
<55 years	0.0306	0.0001	0.0306	0.0001	beta	
55-64 years	0.1094	0.0001	0.1094	0.0001	beta	
65-74 years	0.1197	0.0001	0.1197	0.0001	beta	
75-84 years	0.1262	0.0001	0.1262	0.0001	beta	
85+ years	0.1240	0.0001	0.1240	0.0001	beta	
HR for death among patients with type 2 diabetes	1.80	0.0269	1.80	0.0269	lognormal	[16]
Breast cancer mortality	–	–	0.0290	0.0004	beta	[17]
Colorectal cancer mortality	0.1038	0.0006	0.1035	0.0006	beta	[17]
Lung cancer mortality	0.3523	0.0004	0.3112	0.0005	beta	[17]

HR = Hazard ratio; SMR = Standardised mortality ratio

For peer review only

For peer review only - http://bmjopen.bmj.com/site/about/guidelines.xhtml

B. Model validation results

1. Cohort profile

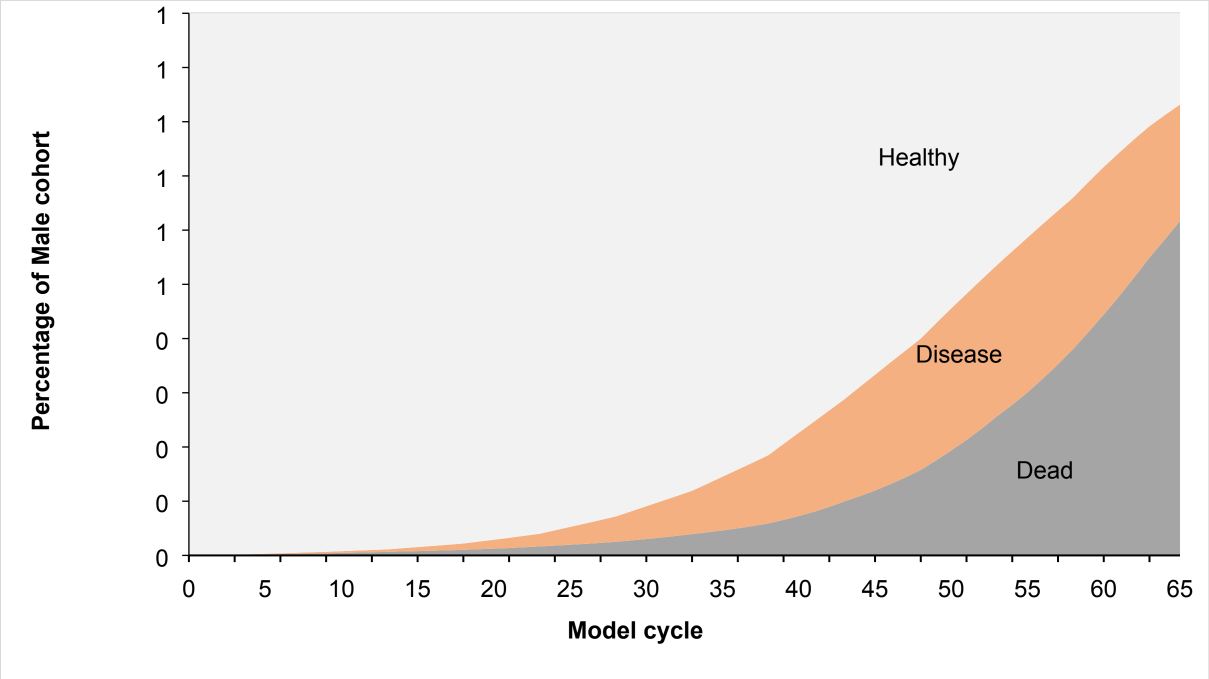

Figure S1: Cohort profile (male)

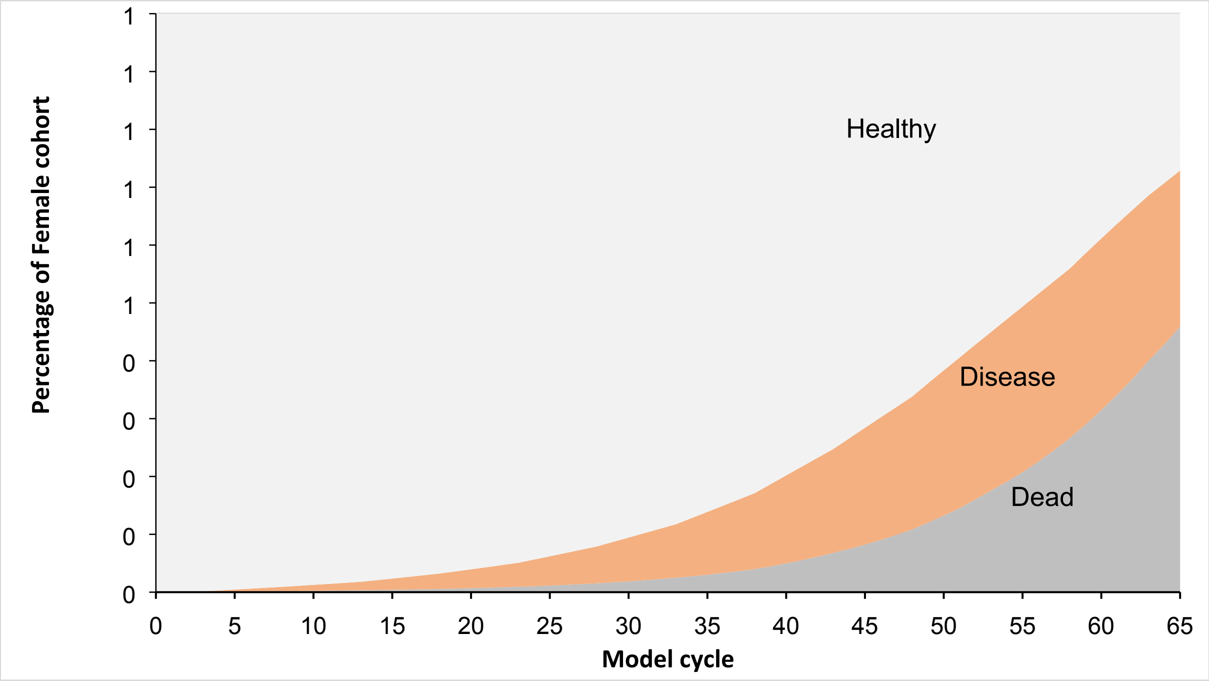

For peer review only - http://bmjopen.bmj.com/site/about/guidelines.xhtml

Figure S2: Cohort profile (female)

2. Prevalence of physical activity levels by age group and gender

Figure S3: Physical activity levels by age group in Male (HSE 2012 data vs no intervention 'control')

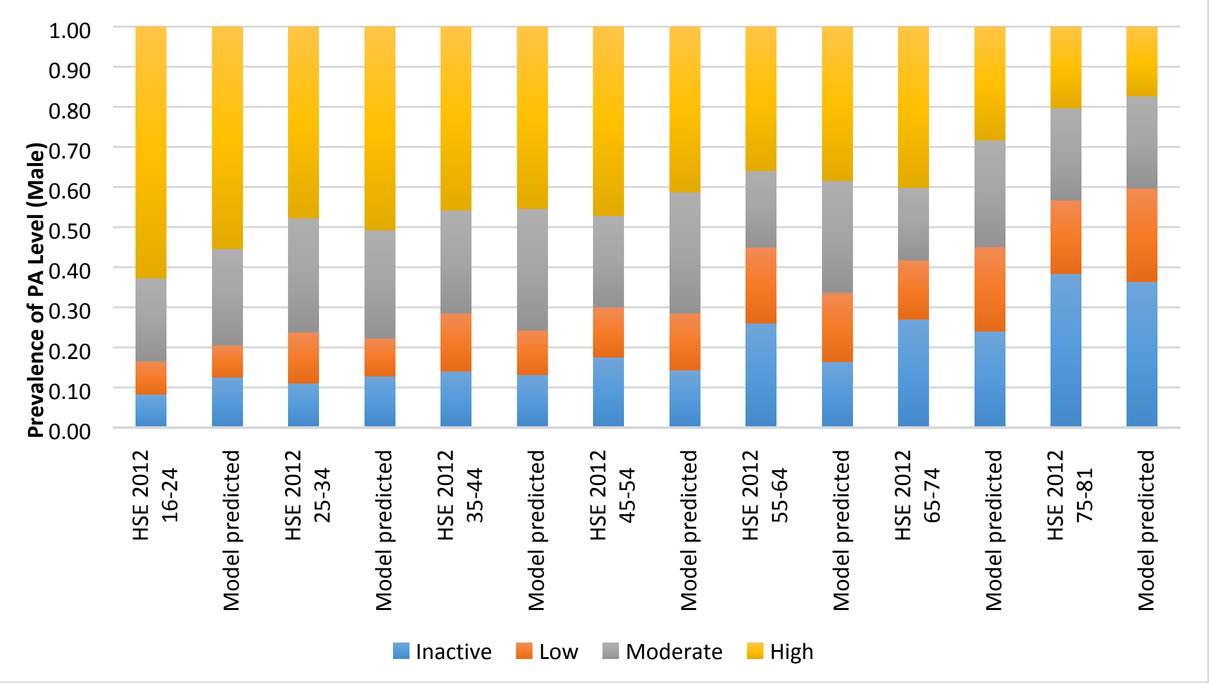

For peer review only - http://bmjopen.bmj.com/site/about/guidelines.xhtml

Figure S4: Physical activity levels by age group in Female (HSE 2012 data vs no intervention 'control')

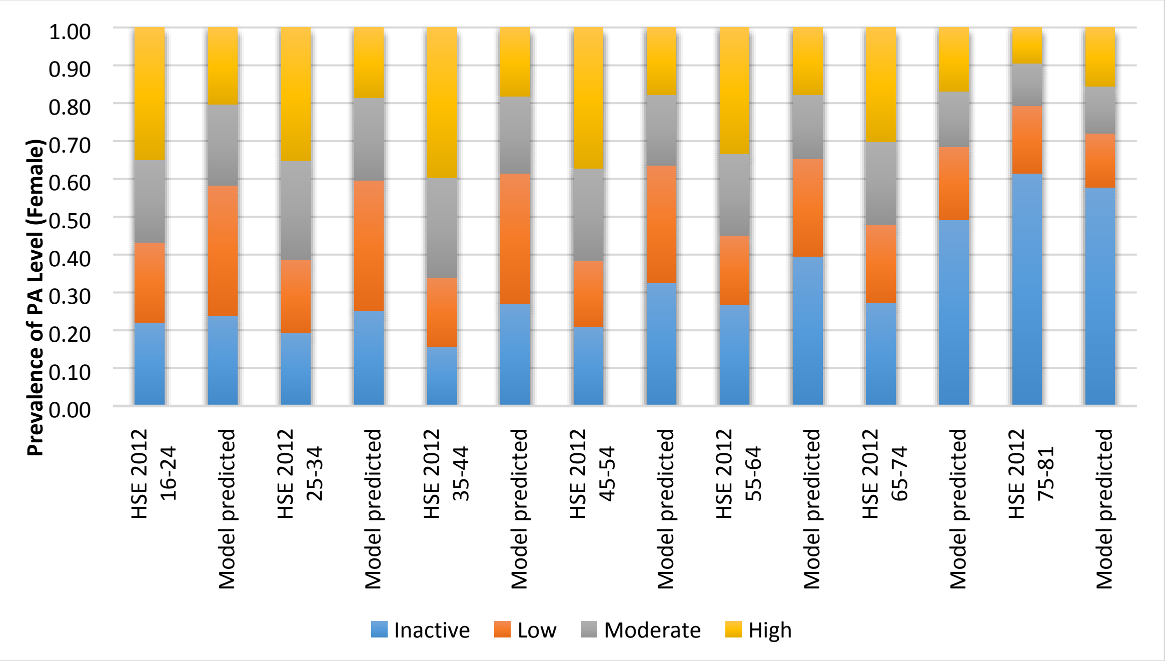

For peer review only - http://bmjopen.bmj.com/site/about/guidelines.xhtml

3. Risk of disease events

Figure S5: CHD incidence: model predicted versus data informing model parameter by age-group and gender

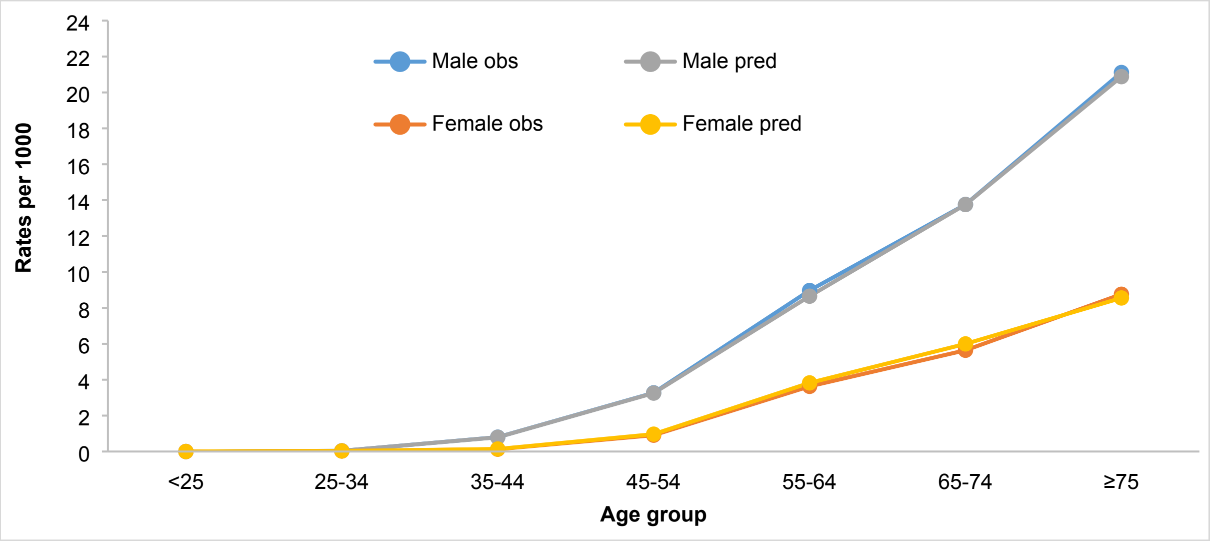

Figure S6: Stroke incidence: model predicted versus data informing model parameter by age-group and gender

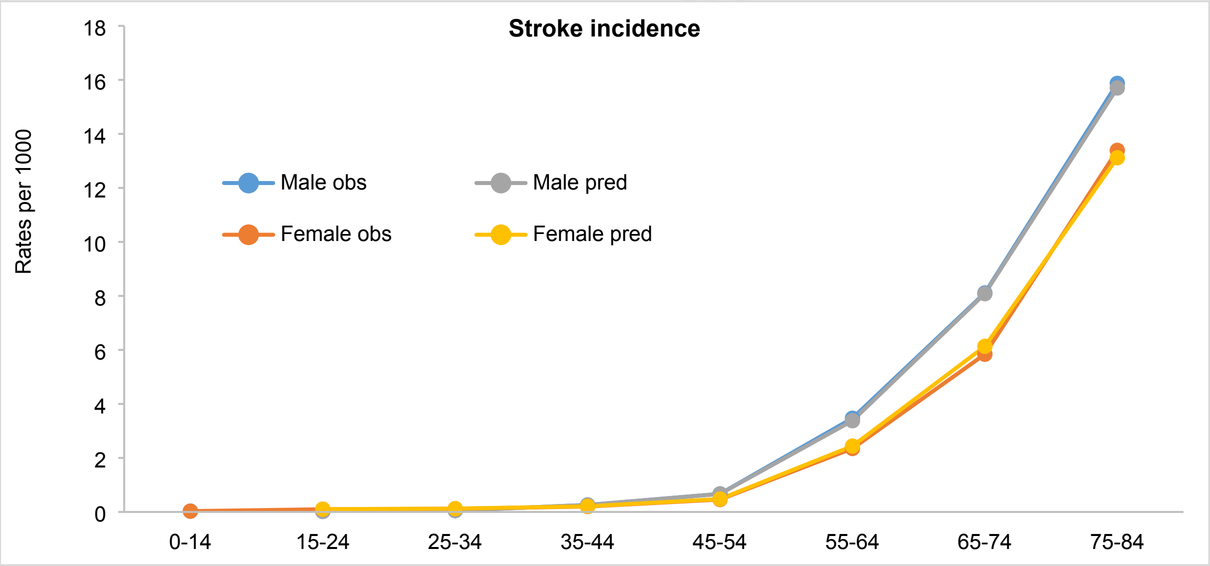

For peer review only - http://bmjopen.bmj.com/site/about/guidelines.xhtml

Figure S7: Heart failure incidence: model predicted versus data informing model parameter by age-group and gender

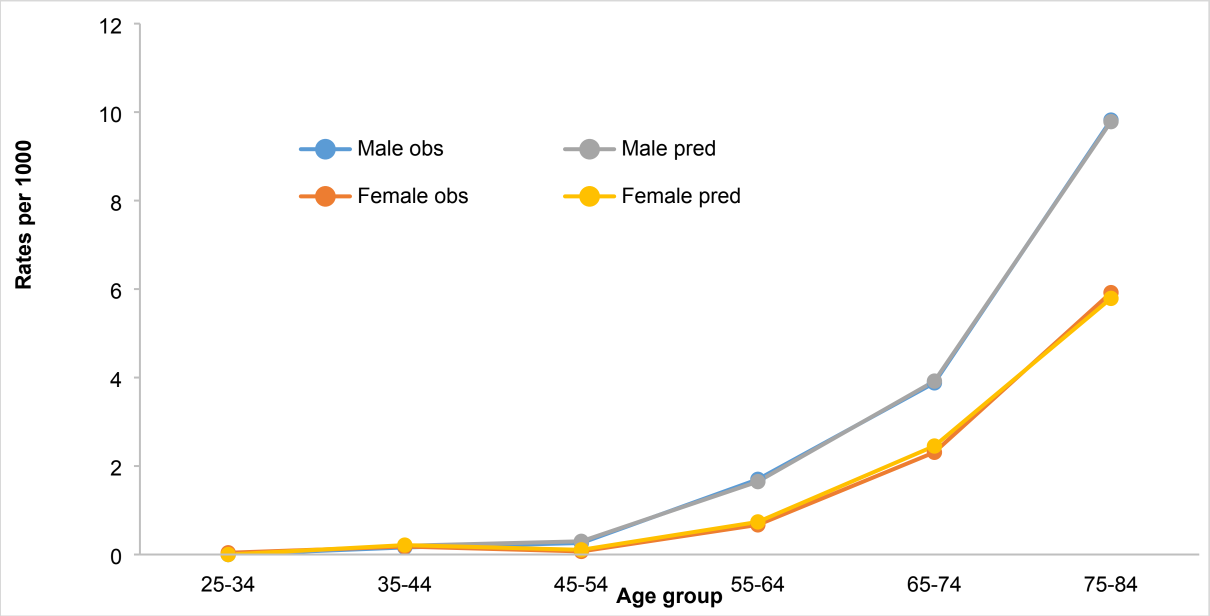

Figure S8: Incidence of type 2 diabetes: model predicted versus data informing model parameter by age-group and gender

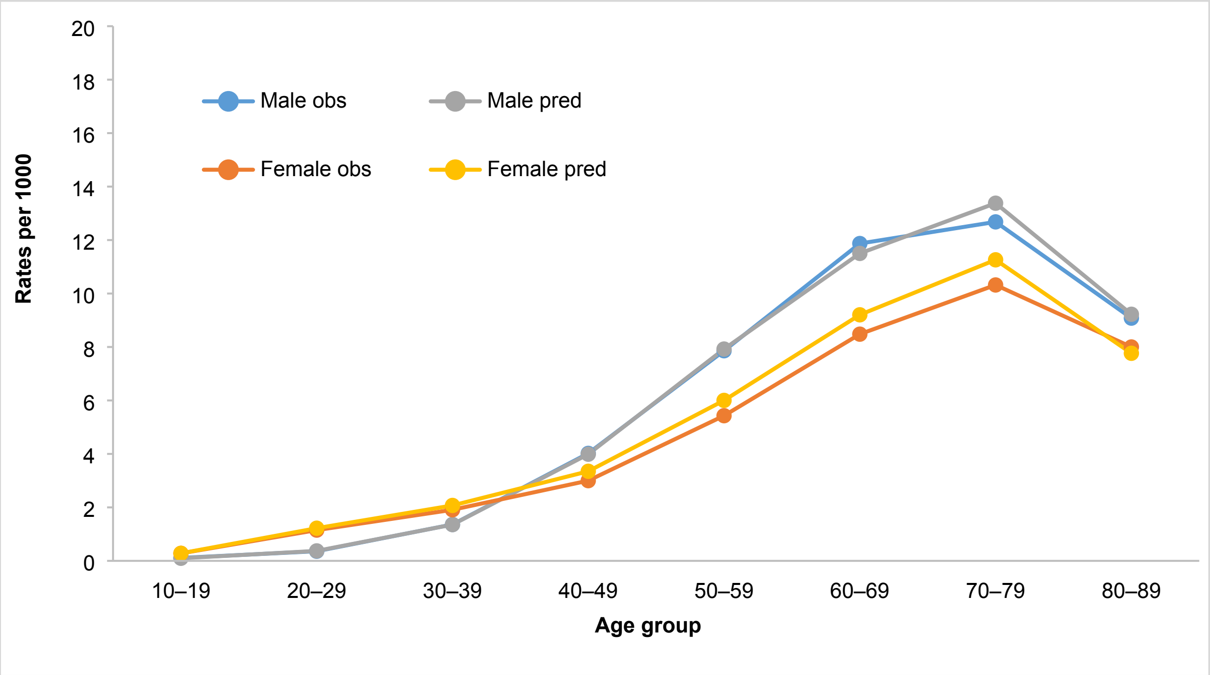

For peer review only - http://bmjopen.bmj.com/site/about/guidelines.xhtml

Figure S9: Breast cancer (female) incidence: model predicted versus data informing model parameter by age-group and gender

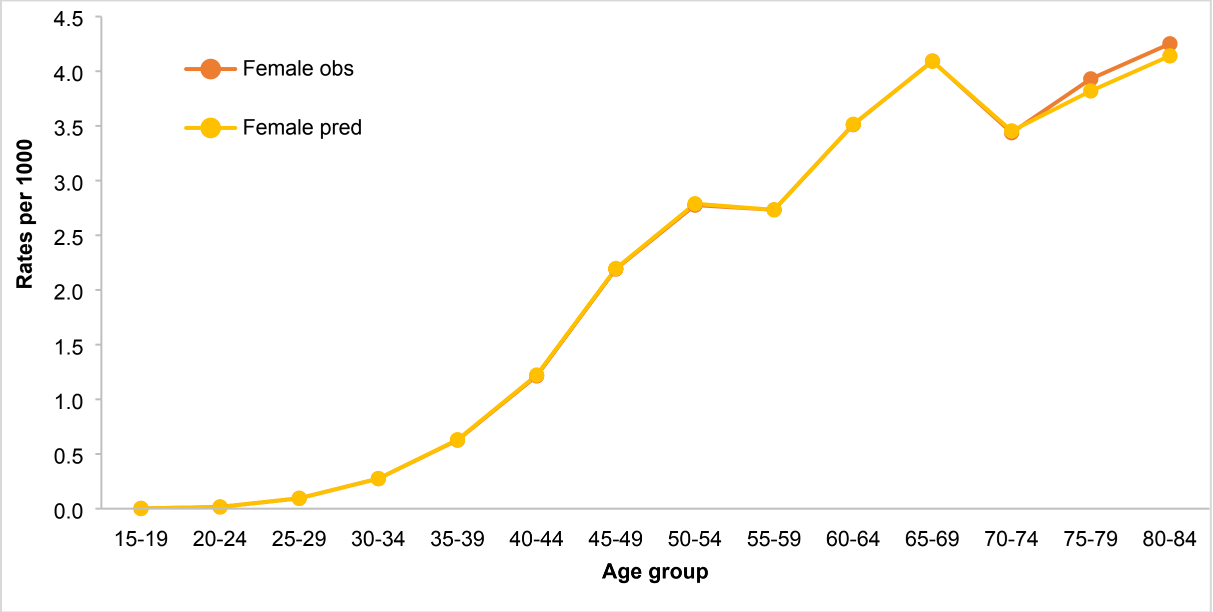

Figure S10: Colorectal cancer incidence: model predicted versus data informing model parameter by age-group and gender

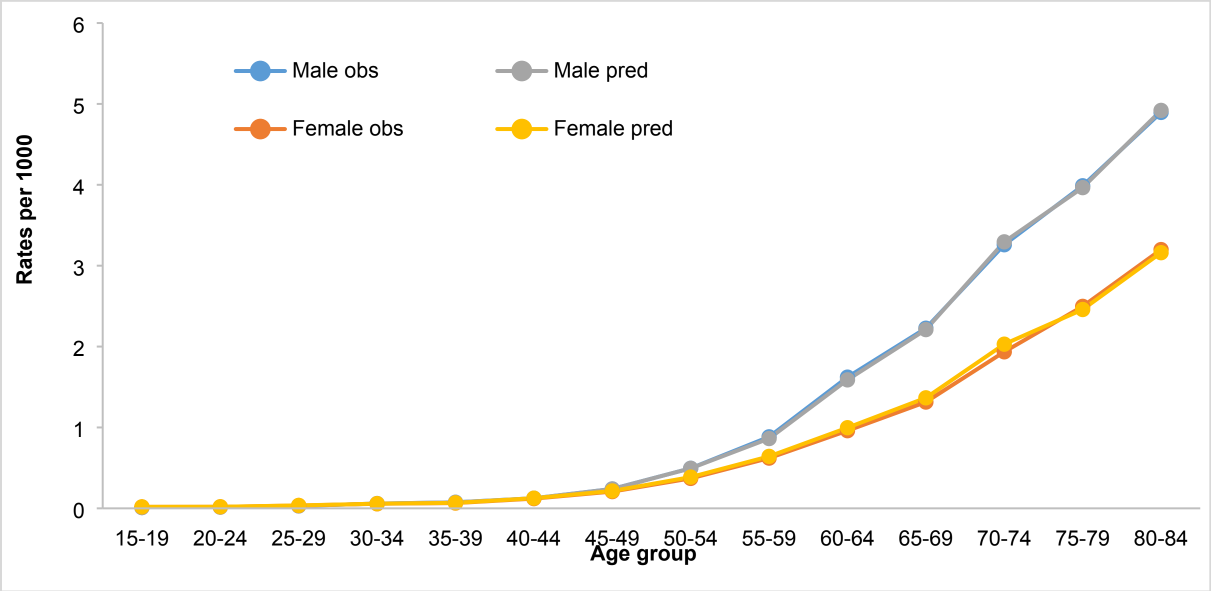

For peer review only - http://bmjopen.bmj.com/site/about/guidelines.xhtml

References

1. Scholes S, Mindell J (2013) *Physical activity in children*. In: Craig R, Mindell J, editors. Health Survey for England 2012.(2013) London: The Health and Social Care Information Centre

2. Wahid A, Manek N, Nichols M *et al.* Quantifying the Association Between Physical Activity and Cardiovascular Disease and Diabetes: A Systematic Review and Meta-Analysis. *J Am Heart Assoc* 2016; 5.

3. Pandey A, Garg S, Khunger M *et al.* Dose-Response Relationship Between Physical Activity and Risk of Heart Failure: A Meta-Analysis. *Circulation* 2015; 132: 1786-1794.

4. Smith AD, Crippa A, Woodcock J *et al.* Physical activity and incident type 2 diabetes mellitus: a systematic review and dose-response meta-analysis of prospective cohort studies. *Diabetologia* 2016.

5. Kyu HH, Bachman VF, Alexander LT *et al.* Physical activity and risk of breast cancer, colon cancer, diabetes, ischemic heart disease, and ischemic stroke events: systematic review and dose-response meta-analysis for the Global Burden of Disease Study 2013. *BMJ* 2016; 354: i3857.

6. Smolina K, Wright FL, Rayner M *et al.* Determinants of the decline in mortality from acute myocardial infarction in England between 2002 and 2010: linked national database study. *BMJ* 2012; 344: d8059.

7. Bamford J, Sandercock P, Dennis M *et al.* A prospective study of acute cerebrovascular disease in the community: the Oxfordshire Community Stroke Project 1981-86. 1. Methodology, demography and incident cases of first-ever stroke. *J Neurol Neurosurg Psychiatry* 1988; 51: 1373-1380.

8. Cowie MR, Wood DA, Coats AJ *et al.* Incidence and aetiology of heart failure; a population-based study. *Eur Heart J* 1999; 20: 421-428.

9. Sharma M, Nazareth I, Petersen I Trends in incidence, prevalence and prescribing in type 2 diabetes mellitus between 2000 and 2013 in primary care: a retrospective cohort study. *BMJ Open* 2016; 6: e010210.

10. Cancer Research UK. Breast cancer statistics. 2016. [accessed 26 October 2016.] Available from: http://www.cancerresearchuk.org/sites/default/files/cstream-node/cases_crude_f_breast_2.xls.

11. Cancer Research UK. Bowel cancer incidence. 2016. [accessed 26 October 2016.] Available from: http://www.cancerresearchuk.org/sites/default/files/cstream-node/cases_crude_bowel_2.xls.

12. Bamford J, Sandercock P, Dennis M *et al.* A prospective study of acute cerebrovascular disease in the community: the Oxfordshire Community Stroke Project--1981-86. 2. Incidence, case fatality rates and overall outcome at one year of cerebral infarction, primary intracerebral and subarachnoid haemorrhage. *J Neurol Neurosurg Psychiatry* 1990; 53: 16-22.

For peer review only

For peer review only - http://bmjopen.bmj.com/site/about/guidelines.xhtml

13. MacIntyre K, Capewell S, Stewart S *et al.* Evidence of improving prognosis in heart failure: trends in case fatality in 66 547 patients hospitalized between 1986 and 1995. *Circulation* 2000; 102: 1126-1131.

14. Turner D, Raftery J, Cooper K *et al.* The CHD challenge: comparing four cost-effectiveness models. *Value Health* 2011; 14: 53-60.

15. Bronnum-Hansen H, Davidsen M, Thorvaldsen P *et al.* Long-term survival and causes of death after stroke. *Stroke* 2001; 32: 2131-2136.

16. Seshasai SR, Kaptoge S, Thompson A *et al.* Diabetes mellitus, fasting glucose, and risk of cause-specific death. *N Engl J Med* 2011; 364: 829-841.

17. Office for National Statistics. Cancer survival in England - adults diagnosed. 2016. [accessed 26 October 2016.] Available from: http://www.ons.gov.uk/file?uri=/peoplepopulationandcommunity/healthandsocialcare/conditionsanddiseases/datasets/cancersurvivalratescancersurvivalinenglandadultsdiagnosed/20102014/finalreferencetablev2.xls.

For peer review only

For peer review only - http://bmjopen.bmj.com/site/about/guidelines.xhtml

CHEERS statement

Section/Item	Item no.	Recommendation	Reported on page no./line no.
**Title and abstract**			
Title	1	Identify the study as an economic evaluation, or use more specific terms such as ''cost-effectiveness analysis'' and describe the interventions compared.	1
Abstract	2	Provide a structured summary of objectives, perspective, setting, methods (including study design and inputs), results (including base-case and uncertainty analyses), and conclusions.	2
**Introduction**			
Background and objectives	3	Provide an explicit statement of the broader context for the study. Present the study question and its relevance for health policy or practice decisions.	4-5
**Methods**			
Target population and subgroups	4	Describe characteristics of the base-case population and subgroups analysed including why they were chosen.	5
Setting and location	5	State relevant aspects of the system(s) in which the decision(s) need(s) to be made	5
Study perspective	6	Describe the perspective of the study and relate this to the costs being evaluated.	13
Comparators	7	Describe the interventions or strategies being compared and state why they were chosen.	8
Time horizon	8	State the time horizon(s) over which costs and consequences are being evaluated and say why appropriate.	6
Discount rate	9	Report the choice of discount rate(s) used for costs and outcomes and say why appropriate	6
Choice of health outcomes	10	Describe what outcomes were used as the measure(s) of benefit in the evaluation and their relevance for the type of analysis performed	9
Measurement of effectiveness	11a	Single study–based estimates: Describe fully the design features of the single effectiveness study and why the single study was a sufficient source of clinical effectiveness data.	8
	11b	Synthesis-based estimates: Describe fully the methods used for the identification of included studies and synthesis of clinical effectiveness data.	8
Measurement and valuation of preference-based outcomes	12	If applicable, describe the population and methods used to elicit preferences for outcomes.	N/A
Estimating resources and costs	13a	Single study–based economic evaluation: Describe approaches used to estimate resource use associated with the alternative interventions. Describe primary or secondary research methods for valuing each resource item in terms of its unit cost. Describe any adjustments made to approximate to opportunity costs.	
	13b	Model-based economic evaluation: Describe approaches and data sources used to estimate resource use associated with model health states. Describe primary or secondary research methods for valuing each resource item in terms of its unit cost. Describe any adjustments made to approximate to opportunity costs.	9-10, Table 1
Currency, price date, and conversion	14	Report the dates of the estimated resource quantities and unit costs. Describe methods for adjusting estimated unit costs to the year of reported costs if necessary. Describe methods for converting costs into a common currency base and the exchange rate.	9

For peer review only - http://bmjopen.bmj.com/site/about/guidelines.xhtml

Section/Item	Item no.	Recommendation	Reported on page no./line no.
Choice of model	15	Describe and give reasons for the specific type of decision-analytic model used. Providing a figure to show model structure is strongly recommended.	5
Assumptions	16	Describe all structural or other assumptions underpinning the decision-analytic model.	5-8 & 11
Analytic methods	17	Describe all analytic methods supporting the evaluation. This could include methods for dealing with skewed, missing, or censored data; extrapolation methods; methods for pooling data; approaches to validate or make adjustments (e.g., half-cycle corrections) to a model; and methods for handling population heterogeneity and uncertainty.	5, 11-14
**Results**			
Study parameters	18	Report the values, ranges, references, and if used, probability distributions for all parameters. Report reasons or sources for distributions used to represent uncertainty where appropriate. Providing a table to show the input values is strongly recommended.	Table 1-2, Supplementary Table S1, S2
Incremental costs and outcomes	19	For each intervention, report mean values for the main categories of estimated costs and outcomes of interest, as well as mean differences between the comparator groups. If applicable, report incremental cost-effectiveness ratios.	14-15
Characterizing uncertainty	20a	Single study–based economic evaluation: Describe the effects of sampling uncertainty for estimated incremental cost, incremental effectiveness, and incremental cost-effectiveness, together with the impact of methodological assumptions (such as discount rate, study perspective).	N/A
	20b	Model-based economic evaluation: Describe the effects on the results of uncertainty for all input parameters, and uncertainty related to the structure of the model and assumptions.	15-16
Characterizing heterogeneity	21	If applicable, report differences in costs, outcomes, or cost-effectiveness that can be explained by variations between subgroups of patients with different baseline characteristics or other observed variability in effects that are not reducible by more information.	N/A
**Discussion**			
Study findings, limitations, generalizability, and current knowledge	22	Summarize key study findings and describe how they support the conclusions reached. Discuss limitations and the generalizability of the findings and how the findings fit with current knowledge.	16-18
**Other**			
Source of funding	23	Describe how the study was funded and the role of the funder in the identification, design, conduct, and reporting of the analysis. Describe other nonmonetary sources of support.	19
Conflicts of interest	24	Describe any potential for conflict of interest among study contributors in accordance with journal policy. In the absence of a journal policy, we recommend authors comply with International Committee of Medical Journal Editors' recommendations.	19

For consistency, the CHEERS statement checklist format is based on the format of the CONSORT statement checklist

For peer review only - http://bmjopen.bmj.com/site/about/guidelines.xhtml

BMJ Open

The cost-effectiveness of physical activity interventions in adolescents: model development and illustration using two exemplary interventions

Journal:	*BMJ Open*
Manuscript ID	bmjopen-2018-027566.R1
Article Type:	Research
Date Submitted by the Author:	24-Apr-2019
Complete List of Authors:	Gc, Vijay; University of York Centre for Health Economics, ; University of East Anglia Norwich Medical School,<br>Suhrcke, Marc; University of York Centre for Health Economics,<br>Atkin, Andrew; University of East Anglia Faculty of Medicine and Health Sciences,<br>van Sluijs, Esther; University of Cambridge, MRC Epidemiology Unit<br>Turner, David; University of East Anglia, Norwich Medical School
**Primary Subject Heading**:	Health economics
Secondary Subject Heading:	Public health, Sports and exercise medicine, Health services research
Keywords:	physical activity, young adult, HEALTH ECONOMICS, cost-effectiveness

SCHOLARONE™
Manuscripts

The cost-effectiveness of physical activity interventions in adolescents: model development and illustration using two exemplary interventions

Vijay S Gc[1,2], Marc Suhrcke[2], Andrew J Atkin[3,4], Esther van Sluijs[3], David Turner[1]

[1] Norwich Medical School, Faculty of Medicine and Health Sciences, University of East Anglia, Norwich, UK

[2] Centre for Health Economics, University of York, York, UK

[3] MRC Epidemiology Unit and UKCRC Centre for Diet and Activity Research (CEDAR), University of Cambridge School of Clinical Medicine, Cambridge, UK

[4] School of Health Sciences, Faculty of Medicine and Health Sciences, University of East Anglia, Norwich, UK

Corresponding author:

Mr Vijay S Gc, MPH

Centre for Health Economics

University of York

York, YO10 5DD

United Kingdom

Phone: +44 (0)1904 321973, Email: vijay.gc@york.ac.uk

Keywords: physical activity; adolescent; young adult; cost-effectiveness; economic model

Word count excluding title page, abstract, references, figures and tables: *3,617*

Number of tables and figures: Figures *3*, Tables *3*

For peer review only

For peer review only - http://bmjopen.bmj.com/site/about/guidelines.xhtml

Abstract

Objective: To develop a model to assess the long-term costs and health outcomes of physical activity interventions targeting adolescents.

Design: A Markov cohort simulation model was constructed with the intention of being capable of estimating long-term costs and health impacts of changes in activity levels during adolescence. The model parameters were informed by published literature and the analysis took a National Health Service perspective over a lifetime horizon. Univariate and probabilistic sensitivity analyses were undertaken.

Setting: School and community

Participants: A hypothetical cohort of adolescents aged 16 years at baseline.

Interventions: Two exemplar school-based: a comparatively simple, after-school intervention and a more complex multi-component intervention compared to usual care.

Primary and secondary outcome measures: Incremental cost-effectiveness ratio as measured by cost per quality-adjusted life year gained.

Results: The model gave plausible estimates of the long-term effect of changes in physical activity. The use of two exemplar interventions suggests that the model could potentially be used to evaluate a number of different physical activity interventions in adolescents. The key model driver was the degree to which intervention effects were maintained over time.

Conclusions: The model developed here has the potential to assess long-term value for money of physical activity interventions in adolescents. The two applications of the model indicate that complex interventions may not necessarily be the ones considered the most cost-effective when longer-term costs and consequences are taken into account.

Word count: 230 words

For peer review only - http://bmjopen.bmj.com/site/about/guidelines.xhtml

For peer review only

Article summary

Strengths and limitations of this study

- A Markov cohort model was developed based on currently available evidence to simulate the long-term impacts in terms of costs and quality-adjusted life years of physical activity interventions for adolescents.
- The study incorporates the most recent evidence on the effect of increased physical activity in long-term chronic disease conditions.
- The model builds on previously published cohort models and includes additional health states. In addition, extensive sensitivity analyses have been performed to reflect uncertainty in model structure and parameter assumptions.
- A limitation of the present study is that the change in activity level over time were estimated using population-level prevalence data due to unavailability of longitudinal data describing the lifetime trajectory of physical activity and exclusion of long-term impacts on other conditions, for example, mental health.

For peer review only

For peer review only - http://bmjopen.bmj.com/site/about/guidelines.xhtml

Introduction

Insufficient physical activity is a key risk factor for chronic diseases, such as cardiovascular disease (CVD), type 2 diabetes, and some types of cancer in the general population.[1,2] Physical activity in young people is associated with many health benefits including improved cardiovascular and mental health,[3,4] academic performance[5] and bone health.[6] Whilst physical activity typically declines with age, active children are more likely to become active adults.[7,8] Although the short- and long-term health benefits of physical activity are well-documented,[9,10] in England, nearly half of all young people fail to achieve the recommended levels of physical activity, based on self-reports.[11,12] When measured objectively using accelerometers, the prevalence of inactivity is higher still (91% boys and 98% girls).[13]

The high prevalence of physical inactivity in young people places a significant burden on health care services and the wider economy. A 2014 report estimated a lifetime cost of £53.3 billion related to inactivity among today's 11 to 25 year olds,[12] taking into consideration the fact that physical activity levels in childhood predict adult activity levels.[14] This estimate includes direct healthcare costs of treating the burden of type 2 diabetes, chronic heart disease, stroke and colon cancer, and the risk of premature death and morbidity associated with these illnesses.

In recent years, there has been increasing interest in identifying interventions to improve young people's activity levels. Although some school-based physical activity interventions show promising effects[15,16] the existing evidence is very limited in both quantity and quality.[17] While improvements in physical activity may have long-term health benefits, the evidence on the longer-term costs and health benefits of interventions in adolescence is particularly sparse. Trials generally do not have sufficient follow-up to capture associated longer-term costs and consequences directly.[18] Quantifying the economic and health benefits associated with physical activity interventions would help decision makers to make informed decisions, i.e., assessing whether these interventions are an efficient use of limited healthcare resources.

For peer review only - http://bmjopen.bmj.com/site/about/guidelines.xhtml

For peer review only

Furthermore, much of the health benefits of physical activity interventions occur in the future. Also, many interventions are focused on adult or elderly populations. The long-term costs and health benefits of physical activity interventions in adolescent population are a comparatively scarcely researched area. To fill this critical research gap, we developed a decision analytic model aimed at quantifying the potential long-term costs and quality-adjusted life-year (QALY) implications of changes in activity levels during adolescence. We then illustrate some of the practical implications of taking a longer-term perspective by applying the model to two exemplary intervention programmes to show how the changes in levels of adolescent physical activity could affect activity levels throughout lifetime, as well the resulting longer term costs and health benefits.

Methods

We developed a probabilistic, age- and gender-dependant state-transition Markov model to simulate a cohort of healthy adolescents. The model estimates the risk of cardiovascular, type 2 diabetes and oncological events over a lifetime, and associated costs and quality of life. The model structure was based on the previously published models[19,20] that assessed the cost-effectiveness of physical activity interventions in the adult population. The model combines information from a variety of sources relating to disease and physical activity epidemiology, mortality, effectiveness, health-related quality of life, and costs.

Structuring the model

Model and population type

A simulated cohort of 10,000 healthy adolescents aged 16 entered the model. The intervention is assumed to have been delivered at the start of the first model cycle. At the end of the first cycle, based on the intervention effectiveness evidence, a proportion of cohort members move to a higher activity level. Depending on the sustainability of the intervention effect, in subsequent years cohort

For peer review only - http://bmjopen.bmj.com/site/about/guidelines.xhtml

For peer review only

members obtain an annual probability of remaining at the new activity level or moving to a lower physical activity state or to a disease state or death.

Model states

The health states included in the model are 'healthy' (disease-free), 'having a chronic disease' and 'dead'. At the beginning of the simulation, we assumed that all cohort members start out as healthy, i.e. disease free. Within the model, physical activity is classified into four activity levels (inactive, low, moderate and high) based on weekly moderate-to-vigorous physical activity (MVPA). The model has 11 health states in total: four physical activity levels, six chronic disease conditions and death (Figure 1). Among the healthy, the risk of developing one of the six diseases depends on age, gender and activity level. For simplicity, we assumed that health states included in the model were mutually exclusive, and cohort members did not move between disease states.

The selection of the disease conditions was based on currently available evidence describing the association between physical activity and disease risk,[21-26] and economic and health burden of diseases related to physical inactivity in the UK.[27] Disease conditions are all associated with costs and impact upon quality of life. For each of the selected exemplar interventions, we re-ran the model changing appropriate data to reflect the costs and effectiveness of these interventions. Costs and QALYs were discounted at 3.5% as recommended for the National Institute for Health and Care Excellence (NICE) reference case.[28]

Time horizon

The time horizon of the model is 65 years, i.e. the model follows the cohort of 16 year olds until they reach 81 years – the average life expectancy in the UK.[29] A half-cycle correction was applied under the assumption that each transition happened halfway during the cycle.[30]

For peer review only - http://bmjopen.bmj.com/site/about/guidelines.xhtml

Populating the model

Baseline population and activity levels

Data on age and gender distribution of the initial population were obtained from the Office for National Statistics (ONS).[31] An estimate of baseline activity level (weekly MVPA) was taken from the 2012 Health Survey for England (HSE).[32] Participants were divided into four levels (<30, 30-149, 150-420 and ≥421 MVPA min per week) of activity by age and gender, based on the UK Department of Health's physical activity recommendations.[11] The moderate activity category equates to the current recommended level of physical activity.

Transition probabilities

To estimate the annual probability of developing each disease, the annual incidence rates for the disease conditions included in the model were taken from population-based studies in the UK.[33-39] These are probabilities for the general adult population and included all four activity levels. In order to adjust the differential risk of developing these disease conditions by activity level, we first derived the probability of developing that disease among inactive people, using the method presented by Hurley et al.[40] The probabilities for each condition among low, moderate and high activity levels in the cohort were estimated by multiplying the probabilities for the inactive population by corresponding relative risks (RRs) for low, moderate and high activity.[21-24,26] Supplementary section A Table S1 provides the transitional parameters.

Mortality rates

All-cause mortality rates by age and gender were derived from the ONS.[29] Mortality consists of disease-specific mortality and mortality due to other causes. We estimated other-cause mortality by subtracting the total number of deaths due to the six disease conditions included in the model from the all-cause mortality total. The other-cause mortality rates by age and gender were assumed constant in the sensitivity analysis. The model assumes that a given proportion of coronary heart disease (CHD), stroke and heart failure (HF) events would be immediately fatal and people who

For peer review only - http://bmjopen.bmj.com/site/about/guidelines.xhtml
For peer review only

survived one of these events had an increased subsequent risk of death (Supplementary section A Table S2). Case fatality rates for these health states were taken from published population-based studies in the UK.[41-43]

Individuals with type 2 diabetes were assigned an increased risk of mortality using data from a published meta-analysis.[44] Based on standardised mortality ratios reported in long-term follow-up studies of first-ever patients' stroke, a 2-fold increase in the risk of death after one year[45] was applied to the general mortality rates from the life tables to reflect the higher mortality burden post-vascular event. Annual mortality rates following the first HF event were estimated from ten-year case fatality rates in patients admitted with a principal diagnosis of HF in Scotland.[43] The age-adjusted five-year net survival rates from the ONS[46] were used to estimate an annual risk of cancer death. It was assumed that the mortality rates do not increase due to cancer beyond five years after a cancer diagnosis.

Interventions

We reviewed the literature to identify physical activity interventions targeting the adolescent population. For primarily illustrative purposes, we selected two interventions to test the model and explore the health and economic impact of smaller and greater changes in physical activity. The first was a more simple after-school intervention, not costly but likely with smaller benefits[47], the second a more complex, multi-component intervention – more costly intervention but with higher expected benefits[15]:

After school intervention programme. Mears and Jago[47] included six after-school interventions in their meta-analysis and reported the pooled mean difference of 4.84 (-0.94 to 10.61) min of MVPA per day. These programmes typically included structured or unstructured play, planned MVPA, single or multisport physical activity programme or adhering to specific instructions (e.g. maintaining sufficient intensity of exercise during a session).

For peer review only - http://bmjopen.bmj.com/site/about/guidelines.xhtml
For peer review only

Multi-component intervention. The intervention effect for school-based multi-component intervention was taken from a cluster-randomised trial[15] implemented in secondary schools in Australia. The intervention included multiple intervention strategies (e.g. active physical education lessons, enhanced school sport, supportive school physical activity policies) targeting physical activity. The reported difference in min of MVPA per day between the intervention and control arm at follow-up was 7.0 (2.7 to 11.4).

Costs, currency, price date and conversion

The annual costs incurred in each disease health state were based on previously published studies (Table 1). First-year costs and subsequent year costs are assigned for each of the health states modelled. Costs of CHD and stroke were taken from the statins health technology assessment (HTA).[48] Costs for HF were taken from the UKPDS study.[49] Costs for type 2 diabetes were based on Diabetes Glycaemic Education and Monitoring (DiGEM) trial and included medication and other healthcare costs.[50] Cost for breast cancer was taken from a screening appraisal for breast cancer.[51] The estimated cost is the weighted average treatment costs depending on the prognosis at diagnosis. Cost for colorectal cancer was based on a screening appraisal.[52] The appraisal reported the lifetime cost of colorectal cancer according to the cancer stage, and a weighted average cost was estimated using the proportion of cancers identified at each stage. All the costs were inflated to 2013-14.[53]

Health State Utility Values

Utility weights were used to value a year spent in each of the health states used in the model. A value of 1 means that the health state would be equivalent to full health and one year in that state would generate 1 QALY. For example, if an individual spent ten years in the CHD state with a utility of 0.65, they would accrue 6.5 QALYs. The same number of QALYs could be generated by spending 6.5 years in a health state of 1. The lower the utility value, the worse the health state is considered to be. The weights used to value disease health states are given in Table 1 and were taken from

For peer review only

For peer review only - http://bmjopen.bmj.com/site/about/guidelines.xhtml

Sullivan et al.[54], who used UK based community preferences to derive EQ-5D scores from the Medical Expenditure Panel Survey (MEPS). Utility weights for activity level by age- and gender were extracted from the HSE 2012 (Table 2).[55]

Table 1: Health state utilities and costs used in the model

Parameter	Value	Standard error	Distribution	Source
Health state utilities				[54]
CHD	0.65	0.0203	Beta (α=357, β=191)	
Stroke	0.52	0.0192	Beta (α=355, β=323)	
Heart failure	0.49	0.0194	Beta (α=326, β=335)	
Type 2 diabetes	0.66	0.0054	Beta (α=5032, β=2548)	
Breast cancer	0.76	0.0133	Beta (α=791, β=256)	
Colorectal cancer	0.67	0.0314	Beta (α=150, β=73)	
**Costs of health states**				
CHD 1st event	£5,562	£556	Gamma (α=100, β=56)	[48]
CHD subsequent	£214	£21	Gamma (α=100, β=2)	[48]
CHD fatal	£1,458	£146	Gamma (α=100, β=15)	[48]
Stroke 1st event	£10,062	£1,006	Gamma (α=100, β=101)	[48]
Stroke subsequent	£2,705	£270	Gamma (α=100, β=27)	[48]
Stroke fatal	£8,805	£881	Gamma (α=100, β=88)	[48]
Heart failure	£2,402	£240	Gamma (α=100, β=24)	[49]
HF subsequent	£815	£82	Gamma (α=100, β=8)	[49]
Type 2 diabetes	£1,257	£126	Gamma (α=100, β=13)	[50]
Breast cancer	£12,155	£1,215	Gamma (α=100, β=122)	[51]
Colorectal cancer	£16,978	£1,698	Gamma (α=100, β=170)	[52]

For peer review only

For peer review only - http://bmjopen.bmj.com/site/about/guidelines.xhtml

Table 2: Baseline utilities associated with physical activity level

Age-group	Utility values by activity level				Distribution	Source
	Inactive	Low	Moderate	High		
16-34	0.897	0.918	0.937	0.943	beta	[55]
35-44	0.770	0.889	0.914	0.927	beta	[55]
45-54	0.696	0.852	0.899	0.921	beta	[55]
55-64	0.648	0.861	0.863	0.907	beta	[55]
65-74	0.657	0.823	0.870	0.897	beta	[55]
65-74	0.701	0.829	0.850	0.876	beta	[55]

Inactive: <30; Low: 30-149; Moderate: 150-420 and High: ≥421 MVPA min per week

Modelling health benefits

We estimated the probability of moving to a higher activity level after intervention by adding intervention-specific MVPA minutes to baseline levels. We then calculated the proportion of cohort members that moved from one activity level to another, and we used this proportion as a transition probability. Members of the cohort who improved physical activity level at the end of cycle 0 were assumed to have a lower probability of developing CHD, stroke, HF or any of the cancers.

Estimation of the sustainability of intervention effect

The decline in activity levels over time in the 'no intervention'-group was modelled based on data from a recent meta-analysis examining the change in activity level from adolescence to adulthood[8] as well as using prevalence data from the 2012 HSE. The meta-analysis showed a decrease of 6.5 min/day of MVPA in boys and 5.5 min/day of MVPA in girls from adolescence to adulthood. As the review included studies reporting at least one measurement between both 13-19 years and 16-30 years, we assumed that the decrease in MVPA minutes was for a seven-year period. For the intervention group, a 50% decline in intervention effect per year post-intervention was assumed.

For peer review only

For peer review only - http://bmjopen.bmj.com/site/about/guidelines.xhtml

Therefore, the activity levels decreased towards the control activity level after seven years. The effect of this was that a number of individuals in the intervention group were re-categorised into higher activity groups immediately after the intervention. Over time, many of these individuals would fall back into lower activity groups, and at the end of seven-year, there was no real difference between intervention and control groups. To account for the decline in physical activity occurring with age across the life course, we estimated age-related activity levels using 2012 HSE data on physical activity prevalence by age and gender. Activity levels were estimated in three broad age groups: 24-44, 45-64 and ≥65 years to reflect activity-level differences in adulthood, middle-age and retirement.

Estimation of costs of intervention

Costs of delivering after-school intervention were taken from a cluster randomised feasibility study in the UK.[56] The authors reported cost estimates (£49 per participant, 2012-13 price) of a teaching assistant led extra-curricular physical activity intervention. We took that as an indicative cost of the after-school intervention, inflated to 2014 prices (£51 per participant). The cost includes intervention delivery costs, one-off training and non-recurrent costs such as consultation and intervention development work.

Sutherland et al. performed an economic evaluation alongside the multi-component intervention [57] and reported that the intervention costed AU$ 394 per participant. This cost includes opportunity costs for delivery of strategies by school staff and community sport and fitness providers, materials and printing. We converted this cost to 2014 pound sterling (£190 per participant) by applying the gross domestic product deflator index (GDP values) and purchasing power parities conversion rates using the CCEMG-EPPI-Centre Cost Converter (V1.5).[58] We added the intervention cost in the first year for the intervention groups.

For peer review only

For peer review only - http://bmjopen.bmj.com/site/about/guidelines.xhtml

Validation

The model structure, data sources and the effectiveness evidence used in the exemplar interventions and model results were validated by the study team comprising health economists, behavioural epidemiologists and trialists. Internal validity of the model code was ensured using several tests and by assuming a constant total population throughout the calculations. Furthermore, model predictions were examined to make sure that results from the model were consistent with the model's specifications. We specifically checked lifetime incidence and mortality, as well as physical activity prevalence by age and gender. Details can be found in the Supplementary material, section B Figures S1-S10.

Cost-effectiveness analysis

We used the NICE reference case[28] and followed existing guidelines for modelling.[59] The analysis was performed from the perspective of the NHS and personal social services. Costs and health outcomes were discounted at 3.5% per year.[28] We estimated the cost-effectiveness ratio for each intervention compared to 'no intervention'. The incremental costs and QALYs gained by the intervention were estimated and averaged across the simulated cohort. The incremental cost-effectiveness ratio (ICER) was estimated as a ratio between the additional expected cost of the intervention, and the additional expected QALYs gained, both relative to the 'no intervention' alternative. The intervention was considered cost-effective if the ICER was no more than the lower NICE recommended threshold of £20,000 per QALY. The uncertainty surrounding the estimates of cost-effectiveness is presented using cost-effectiveness acceptability curves (CEACs). A CEAC shows the probability that an intervention is cost-effective compared with alternative intervention for a range of cost-effectiveness thresholds.[60]

For peer review only - http://bmjopen.bmj.com/site/about/guidelines.xhtml

Sensitivity analyses

We performed sensitivity analyses by changing intervention decay rates and time horizon that affect cost-effectiveness results. Deterministic one-way, scenario and extreme value analyses were undertaken. This was complemented by probabilistic sensitivity analysis (PSA) to assess the combined effects of uncertainty in the input parameters by simultaneously sampling input parameter values from within a specified distribution using Monte Carlo simulations (2,000 iterations). Uncertainty about the sustainability of the intervention effects was assessed by varying the decay rates between 0% and 100%. In our base-case analysis, we assumed that the intervention effects are sustained for the first year but decay exponentially at a rate of 50% per annum thereafter, resulting in virtually no intervention effect after five years.

Probabilities of disease events and utilities were assumed to follow a beta distribution; costs followed a gamma distribution and risk reductions/hazard ratios a lognormal distribution.[61] The model was developed and implemented in Microsoft Excel.

Patient and public involvement

Public involvement informed the questions addressed in the overarching research project of which this study is a part. No further public involvement was sought with regards to the development of the research question, the outcome measures or the study design.

Results

Base-case analysis

In our base-case analysis, both after-school and multi-component interventions were associated with higher costs and were more effective than no intervention (

Table 3). The multi-component intervention was associated with a QALY gain of 0.002 at an incremental cost of £138, compared to the after-school intervention, yielding an ICER of £68,056.

For peer review only - http://bmjopen.bmj.com/site/about/guidelines.xhtml

Table 3: Incremental cost-effectiveness ratios in the base-case and sensitivity analyses

	Total cost (£)	Total QALYs	Incremental cost (£)	Incremental QALY	ICER
**Base-case analysis**					
No intervention	4,441	21.705	–	–	–
After-school intervention	4,491	21.710	50.64	0.004	11,486
Multi-component intervention	4,629	21.712	137.89	0.002	68,056
**No (0%) decay of intervention effects**					
No intervention	4,437	21.707	–	–	–
After-school intervention	4,451	21.898	14.32	0.191	75
Multi-component intervention	4,571	21.987	119.59	0.089	1,342
**33% decay of intervention effects**					
No intervention	4,430	21.705	–	–	–
After-school intervention	4,479	21.718	48.86	0.013	3,661
Multi-component intervention	4,616	21.726	136.94	0.008	17,661
**100% decay of intervention effects**					
No intervention	4,432	21.705	–	–	–
After-school intervention	4,484	21.707	51.42	0.002	28,838
Multi-component intervention	4,621	21.708	137.61	0.001	189,897
**Time horizon (10 years)**					
No intervention	41	7.167	–	–	–
After-school intervention	92	7.170	51.47	0.004	14,204
Multi-component intervention	231	7.172	138.60	0.002	75,100
**Time horizon (20 years)**					
No intervention	219	12.835	–	–	–
After-school intervention	270	12.838	51.09	0.004	13,414
Multi-component intervention	408	12.840	138.19	0.002	72,346

ICER, incremental cost-effectiveness ratio (incremental cost/incremental QALY); QALY, quality-adjusted life year.

For peer review only

Sensitivity analyses

Sensitivity analyses were implemented, varying the base-case assumptions and inputs, as outlined in the methods section (Table 3). Both the after-school and multi-component interventions had more favourable ICERs at lower decay rates, indicating that cost-effectiveness of physical activity interventions depends on the sustainability of intervention effects over time. The results of PSA are presented on the cost-effectiveness plane (Figure 2), with simulations found to lie predominantly in the northeast quadrant indicating – as expected – an improved health outcome but also at higher spending on physical activity interventions.

Figure 3 depicts the probability of the interventions being cost-effective. At a threshold value of £20,000 per QALY gained, the after-school intervention has the highest probability of being cost-effective (59%) while for the multi-component intervention this probability is at 8%.

Discussion

Main findings

We found that modelling the long-term effects of physical activity among adolescent is feasible and that early interventions probably make only small differences to lifetime costs and QALYs. Although more complex and costly interventions may have bigger health gains, that need not mean they are automatically better valued for money in the longer-term. Our results underline that cost-effectiveness estimates are critically sensitive to assumptions around the sustainability of intervention effects. Our base-case analysis assumed that intervention effects decay exponentially at a rate of 50% per year post-intervention, such that intervention group participants revert towards the control activity levels at the end of a seven-year period.

Comparison with previous models

Two previous UK-based modelling studies evaluating the cost-effectiveness of community based physical activity interventions included young people. Although Frew et al[20] used the same basic

For peer review only - http://bmjopen.bmj.com/site/about/guidelines.xhtml

modelling approach, their model included only three activity categories and study participants included both young people and adults (16-70 year olds). By contrast, our model has four physical activity categories, included two more health states and health effects are modelled over a lifetime (with a time horizon of 65 years). Pringle et al[62] evaluated seven broad categories of community based physical activity interventions (one of which was related to the interventions considered here). Their analysis was based on the NICE/Matrix model[63] which used two activity categories (active or inactive) with 4 disease states. However, they did not focus on young people. Recently, Lee and colleagues[64] modelled the economic and health impact of increasing children's physical activity in the US. However, unlike in the current model, their model specifically looked at the influence of physical activity on weight status and metabolic profiles, and ignored decay in intervention effects or the naturally occurring decline in physical activity associated with ageing.

Strengths and limitations

Although our model used a similar modelling framework as previous models,[19,20] we included additional health states and focused on adolescent physical activity interventions – this approach has hitherto been neglected, despite the potential importance of intervening at this key stage. We also include the most up to date available evidence on disease conditions. UK-specific incidence rates were used to ensure that patients entering the model match the likely distribution of events in the UK. We chose not to include sedentary behaviour, as there is ongoing debate around its impact on health independent of physical activity.[65]

As with all models, assumptions were required for the analysis. The model presented here is a simplification of a very complex problem. The baseline age of the cohort is 16 years and the effect of physical activity interventions are likely to differ depending on the population age at baseline. We included six disease conditions that have established links with physical (in)activity. This might underestimate the potential impact of physical activity on other disease conditions, most notably mental health. The effect of physical activity on the prevention of depression is still a subject of

For peer review only

For peer review only - http://bmjopen.bmj.com/site/about/guidelines.xhtml

debate[66], and a clear dose-response relationship between physical activity and reduced depression is not readily apparent.[67] Further empirical evidence is required to facilitate its inclusion in a future iteration of the model. The current model does not allow for transitions between disease states as this requires more complex modelling. However, this may underestimate the potential impact of physical activity. For example, participants with type 2 diabetes tend to have a higher risk of developing cardiovascular conditions.[68] Although the intervention was aimed at adolescents, due to the nature of the disease conditions included in the model, it would mainly be older adults who develop these diseases.

Our assumption on the decay rates of the additional effect of the intervention, which was based on previous modelling studies,[19,69,70] would mean that there would be very little difference in activity between groups at time points when individuals are starting to develop these diseases. We tested different assumptions on the maintenance of intervention effect to examine influence in cost-effectiveness results. Further research into maintenance of intervention effect would provide valuable information. Our analysis focused on physical activity and only considered direct effects that might result from changes in this health behaviour, while holding any other health behaviours constant. In the real world, physical activity would be expected to interact with other health behaviour choices, in ways that might well affect longer term cost and health outcomes. The existing, very sparse, literature on the interaction between different health behaviours suggest a complex and likely context-specific picture.[64,71]

For peer review only

Conclusion

Interventions to promote physical activity among adolescents represent a potentially promising public health measure to reduce the burden of cardiovascular and other non-communicable diseases. Faced with limited resources, governments need to carefully weigh the costs of any proposed interventions against the associated health benefits expected to be realised over the longer term, in order to ensure that net health gains are maximised. The model developed here has

For peer review only - http://bmjopen.bmj.com/site/about/guidelines.xhtml

the potential to assess the long-term, beyond trial duration, value-for-money of such interventions. The two purely illustrative applications of the model convey the notion that complex, resource intensive interventions may not necessarily be the ones considered the better buys compared to cheaper, more targeted ones. Maintaining the effect of any behaviour change interventions is challenging as they require personal commitment, encouragement and support over time.

Author contributions

VG designed the model, performed analysis and wrote the first draft of the manuscript. DT and MS supervised the process and provided input into the interpretation of results. AA and EvS provided critical comments on model structure and data analysis. All authors contributed to the critical revision of the manuscript and approved the final version of the manuscript.

Funding

This report is an independent research commissioned and funded by the Department of Health Policy Research Programme (opportunities within the school environment to shift the distribution of activity intensity in adolescents, PR-R5-0213-25001). The views expressed in this publication are those of the author(s) and not necessarily those of the Department of Health. This work was also supported by the Medical Research Council (unit programme number: MC_UU_12015/7). The work was undertaken under the auspices of the Centre for Diet and Activity Research (CEDAR), a UKCRC Public Health Research Centre of Excellence which is funded by the British Heart Foundation, Cancer Research UK, Economic and Social Research Council, Medical Research Council, the National Institute for Health Research, and the Wellcome Trust (MR/K023187/1).

Availability of data and material

The model was developed using data from publicly available sources, and all the model inputs are described in the paper.

For peer review only - http://bmjopen.bmj.com/site/about/guidelines.xhtml

Competing interests statement

Nothing to report.

Ethics approval and consent to participate

Not applicable.

Acknowledgements

We thank Dr Katie Morton and Dr Kirsten Corder for their comments in the early stage of model development work.

For peer review only

For peer review only - http://bmjopen.bmj.com/site/about/guidelines.xhtml

References

1. Reiner M, Niermann C, Jekauc D *et al.* Long-term health benefits of physical activity--a systematic review of longitudinal studies. *BMC Public Health* 2013; 13: 813.

2. Lee IM, Shiroma EJ, Lobelo F *et al.* Effect of physical inactivity on major non-communicable diseases worldwide: an analysis of burden of disease and life expectancy. *Lancet* 2012; 380: 219-229.

3. Ekelund U, Luan J, Sherar LB *et al.* Moderate to vigorous physical activity and sedentary time and cardiometabolic risk factors in children and adolescents. *JAMA* 2012; 307: 704-712.

4. Biddle SJ, Asare M Physical activity and mental health in children and adolescents: a review of reviews. *Br J Sports Med* 2011; 45: 886-895.

5. Singh A, Uijtdewilligen L, Twisk JW *et al.* Physical activity and performance at school: a systematic review of the literature including a methodological quality assessment. *Arch Pediatr Adolesc Med* 2012; 166: 49-55.

6. Hallal PC, Victora CG, Azevedo MR *et al.* Adolescent Physical Activity and Health. *Sports Medicine* 2006; 36: 1019-1030.

7. Telama R, Yang X, Viikari J *et al.* Physical activity from childhood to adulthood: a 21-year tracking study. *Am J Prev Med* 2005; 28: 267-273.

8. Corder K, Winpenny E, Love R *et al.* Change in physical activity from adolescence to early adulthood: a systematic review and meta-analysis of longitudinal cohort studies. *Br J Sports Med* 2017; doi:10.1136/bjsports-2016-097330.

9. Department of Health. *UK physical activity guidelines*. London: Department of Health, 2011.

10. Warburton DE, Nicol CW, Bredin SS Health benefits of physical activity: the evidence. *CMAJ* 2006; 174: 801-809.

11. Department of Health. *Start active, stay active: a report on physical activity from the four home countries' Chief Medical Officers*. Department of Health, 2011.

For peer review only - http://bmjopen.bmj.com/site/about/guidelines.xhtml

For peer review only

12. Evans K. The inactivity time bomb. The economic cost of physical inactivity in young people: a StreetGames/Cebr report. 2014. [accessed 13 January 2017.] Available from: http://www.streetgames.org/sites/default/files/The-Inactivity-TimeBomb-StreetGames-Cebr-report-April-2014.pdf.

13. Cooper AR, Goodman A, Page AS *et al.* Objectively measured physical activity and sedentary time in youth: the International children's accelerometry database (ICAD). *Int J Behav Nutr Phys Act* 2015; 12: 113.

14. Telama R Tracking of physical activity from childhood to adulthood: a review. *Obes Facts* 2009; 2: 187-195.

15. Sutherland RL, Campbell EM, Lubans DR *et al.* The Physical Activity 4 Everyone Cluster Randomized Trial: 2-Year Outcomes of a School Physical Activity Intervention Among Adolescents. *Am J Prev Med* 2016; 51: 195-205.

16. Grydeland M, Bergh IH, Bjelland M *et al.* Intervention effects on physical activity: the HEIA study - a cluster randomized controlled trial. *Int J Behav Nutr Phys Act* 2013; 10: 17.

17. Borde R, Smith JJ, Sutherland R *et al.* Methodological considerations and impact of school-based interventions on objectively measured physical activity in adolescents: a systematic review and meta-analysis. *Obes Rev* 2017; 18: 476-490.

18. Buxton MJ, Drummond MF, Van Hout BA *et al.* Modelling in economic evaluation: an unavoidable fact of life. *Health Econ* 1997; 6: 217-227.

19. Roux L, Pratt M, Tengs TO *et al.* Cost effectiveness of community-based physical activity interventions. *Am J Prev Med* 2008; 35: 578-588.

20. Frew EJ, Bhatti M, Win K *et al.* Cost-effectiveness of a community-based physical activity programme for adults (Be Active) in the UK: an economic analysis within a natural experiment. *Br J Sports Med* 2014; 48: 207-212.

For peer review only

For peer review only - http://bmjopen.bmj.com/site/about/guidelines.xhtml

21. Wahid A, Manek N, Nichols M *et al.* Quantifying the Association Between Physical Activity and Cardiovascular Disease and Diabetes: A Systematic Review and Meta-Analysis. *J Am Heart Assoc* 2016; 5.

22. Pandey A, Garg S, Khunger M *et al.* Dose-Response Relationship Between Physical Activity and Risk of Heart Failure: A Meta-Analysis. *Circulation* 2015; 132: 1786-1794.

23. Smith AD, Crippa A, Woodcock J *et al.* Physical activity and incident type 2 diabetes mellitus: a systematic review and dose-response meta-analysis of prospective cohort studies. *Diabetologia* 2016.

24. Kyu HH, Bachman VF, Alexander LT *et al.* Physical activity and risk of breast cancer, colon cancer, diabetes, ischemic heart disease, and ischemic stroke events: systematic review and dose-response meta-analysis for the Global Burden of Disease Study 2013. *BMJ* 2016; 354: i3857.

25. Brenner DR, Yannitsos DH, Farris MS *et al.* Leisure-time physical activity and lung cancer risk: A systematic review and meta-analysis. *Lung Cancer* 2016; 95: 17-27.

26. Tardon A, Lee WJ, Delgado-Rodriguez M *et al.* Leisure-time physical activity and lung cancer: a meta-analysis. *Cancer Causes Control* 2005; 16: 389-397.

27. Allender S, Foster C, Scarborough P *et al.* The burden of physical activity-related ill health in the UK. *J Epidemiol Community Health* 2007; 61: 344-348.

28. National Institute for Health and Care Excellence. *Guide to the methods of technology appraisal 2013*. London: National Institute for Health and Care Excellence, 2013.

29. Office for National Statistics. National Life Tables: United Kingdom. 2016. [accessed 3 November 2016.] Available from: http://www.ons.gov.uk/file?uri=/peoplepopulationandcommunity/birthsdeathsandmarriages/lifeexpectancies/datasets/nationallifetablesenglandreferencetables/current/nlteng1315reg.xls.

30. Naimark DM, Bott M, Krahn M The half-cycle correction explained: two alternative pedagogical approaches. *Med Decis Making* 2008; 28: 706-712.

For peer review only

For peer review only - http://bmjopen.bmj.com/site/about/guidelines.xhtml

31. Office for National Statistics. MYE1: Population Estimates Summary for the UK, mid-2014. 2016. [accessed 20 October 2016.] Available from: https://www.ons.gov.uk/file?uri=/peoplepopulationandcommunity/populationandmigration/populationestimates/datasets/populationestimatesanalysistool/mid2014/analysistoolmid2014uk.zip.

32. Scholes S, Mindell J (2013) *Physical activity in children*. In: Craig R, Mindell J, editors. Health Survey for England 2012.(2013) London: The Health and Social Care Information Centre

33. Sutcliffe SJ, Fox KF, Wood DA *et al.* Incidence of coronary heart disease in a health authority in London: review of a community register. *BMJ* 2003; 326: 20.

34. Bamford J, Sandercock P, Dennis M *et al.* A prospective study of acute cerebrovascular disease in the community: the Oxfordshire Community Stroke Project 1981-86. 1. Methodology, demography and incident cases of first-ever stroke. *J Neurol Neurosurg Psychiatry* 1988; 51: 1373-1380.

35. Cowie MR, Wood DA, Coats AJ *et al.* Incidence and aetiology of heart failure; a population-based study. *Eur Heart J* 1999; 20: 421-428.

36. Sharma M, Nazareth I, Petersen I Trends in incidence, prevalence and prescribing in type 2 diabetes mellitus between 2000 and 2013 in primary care: a retrospective cohort study. *BMJ Open* 2016; 6: e010210.

37. Cancer Research UK. Lung cancer incidence. 2016. [accessed 26 October 2016.] Available from: http://www.cancerresearchuk.org/sites/default/files/cstream-node/cases_crude_lung_2.xls.

38. Cancer Research UK. Bowel cancer incidence. 2016. [accessed 26 October 2016.] Available from: http://www.cancerresearchuk.org/sites/default/files/cstream-node/cases_crude_bowel_2.xls.

39. Cancer Research UK. Breast cancer statistics. 2016. [accessed 26 October 2016.] Available from: http://www.cancerresearchuk.org/sites/default/files/cstream-node/cases_crude_f_breast_2.xls.

For peer review only

For peer review only - http://bmjopen.bmj.com/site/about/guidelines.xhtml

40. Hurley SF, Matthews JP The Quit Benefits Model: a Markov model for assessing the health benefits and health care cost savings of quitting smoking. *Cost Eff Resour Alloc* 2007; 5: 2.

41. Smolina K, Wright FL, Rayner M *et al.* Determinants of the decline in mortality from acute myocardial infarction in England between 2002 and 2010: linked national database study. *BMJ* 2012; 344: d8059.

42. Bamford J, Sandercock P, Dennis M *et al.* A prospective study of acute cerebrovascular disease in the community: the Oxfordshire Community Stroke Project--1981-86. 2. Incidence, case fatality rates and overall outcome at one year of cerebral infarction, primary intracerebral and subarachnoid haemorrhage. *J Neurol Neurosurg Psychiatry* 1990; 53: 16-22.

43. MacIntyre K, Capewell S, Stewart S *et al.* Evidence of improving prognosis in heart failure: trends in case fatality in 66 547 patients hospitalized between 1986 and 1995. *Circulation* 2000; 102: 1126-1131.

44. Seshasai SR, Kaptoge S, Thompson A *et al.* Diabetes mellitus, fasting glucose, and risk of cause-specific death. *N Engl J Med* 2011; 364: 829-841.

45. Bronnum-Hansen H, Davidsen M, Thorvaldsen P *et al.* Long-term survival and causes of death after stroke. *Stroke* 2001; 32: 2131-2136.

46. Office for National Statistics. Cancer survival in England - adults diagnosed. 2016. [accessed 26 October 2016.] Available from: http://www.ons.gov.uk/file?uri=/peoplepopulationandcommunity/healthandsocialcare/conditionsanddiseases/datasets/cancersurvivalratescancersurvivalinenglandadultsdiagnosed/20102014/finalreferencetablev2.xls.

47. Mears R, Jago R Effectiveness of after-school interventions at increasing moderate-to-vigorous physical activity levels in 5- to 18-year olds: a systematic review and meta-analysis. *Br J Sports Med* 2016; 50: 1315-1324.

48. Ward S, Lloyd Jones M, Pandor A *et al.* A systematic review and economic evaluation of statins for the prevention of coronary events. *Health Technol Assess* 2007; 11: 1-160, iii-iv.

For peer review only - http://bmjopen.bmj.com/site/about/guidelines.xhtml

For peer review only

49. Clarke P, Gray A, Legood R *et al.* The impact of diabetes-related complications on healthcare costs: results from the United Kingdom Prospective Diabetes Study (UKPDS Study No. 65). *Diabet Med* 2003; 20: 442-450.

50. Farmer AJ, Wade AN, French DP *et al.* Blood glucose self-monitoring in type 2 diabetes: a randomised controlled trial. *Health Technol Assess* 2009; 13: iii-iv, ix-xi, 1-50.

51. Madan J, Rawdin A, Stevenson M *et al.* A rapid-response economic evaluation of the UK NHS Cancer Reform Strategy breast cancer screening program extension via a plausible bounds approach. *Value Health* 2010; 13: 215-221.

52. Tappenden P, Chilcott J, Eggington S *et al.* Option appraisal of population-based colorectal cancer screening programmes in England. *Gut* 2007; 56: 677-684.

53. Curtis L, Burns A, editors (2015) Unit Costs of Health and Social Care 2015. Canterbury: Personal Social Services Research Unit, University of Kent.

54. Sullivan PW, Slejko JF, Sculpher MJ *et al.* Catalogue of EQ-5D scores for the United Kingdom. *Med Decis Making* 2011; 31: 800-804.

55. Craig R, Mindell J (2013) *Health Survey for England 2012*. In: Craig R, Mindell J, editors.(2013) London: The Health and Social Care Information Centre

56. Jago R, Sebire SJ, Davies B *et al.* Randomised feasibility trial of a teaching assistant led extracurricular physical activity intervention for 9 to 11 year olds: Action 3:30. *Int J Behav Nutr Phys Act* 2014; 11: 114.

57. Sutherland R, Reeves P, Campbell E *et al.* Cost effectiveness of a multi-component school-based physical activity intervention targeting adolescents: the 'Physical Activity 4 Everyone' cluster randomized trial. *Int J Behav Nutr Phys Act* 2016; 13: 94.

58. The Campbell and Cochrane Economics Methods Group (CCEMG) and the Evidence for Policy and Practice Information and Coordinating Centre (EPPI-Centre). CCEMG - EPPI-Centre Cost Converter (v1.5). [accessed 31 January 2017.] Available from: http://eppi.ioe.ac.uk/costconversion/default.aspx.

For peer review only - http://bmjopen.bmj.com/site/about/guidelines.xhtml

59. Siebert U, Alagoz O, Bayoumi AM *et al.* State-transition modeling: a report of the ISPOR-SMDM Modeling Good Research Practices Task Force--3. *Value Health* 2012; 15: 812-820.

60. Fenwick E, Marshall DA, Levy AR *et al.* Using and interpreting cost-effectiveness acceptability curves: an example using data from a trial of management strategies for atrial fibrillation. *BMC Health Serv Res* 2006; 6: 52.

61. Briggs A, Claxton K, Sculpher MJ (2006) *Chapter 4: Making decision models probabilistic*. Decision Modelling for Health Economic Evaluation.(2006): Oxford University Press. pp. 77-120.

62. Pringle A, Cooke C, Gilson N *et al.* Cost-effectiveness of interventions to improve moderate physical activity: A study in nine UK sites. *Health Education Journal* 2010; 69: 211-224.

63. Matrix Research and Consulting. *Modeling the cost effectiveness of physical activity interventions*. London: National Institute for Health and Clinical Excellence, 2006.

64. Lee BY, Adam A, Zenkov E *et al.* Modeling The Economic And Health Impact Of Increasing Children's Physical Activity In The United States. *Health Aff (Millwood)* 2017; 36: 902-908.

65. Pearson N, Braithwaite RE, Biddle SJ *et al.* Associations between sedentary behaviour and physical activity in children and adolescents: a meta-analysis. *Obes Rev* 2014; 15: 666-675.

66. Teychenne M, Ball K, Salmon J Physical activity and likelihood of depression in adults: a review. *Prev Med* 2008; 46: 397-411.

67. Mammen G, Faulkner G Physical activity and the prevention of depression: a systematic review of prospective studies. *Am J Prev Med* 2013; 45: 649-657.

68. Shah AD, Langenberg C, Rapsomaniki E *et al.* Type 2 diabetes and incidence of a wide range of cardiovascular diseases: a cohort study in 1.9 million people. *Lancet* 2015; 385 Suppl 1: S86.

69. Cobiac LJ, Vos T, Barendregt JJ Cost-effectiveness of interventions to promote physical activity: a modelling study. *PLoS Med* 2009; 6: e1000110.

70. Gc VS, Suhrcke M, Hardeman W *et al.* Cost-Effectiveness and Value of Information Analysis of Brief Interventions to Promote Physical Activity in Primary Care. *Value Health* 2018; 21: 18-26.

For peer review only - http://bmjopen.bmj.com/site/about/guidelines.xhtml

71. Böckerman P, Hyytinen A, Kaprio J *et al.* If you drink, don't smoke: Joint associations between risky health behaviors and labor market outcomes. *Soc Sci Med* 2018; 207: 55-63.

For peer review only

For peer review only - http://bmjopen.bmj.com/site/about/guidelines.xhtml

Figure title and legend

Figure 1: Conceptual overview of the model

Figure 2: Scatter plot of incremental costs and QALYs for each intervention, relative to no intervention

Figure 3: Cost-effectiveness acceptability curves

For peer review only

For peer review only - http://bmjopen.bmj.com/site/about/guidelines.xhtml

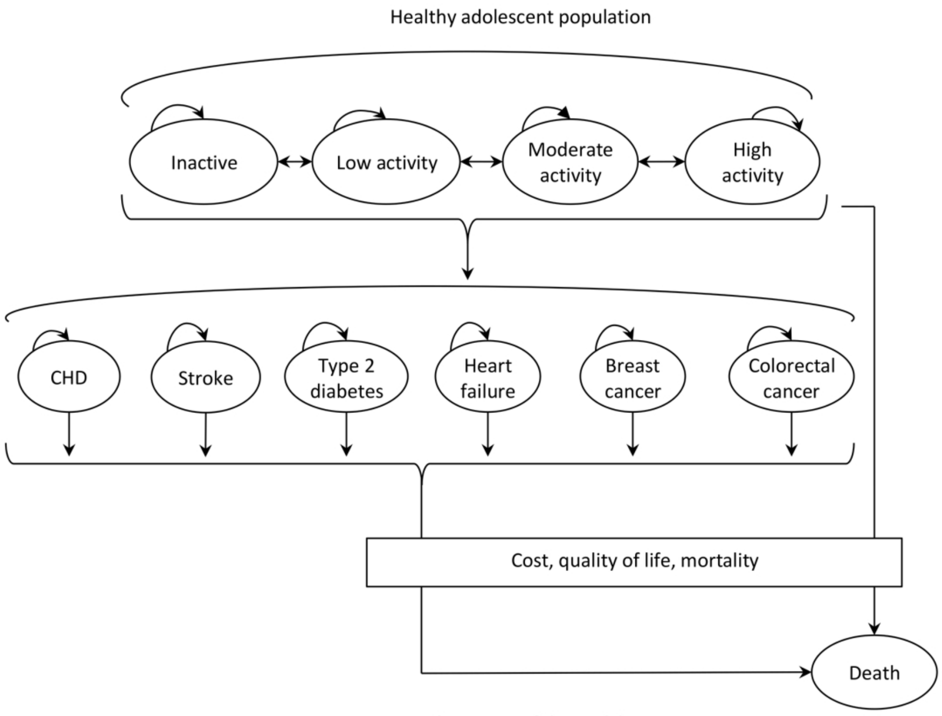

Conceptual overview of the model

168x125mm (300 x 300 DPI)

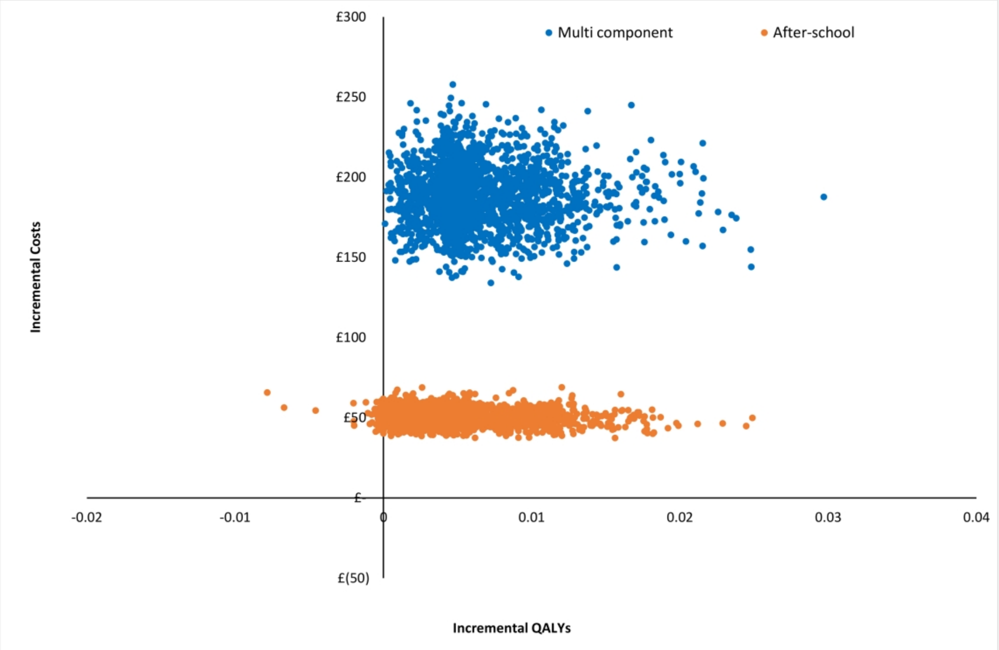

Scatter plot of incremental costs and QALYs for each intervention, relative to no intervention

259x168mm (300 x 300 DPI)

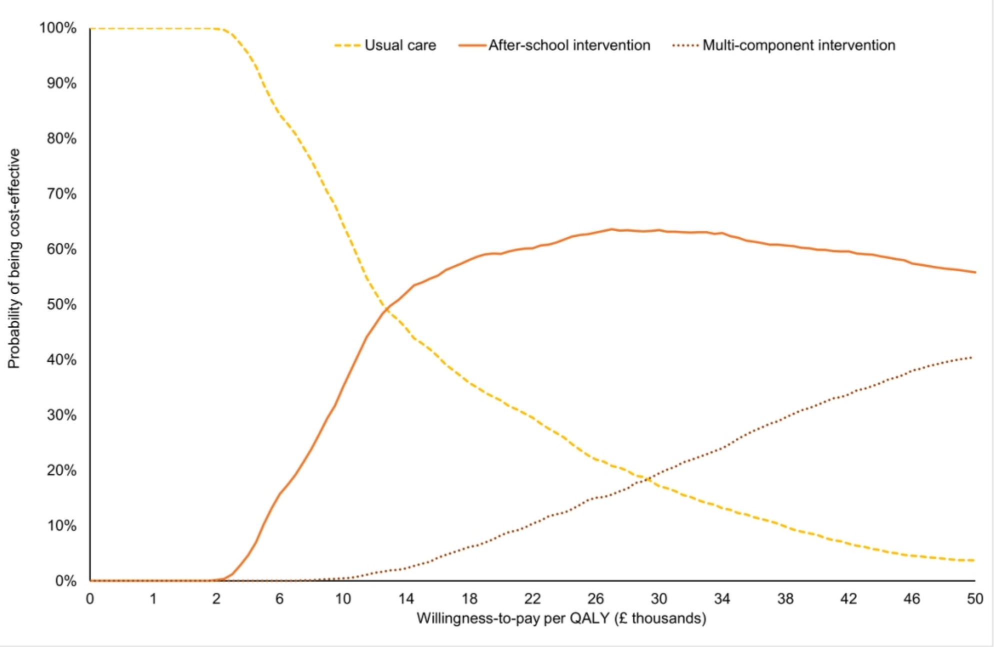

Cost-effectiveness acceptability curves

111x72mm (300 x 300 DPI)

Online Supplemental File

A. Model input parameters

Table S1: Physical activity at baseline and estimates of disease incidence

Parameters	Male				Female				Distribution	Source
	Inactive	Low	Moderate	High	Inactive	Low	Moderate	High		
Baseline PA levels	12%	7%	25%	56%	23%	35%	20%	22%	Fixed	[1]
Relative risk (95% CI) estimates used to adjust disease incidence										
CHD	1.0	0.87 (0.80–0.95)	0.78 (0.74–0.82)	0.70 (0.66–0.75)	1.0	0.87 (0.80–0.95)	0.78 (0.80–0.95)	0.70 (0.80–0.95)	lognormal	[2]
Stroke	1.0	0.85 (0.80–0.91)	0.81 (0.74–0.88)	0.76 (0.68–0.85)	1.0	0.85 (0.80–0.91)	0.81 (0.74–0.88)	0.76 (0.68–0.85)	lognormal	[2]
Heart failure	1.0	0.85 (0.79–0.92)	0.78 (0.75–0.82)	0.70 (0.67–0.73)	1.0	0.85 (0.79–0.92)	0.78 (0.75–0.82)	0.70 (0.67–0.73)	lognormal	[3]
Type 2 diabetes	1.0	0.93 (0.92–0.95)	0.75 (0.69–0.80)	0.60 (0.51–0.70)	1.0	0.93 (0.92–0.95)	0.75 (0.69–0.80)	0.60 (0.51–0.70)	lognormal	[4]
Breast cancer	-	-	-	-	1.0	0.97 (0.94–0.998)	0.94 (0.90–0.98)	0.86 (0.83–0.90)	lognormal	[5]
Colorectal cancer	1.0	0.90 (0.85–0.95)	0.83 (0.77–0.97)	0.79 (0.74–0.85)	1.0	0.90 (0.85–0.95)	0.83 (0.77–0.97)	0.79 (0.74–0.85)	lognormal	[5]

For peer review only - http://bmjopen.bmj.com/site/about/guidelines.xhtml
For peer review only

Parameters	Male				Female				Distribution	Source
	Inactive	Low	Moderate	High	Inactive	Low	Moderate	High		
CHD incidence										[6]
<35	<0.01%	<0.01%	<0.01%	<0.01%	<0.01%	<0.01%	<0.01%	<0.01%	beta	
35-44	0.01%	0.00%	<0.01%	<0.01%	<0.01%	<0.01%	<0.01%	<0.01%	beta	
45-54	0.10%	0.09%	0.08%	0.07%	0.02%	0.01%	0.01%	0.01%	beta	
55-64	0.41%	0.36%	0.32%	0.29%	0.11%	0.10%	0.09%	0.08%	beta	
65-74	1.08%	0.94%	0.84%	0.76%	0.44%	0.38%	0.34%	0.31%	beta	
75+	1.67%	1.45%	1.30%	1.17%	0.67%	0.59%	0.53%	0.47%	beta	
Stroke incidence										[7]
<35	<0.01%	<0.01%	<0.01%	<0.01%	<0.01%	<0.01%	<0.01%	<0.01%	beta	
35-44	<0.01%	<0.01%	<0.01%	<0.01%	0.01%	0.01%	0.01%	0.01%	beta	
45-54	0.01%	0.01%	<0.01%	<0.01%	0.01%	0.01%	0.01%	0.01%	beta	
55-64	0.03%	0.03%	0.03%	0.02%	0.02%	0.02%	0.02%	0.02%	beta	
65-74	0.08%	0.07%	0.07%	0.06%	0.05%	0.05%	0.04%	0.04%	beta	
75-84	0.41%	0.35%	0.33%	0.31%	0.28%	0.23%	0.22%	0.21%	beta	
Heart failure incidence										[8]
<35	<0.01%	<0.01%	<0.01%	<0.01%	<0.01%	<0.01%	<0.01%	<0.01%	beta	
35-44	0.03%	0.02%	0.02%	0.02%	0.03%	0.02%	0.02%	0.02%	beta	
45-54	0.04%	0.03%	0.03%	0.03%	0.01%	0.01%	0.01%	0.01%	beta	
55-64	0.21%	0.18%	0.16%	0.14%	0.08%	0.07%	0.07%	0.06%	beta	
65-74	0.48%	0.41%	0.37%	0.33%	0.28%	0.24%	0.22%	0.19%	beta	
75-84	1.14%	0.97%	0.89%	0.80%	0.64%	0.55%	0.50%	0.45%	beta	

For peer review only

Parameters	Male				Female				Distribution	Source
	Inactive	Low	Moderate	High	Inactive	Low	Moderate	High		
Type 2 diabetes incidence										[9]
<20	0.01%	0.01%	0.01%	0.01%	0.03%	0.03%	0.03%	0.03%	beta	
20–29	0.05%	0.04%	0.04%	0.03%	0.14%	0.12%	0.11%	0.10%	beta	
30–39	0.17%	0.15%	0.13%	0.13%	0.24%	0.20%	0.18%	0.17%	beta	
40–49	0.51%	0.44%	0.38%	0.37%	0.38%	0.32%	0.28%	0.27%	beta	
50–59	0.99%	0.85%	0.74%	0.71%	0.67%	0.57%	0.50%	0.48%	beta	
60–69	1.43%	1.23%	1.07%	1.03%	1.02%	0.88%	0.76%	0.74%	beta	
70–79	1.53%	1.31%	1.15%	1.11%	1.23%	1.06%	0.92%	0.89%	beta	
80–89	1.05%	0.90%	0.79%	0.76%	0.87%	0.74%	0.65%	0.63%	beta	
Breast cancer incidence										[10]
<25					<0.01%	<0.01%	<0.01%	<0.01%	beta	
25 to 29					0.01%	0.01%	0.01%	0.01%	beta	
30 to 34					0.03%	0.03%	0.03%	0.02%	beta	
35 to 39					0.07%	0.07%	0.06%	0.05%	beta	
40 to 44					0.13%	0.13%	0.12%	0.10%	beta	
45 to 49					0.24%	0.23%	0.21%	0.19%	beta	
50 to 54					0.30%	0.29%	0.27%	0.23%	beta	
55 to 59					0.29%	0.28%	0.27%	0.23%	beta	
60 to 64					0.38%	0.36%	0.34%	0.29%	beta	
65 to 69					0.44%	0.42%	0.40%	0.34%	beta	
70 to 74					0.37%	0.35%	0.33%	0.29%	beta	
75 to 79					0.40%	0.39%	0.37%	0.32%	beta	
80 to 84					0.44%	0.42%	0.40%	0.34%	beta	

For peer review only - http://bmjopen.bmj.com/site/about/guidelines.xhtml
For peer review only

Parameters	Male				Female				Distribution	Source
	Inactive	Low	Moderate	High	Inactive	Low	Moderate	High		
Colorectal cancer incidence										[11]
<30	<0.01%	<0.01%	<0.01%	<0.01%	<0.01%	<0.01%	<0.01%	<0.01%	beta	
30 to 34	0.01%	0.01%	0.01%	0.01%	0.01%	0.01%	0.01%	0.01%	beta	
35 to 39	0.01%	0.01%	0.01%	0.01%	0.01%	0.01%	0.01%	0.01%	beta	
40 to 44	0.01%	0.01%	0.01%	0.01%	0.01%	0.01%	0.01%	0.01%	beta	
45 to 49	0.03%	0.03%	0.02%	0.02%	0.02%	0.02%	0.02%	0.02%	beta	
50 to 54	0.06%	0.05%	0.05%	0.05%	0.04%	0.04%	0.04%	0.03%	beta	
55 to 59	0.10%	0.09%	0.08%	0.08%	0.07%	0.06%	0.06%	0.06%	beta	
60 to 64	0.19%	0.17%	0.15%	0.15%	0.11%	0.10%	0.09%	0.09%	beta	
65 to 69	0.26%	0.23%	0.21%	0.20%	0.15%	0.13%	0.12%	0.12%	beta	
70 to 74	0.37%	0.34%	0.31%	0.29%	0.22%	0.20%	0.18%	0.17%	beta	
75 to 79	0.44%	0.40%	0.37%	0.35%	0.26%	0.24%	0.22%	0.21%	beta	
80 to 84	0.54%	0.49%	0.45%	0.43%	0.34%	0.31%	0.28%	0.27%	beta	

Inactive: <30; Low: 30-149; Moderate: 150-420 and High: ≥421 MVPA min per week

For peer review only

Table S2: Estimates of mortality parameters

Parameter	Value				Distribution	Source
	Male Mean	Male SE	Female mean	Female SE		
CHD case fatality rate						[6]
<54 years	0.129	0.013	0.1245	0.0125	beta	
55-64 years	0.132	0.013	0.1597	0.0160	beta	
65-74 years	0.177	0.018	0.2235	0.0224	beta	
75-84 years	0.244	0.024	0.3009	0.0301	beta	
85+ years	0.315	0.032	0.3668	0.0367	beta	
Stroke case fatality rate	0.1730	0.0152	0.1730	0.0152	beta	[12]
Heart failure case fatality rate						[13]
<35 years	0.0000	0.0000	0.0000	0.0000	beta	
35-55 years	0.0989	0.0050	0.0306	0.0001	beta	
55-64 years	0.1253	0.0037	0.1094	0.0001	beta	
65-74 years	0.1666	0.0028	0.1197	0.0001	beta	
75-84 years	0.1989	0.0027	0.1262	0.0001	beta	
85+ years	0.2279	0.0041	0.1240	0.0001	beta	
Death rate after 1st nonfatal CHD						[14]
<45 years	0.0130	0.0020	0.0130	0.0050	beta	
45-54 years	0.0170	0.0010	0.0260	0.0030	beta	
55-64 years	0.0380	0.0020	0.0350	0.0020	beta	
65-74 years	0.0830	0.0020	0.0820	0.0030	beta	
75-84 years	0.1430	0.0040	0.1590	0.0040	beta	
85+ years	0.2620	0.0110	0.2660	0.0080	beta	
SMR for patients after a 1st nonfatal stroke	2.40	0.08	2.21	0.10	Lognormal	[15]
Death rate after 1st nonfatal heart failure						[13]
<55 years	0.0306	0.0001	0.0306	0.0001	beta	
55-64 years	0.1094	0.0001	0.1094	0.0001	beta	
65-74 years	0.1197	0.0001	0.1197	0.0001	beta	
75-84 years	0.1262	0.0001	0.1262	0.0001	beta	
85+ years	0.1240	0.0001	0.1240	0.0001	beta	
HR for death among patients with type 2 diabetes	1.80	0.0269	1.80	0.0269	lognormal	[16]
Breast cancer mortality	–	–	0.0290	0.0004	beta	[17]
Colorectal cancer mortality	0.1038	0.0006	0.1035	0.0006	beta	[17]
Lung cancer mortality	0.3523	0.0004	0.3112	0.0005	beta	[17]

HR = Hazard ratio; SMR = Standardised mortality ratio

For peer review only - http://bmjopen.bmj.com/site/about/guidelines.xhtml

B. Model validation results

1. Cohort profile

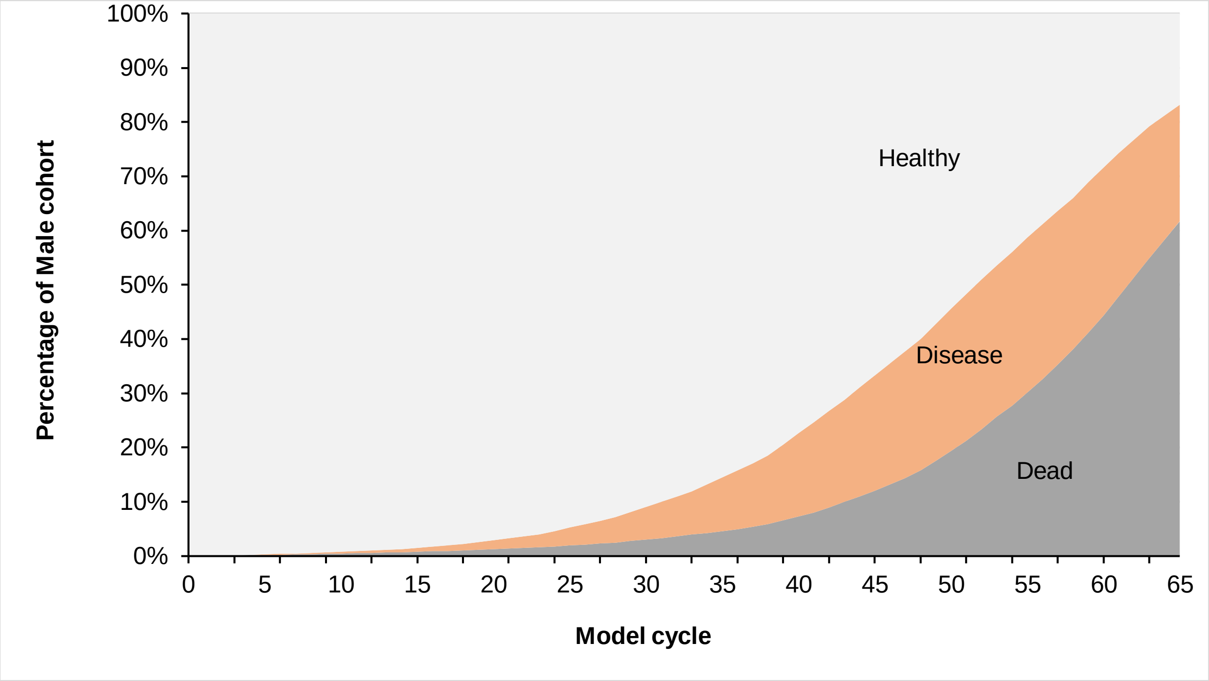

Figure S1: Cohort profile (male)

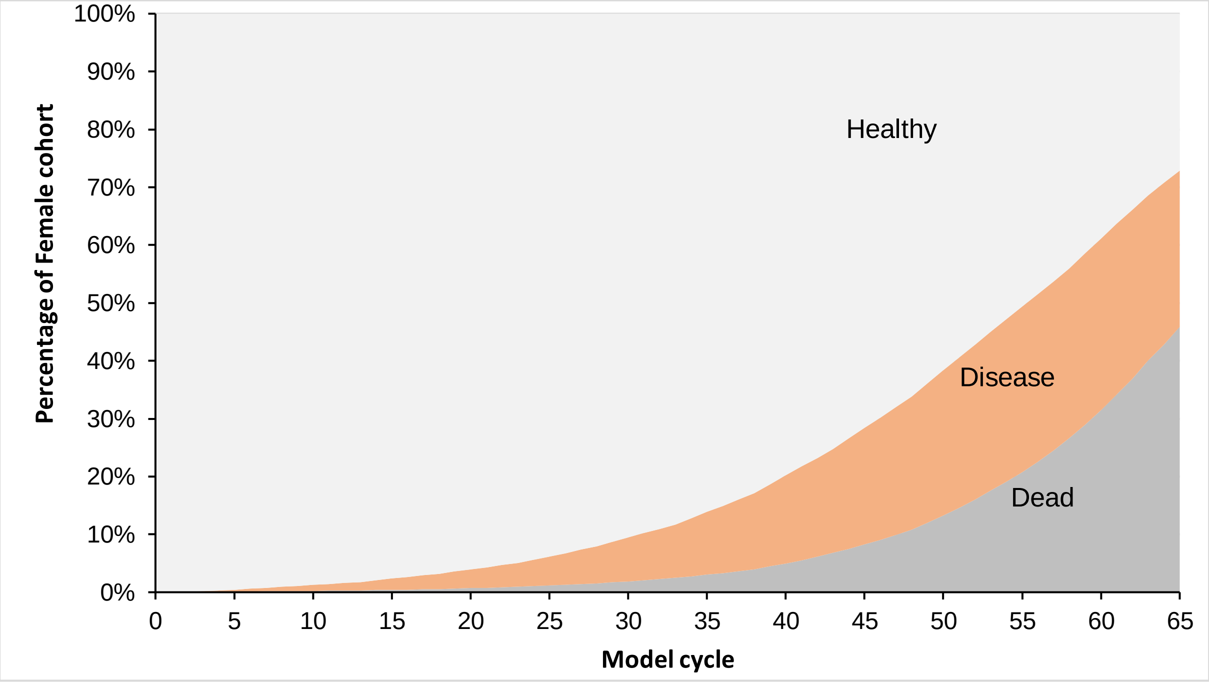

Figure S2: Cohort profile (female)

For peer review only - http://bmjopen.bmj.com/site/about/guidelines.xhtml

2. Prevalence of physical activity levels by age group and gender

Figure S3: Physical activity levels by age group in Male (HSE 2012 data vs no intervention 'control')

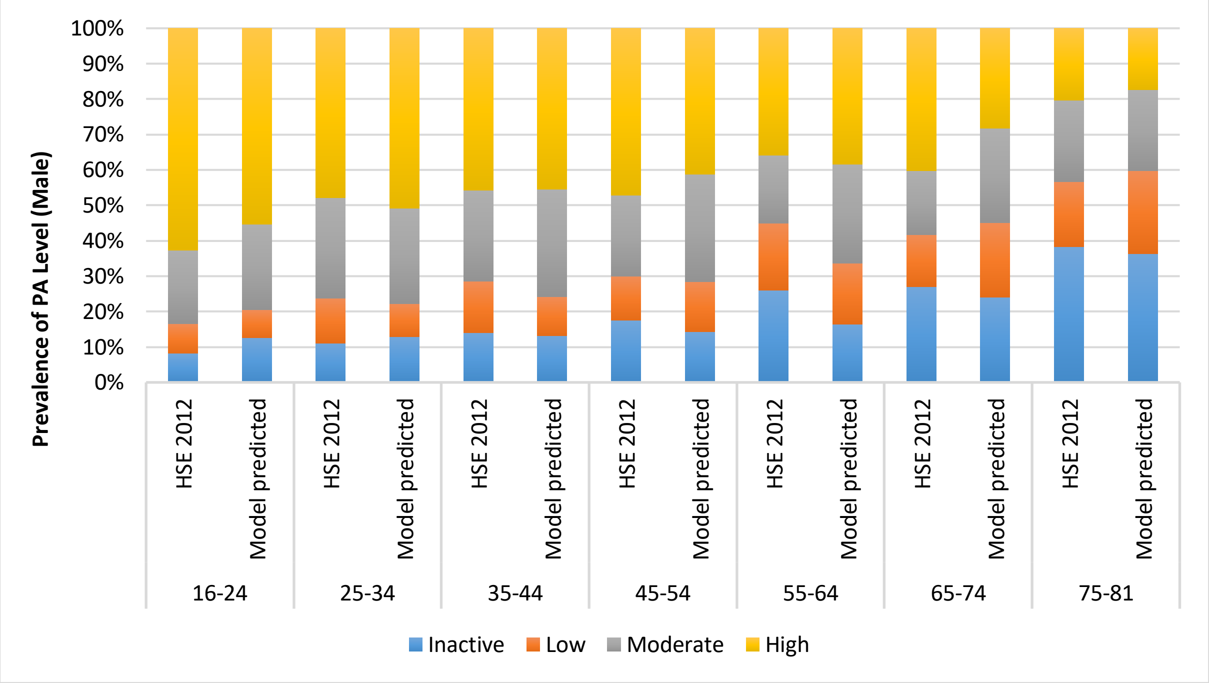

Figure S4: Physical activity levels by age group in Female (HSE 2012 data vs no intervention 'control')

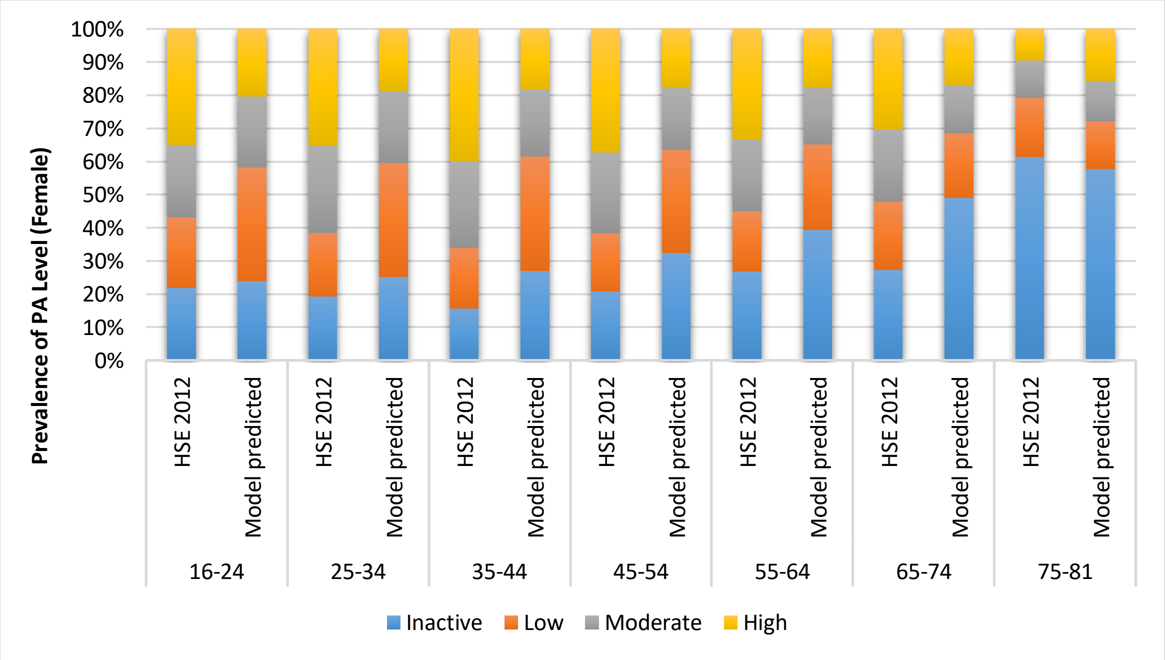

3. Risk of disease events

Figure S5: CHD incidence: model predicted versus data informing model parameter by age-group and gender

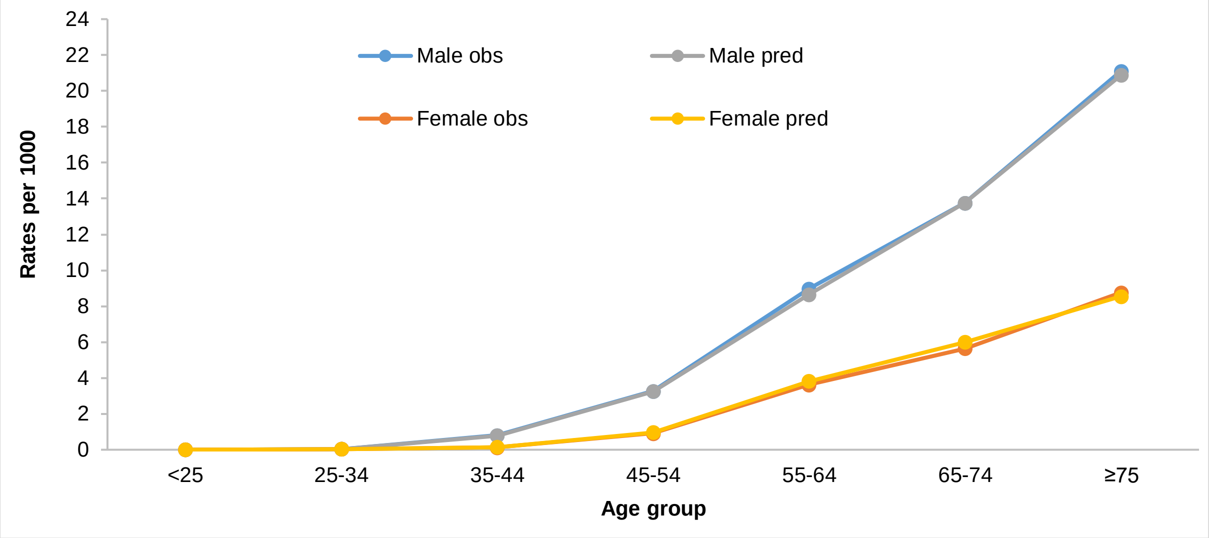

Figure S6: Stroke incidence: model predicted versus data informing model parameter by age-group and gender

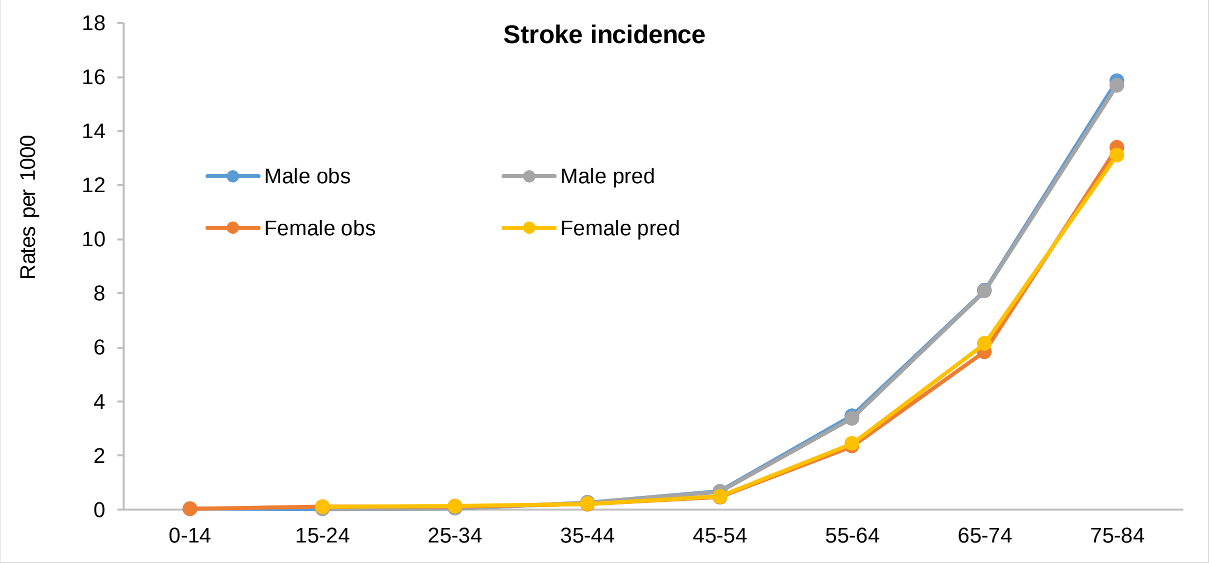

For peer review only - http://bmjopen.bmj.com/site/about/guidelines.xhtml

Figure S7: Heart failure incidence: model predicted versus data informing model parameter by age-group and gender

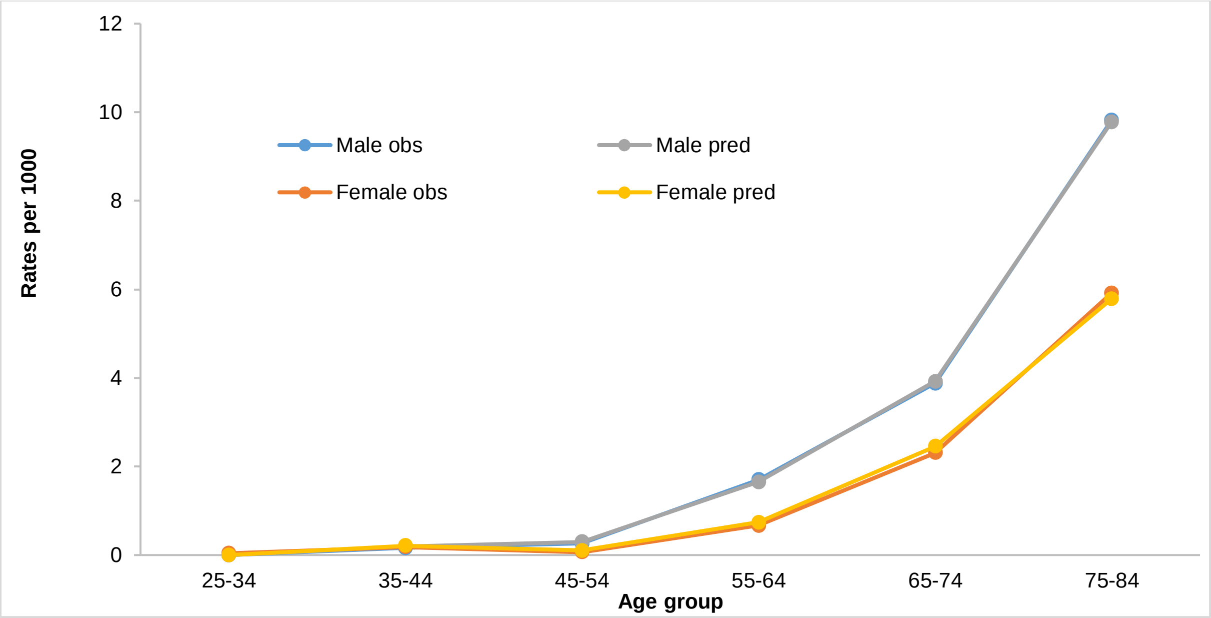

Figure S8: Incidence of type 2 diabetes: model predicted versus data informing model parameter by age-group and gender

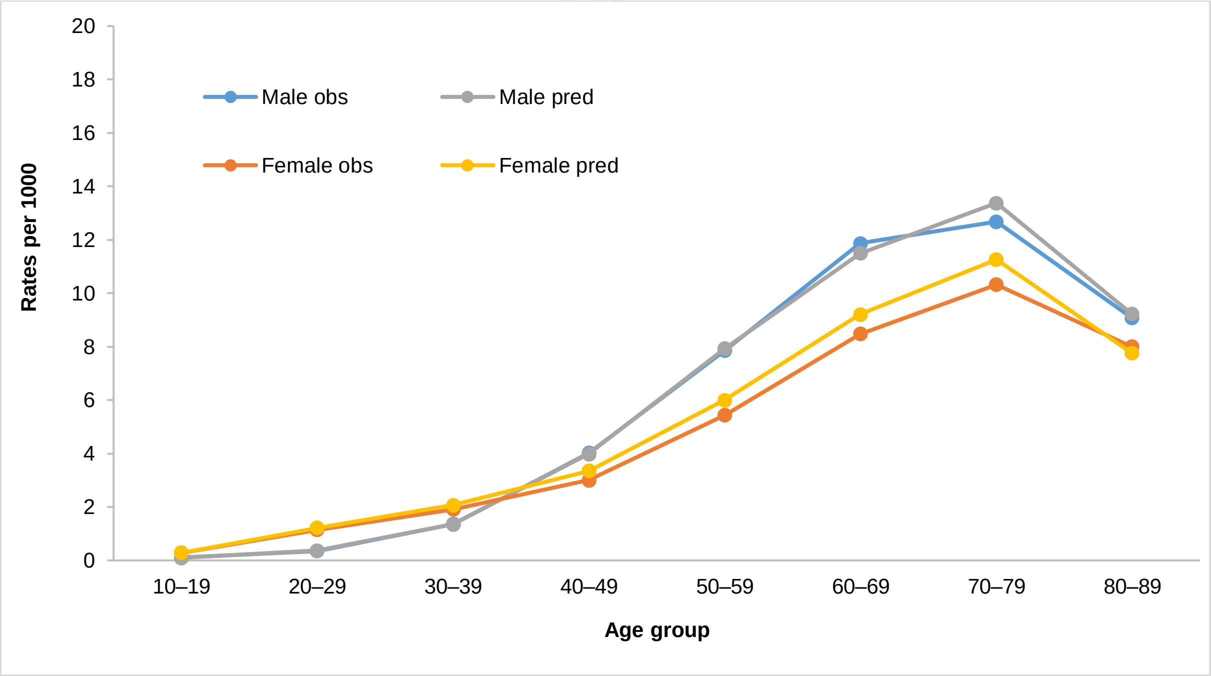

For peer review only - http://bmjopen.bmj.com/site/about/guidelines.xhtml

Figure S9: Breast cancer (female) incidence: model predicted versus data informing model parameter by age-group and gender

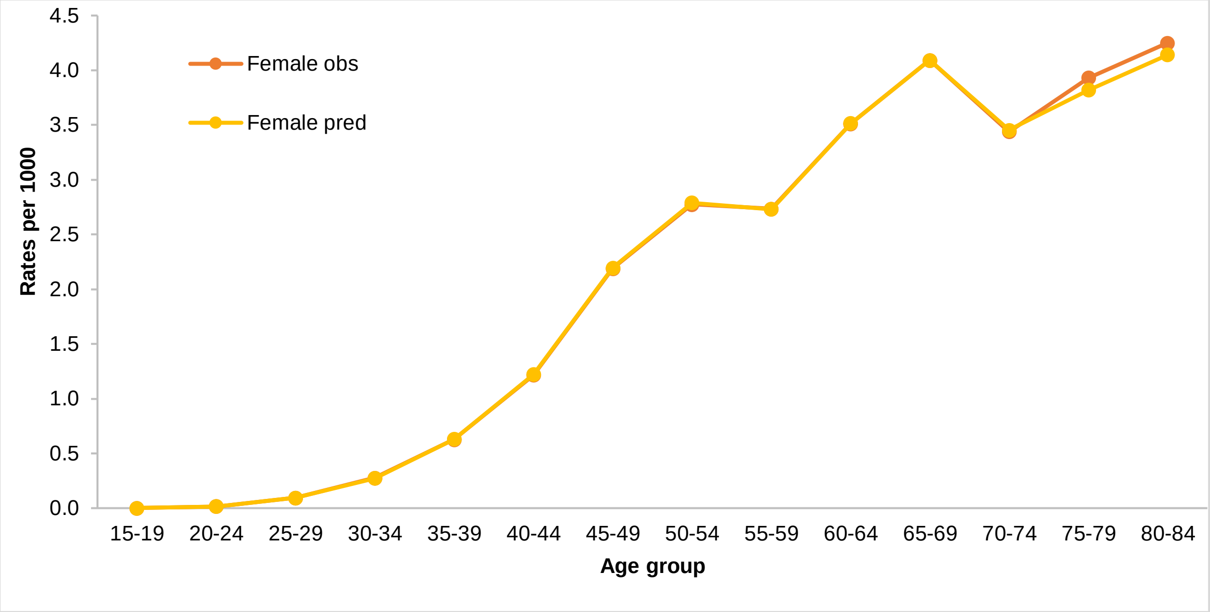

Figure S10: Colorectal cancer incidence: model predicted versus data informing model parameter by age-group and gender

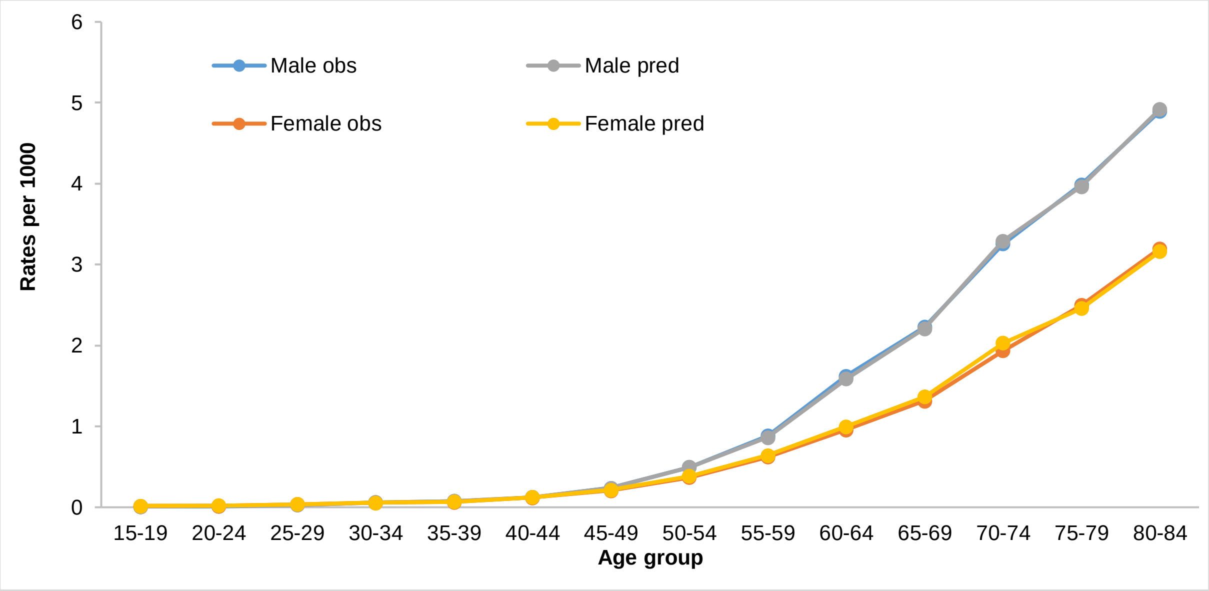

For peer review only - http://bmjopen.bmj.com/site/about/guidelines.xhtml

References

1. Scholes S, Mindell J (2013) *Physical activity in children*. In: Craig R, Mindell J, editors. Health Survey for England 2012.(2013) London: The Health and Social Care Information Centre

2. Wahid A, Manek N, Nichols M *et al.* Quantifying the Association Between Physical Activity and Cardiovascular Disease and Diabetes: A Systematic Review and Meta-Analysis. *J Am Heart Assoc* 2016; 5.

3. Pandey A, Garg S, Khunger M *et al.* Dose-Response Relationship Between Physical Activity and Risk of Heart Failure: A Meta-Analysis. *Circulation* 2015; 132: 1786-1794.

4. Smith AD, Crippa A, Woodcock J *et al.* Physical activity and incident type 2 diabetes mellitus: a systematic review and dose-response meta-analysis of prospective cohort studies. *Diabetologia* 2016.

5. Kyu HH, Bachman VF, Alexander LT *et al.* Physical activity and risk of breast cancer, colon cancer, diabetes, ischemic heart disease, and ischemic stroke events: systematic review and dose-response meta-analysis for the Global Burden of Disease Study 2013. *BMJ* 2016; 354: i3857.

6. Smolina K, Wright FL, Rayner M *et al.* Determinants of the decline in mortality from acute myocardial infarction in England between 2002 and 2010: linked national database study. *BMJ* 2012; 344: d8059.

7. Bamford J, Sandercock P, Dennis M *et al.* A prospective study of acute cerebrovascular disease in the community: the Oxfordshire Community Stroke Project 1981-86. 1. Methodology, demography and incident cases of first-ever stroke. *J Neurol Neurosurg Psychiatry* 1988; 51: 1373-1380.

8. Cowie MR, Wood DA, Coats AJ *et al.* Incidence and aetiology of heart failure; a population-based study. *Eur Heart J* 1999; 20: 421-428.

9. Sharma M, Nazareth I, Petersen I Trends in incidence, prevalence and prescribing in type 2 diabetes mellitus between 2000 and 2013 in primary care: a retrospective cohort study. *BMJ Open* 2016; 6: e010210.

10. Cancer Research UK. Breast cancer statistics. 2016. [accessed 26 October 2016.] Available from: http://www.cancerresearchuk.org/sites/default/files/cstream-node/cases_crude_f_breast_2.xls.

11. Cancer Research UK. Bowel cancer incidence. 2016. [accessed 26 October 2016.] Available from: http://www.cancerresearchuk.org/sites/default/files/cstream-node/cases_crude_bowel_2.xls.

12. Bamford J, Sandercock P, Dennis M *et al.* A prospective study of acute cerebrovascular disease in the community: the Oxfordshire Community Stroke Project--1981-86. 2. Incidence, case fatality rates and overall outcome at one year of cerebral infarction, primary intracerebral and subarachnoid haemorrhage. *J Neurol Neurosurg Psychiatry* 1990; 53: 16-22.

For peer review only - http://bmjopen.bmj.com/site/about/guidelines.xhtml

13. MacIntyre K, Capewell S, Stewart S *et al.* Evidence of improving prognosis in heart failure: trends in case fatality in 66 547 patients hospitalized between 1986 and 1995. *Circulation* 2000; 102: 1126-1131.

14. Turner D, Raftery J, Cooper K *et al.* The CHD challenge: comparing four cost-effectiveness models. *Value Health* 2011; 14: 53-60.

15. Bronnum-Hansen H, Davidsen M, Thorvaldsen P *et al.* Long-term survival and causes of death after stroke. *Stroke* 2001; 32: 2131-2136.

16. Seshasai SR, Kaptoge S, Thompson A *et al.* Diabetes mellitus, fasting glucose, and risk of cause-specific death. *N Engl J Med* 2011; 364: 829-841.

17. Office for National Statistics. Cancer survival in England - adults diagnosed. 2016. [accessed 26 October 2016.] Available from: http://www.ons.gov.uk/file?uri=/peoplepopulationandcommunity/healthandsocialcare/conditionsanddiseases/datasets/cancersurvivalratescancersurvivalinenglandadultsdiagnosed/20102014/finalreferencetablev2.xls.

For peer review only

For peer review only - http://bmjopen.bmj.com/site/about/guidelines.xhtml

CHEERS statement

Section/Item	Item no.	Recommendation	Reported on page no./line no.
**Title and abstract**			
Title	1	Identify the study as an economic evaluation, or use more specific terms such as ''cost-effectiveness analysis'' and describe the interventions compared.	1
Abstract	2	Provide a structured summary of objectives, perspective, setting, methods (including study design and inputs), results (including base-case and uncertainty analyses), and conclusions.	2
**Introduction**			
Background and objectives	3	Provide an explicit statement of the broader context for the study. Present the study question and its relevance for health policy or practice decisions.	4-5
**Methods**			
Target population and subgroups	4	Describe characteristics of the base-case population and subgroups analysed including why they were chosen.	5
Setting and location	5	State relevant aspects of the system(s) in which the decision(s) need(s) to be made	5
Study perspective	6	Describe the perspective of the study and relate this to the costs being evaluated.	13
Comparators	7	Describe the interventions or strategies being compared and state why they were chosen.	8-9
Time horizon	8	State the time horizon(s) over which costs and consequences are being evaluated and say why appropriate.	6
Discount rate	9	Report the choice of discount rate(s) used for costs and outcomes and say why appropriate	6
Choice of health outcomes	10	Describe what outcomes were used as the measure(s) of benefit in the evaluation and their relevance for the type of analysis performed	9-11
Measurement of effectiveness	11a	Single study–based estimates: Describe fully the design features of the single effectiveness study and why the single study was a sufficient source of clinical effectiveness data.	8-9
	11b	Synthesis-based estimates: Describe fully the methods used for the identification of included studies and synthesis of clinical effectiveness data.	8-9
Measurement and valuation of preference-based outcomes	12	If applicable, describe the population and methods used to elicit preferences for outcomes.	N/A
Estimating resources and costs	13a	Single study–based economic evaluation: Describe approaches used to estimate resource use associated with the alternative interventions. Describe primary or secondary research methods for valuing each resource item in terms of its unit cost. Describe any adjustments made to approximate to opportunity costs.	
	13b	Model-based economic evaluation: Describe approaches and data sources used to estimate resource use associated with model health states. Describe primary or secondary research methods for valuing each resource item in terms of its unit cost. Describe any adjustments made to approximate to opportunity costs.	9-10, Table 1
Currency, price date, and conversion	14	Report the dates of the estimated resource quantities and unit costs. Describe methods for adjusting estimated unit costs to the year of reported costs if necessary. Describe methods for converting costs into a common currency base and the exchange rate.	9

For peer review only - http://bmjopen.bmj.com/site/about/guidelines.xhtml
For peer review only

Section/Item	Item no.	Recommendation	Reported on page no./line no.
Choice of model	15	Describe and give reasons for the specific type of decision-analytic model used. Providing a figure to show model structure is strongly recommended.	5
Assumptions	16	Describe all structural or other assumptions underpinning the decision-analytic model.	5-9 & 11-12
Analytic methods	17	Describe all analytic methods supporting the evaluation. This could include methods for dealing with skewed, missing, or censored data; extrapolation methods; methods for pooling data; approaches to validate or make adjustments (e.g., half-cycle corrections) to a model; and methods for handling population heterogeneity and uncertainty.	5, 11-14
**Results**			
Study parameters	18	Report the values, ranges, references, and if used, probability distributions for all parameters. Report reasons or sources for distributions used to represent uncertainty where appropriate. Providing a table to show the input values is strongly recommended.	Table 1-2, Supplementary Table S1, S2
Incremental costs and outcomes	19	For each intervention, report mean values for the main categories of estimated costs and outcomes of interest, as well as mean differences between the comparator groups. If applicable, report incremental cost-effectiveness ratios.	14-15
Characterizing uncertainty	20a	Single study–based economic evaluation: Describe the effects of sampling uncertainty for estimated incremental cost, incremental effectiveness, and incremental cost-effectiveness, together with the impact of methodological assumptions (such as discount rate, study perspective).	N/A
	20b	Model-based economic evaluation: Describe the effects on the results of uncertainty for all input parameters, and uncertainty related to the structure of the model and assumptions.	15-16
Characterizing heterogeneity	21	If applicable, report differences in costs, outcomes, or cost-effectiveness that can be explained by variations between subgroups of patients with different baseline characteristics or other observed variability in effects that are not reducible by more information.	N/A
**Discussion**			
Study findings, limitations, generalizability, and current knowledge	22	Summarize key study findings and describe how they support the conclusions reached. Discuss limitations and the generalizability of the findings and how the findings fit with current knowledge.	16-18
**Other**			
Source of funding	23	Describe how the study was funded and the role of the funder in the identification, design, conduct, and reporting of the analysis. Describe other nonmonetary sources of support.	19
Conflicts of interest	24	Describe any potential for conflict of interest among study contributors in accordance with journal policy. In the absence of a journal policy, we recommend authors comply with International Committee of Medical Journal Editors' recommendations.	19

For consistency, the CHEERS statement checklist format is based on the format of the CONSORT statement checklist

For peer review only - http://bmjopen.bmj.com/site/about/guidelines.xhtml

BMJ Open

The cost-effectiveness of physical activity interventions in adolescents: model development and illustration using two exemplary interventions

Journal:	*BMJ Open*
Manuscript ID	bmjopen-2018-027566.R2
Article Type:	Research
Date Submitted by the Author:	27-Jun-2019

Complete List of Authors:	Gc, Vijay; University of York Centre for Health Economics, ; University of East Anglia Norwich Medical School,<br>Suhrcke, Marc; University of York Centre for Health Economics; Luxembourg Institute of Socio-Economic Research<br>Atkin, Andrew; University of East Anglia Faculty of Medicine and Health Sciences,<br>van Sluijs, Esther; University of Cambridge, MRC Epidemiology Unit<br>Turner, David; University of East Anglia, Norwich Medical School

**Primary Subject Heading**:	Health economics
Secondary Subject Heading:	Public health, Sports and exercise medicine, Health services research
Keywords:	physical activity, young adult, HEALTH ECONOMICS, cost-effectiveness

SCHOLARONE™
Manuscripts

The cost-effectiveness of physical activity interventions in adolescents: model development and illustration using two exemplary interventions

Vijay S Gc[1,2], Marc Suhrcke[1,3], Andrew J Atkin[4,5], Esther van Sluijs[4], David Turner[2]

[1] Centre for Health Economics, University of York, York, UK

[2] Norwich Medical School, Faculty of Medicine and Health Sciences, University of East Anglia, Norwich, UK

[3] Luxembourg Institute of Socio-Economic Research (LISER), Esch-sur-Alzette/Belval, Luxembourg

[4] MRC Epidemiology Unit and UKCRC Centre for Diet and Activity Research (CEDAR), University of Cambridge School of Clinical Medicine, Cambridge, UK

[5] School of Health Sciences, Faculty of Medicine and Health Sciences, University of East Anglia, Norwich, UK

Corresponding author:

Dr Vijay S Gc, PhD
Centre for Health Economics
University of York
York, YO10 5DD
United Kingdom
Phone: +44 (0)1904 321973, Email: vijay.gc@york.ac.uk

Keywords: physical activity; adolescent; young adult; cost-effectiveness; economic model

Word count excluding title page, abstract, references, figures and tables: *3,849*

Number of tables and figures: Figures *3*, Tables *3*

For peer review only

Abstract

Objective: To develop a model to assess the long-term costs and health outcomes of physical activity interventions targeting adolescents.

Design: A Markov cohort simulation model was constructed with the intention of being capable of estimating long-term costs and health impacts of changes in activity levels during adolescence. The model parameters were informed by published literature and the analysis took a National Health Service perspective over a lifetime horizon. Univariate and probabilistic sensitivity analyses were undertaken.

Setting: School and community

Participants: A hypothetical cohort of adolescents aged 16 years at baseline.

Interventions: Two exemplar school-based: a comparatively simple, after-school intervention and a more complex multi-component intervention compared to usual care.

Primary and secondary outcome measures: Incremental cost-effectiveness ratio as measured by cost per quality-adjusted life year gained.

Results: The model gave plausible estimates of the long-term effect of changes in physical activity. The use of two exemplar interventions suggests that the model could potentially be used to evaluate a number of different physical activity interventions in adolescents. The key model driver was the degree to which intervention effects were maintained over time.

Conclusions: The model developed here has the potential to assess long-term value for money of physical activity interventions in adolescents. The two applications of the model indicate that complex interventions may not necessarily be the ones considered the most cost-effective when longer-term costs and consequences are taken into account.

Word count: 230 words

For peer review only - http://bmjopen.bmj.com/site/about/guidelines.xhtml

For peer review only

Article summary

Strengths and limitations of this study

- A Markov cohort model was developed based on currently available evidence to simulate the long-term impacts in terms of costs and quality-adjusted life years of physical activity interventions for adolescents.
- The study incorporates the most recent evidence on the effect of increased physical activity in long-term chronic disease conditions.
- The model builds on previously published cohort models and includes additional health states. In addition, extensive sensitivity analyses have been performed to reflect uncertainty in model structure and parameter assumptions.
- A limitation of the present study is that the change in activity level over time were estimated using population-level prevalence data due to unavailability of longitudinal data describing the lifetime trajectory of physical activity and exclusion of long-term impacts on other conditions, for example, mental health.

For peer review only

For peer review only - http://bmjopen.bmj.com/site/about/guidelines.xhtml

Introduction

Insufficient physical activity is a key risk factor for chronic diseases, such as cardiovascular disease (CVD), type 2 diabetes, and some types of cancer in the general population.[1,2] Physical activity in young people is associated with many health benefits including improved cardiovascular and mental health,[3,4] academic performance[5] and bone health.[6] Whilst physical activity typically declines with age, active children are more likely to become active adults.[7,8] Although the short- and long-term health benefits of physical activity are well-documented,[9,10] in England, nearly half of all young people fail to achieve the recommended levels of physical activity, based on self-reports.[11,12] When measured objectively using accelerometers, the prevalence of inactivity is higher still (91% boys and 98% girls).[13]

The high prevalence of physical inactivity in young people places a significant burden on health care services and the wider economy. A 2014 report estimated a lifetime cost of £53.3 billion related to inactivity among today's 11 to 25 year olds,[12] taking into consideration the fact that physical activity levels in childhood predict adult activity levels.[14] This estimate includes direct healthcare costs of treating the burden of type 2 diabetes, coronary heart disease, stroke and colon cancer, and the risk of premature death and morbidity associated with these illnesses.

In recent years, there has been increasing interest in identifying interventions to improve young people's activity levels. Although some school-based physical activity interventions show promising effects[15,16] the existing evidence is very limited in both quantity and quality.[17] While improvements in physical activity may have long-term health benefits, the evidence on the longer-term costs and health benefits of interventions in adolescence is particularly sparse. Trials generally do not have sufficient follow-up to capture associated longer-term costs and consequences directly.[18] Quantifying the economic and health benefits associated with physical activity interventions would help decision makers to make informed decisions, i.e. assessing whether these interventions are an efficient use of limited healthcare resources.

For peer review only - http://bmjopen.bmj.com/site/about/guidelines.xhtml

Furthermore, much of the health benefits of physical activity interventions occur in the future. Also, many interventions are focused on adult or elderly populations. The long-term costs and health benefits of physical activity interventions in an adolescent population are a comparatively scarcely researched area. To fill this critical research gap, we developed a decision analytic model aimed at quantifying the potential long-term costs and quality-adjusted life-year (QALY) implications of changes in activity levels during adolescence. We then illustrate some of the practical implications of taking a longer-term perspective by applying the model to two exemplary intervention programmes to show how the changes in levels of adolescent physical activity could affect activity levels throughout lifetime, as well the resulting longer term costs and health benefits.

Methods

We developed a probabilistic, age- and gender-dependant state-transition Markov model to simulate a cohort of healthy adolescents. The model estimates the risk of cardiovascular, type 2 diabetes and oncological events over a lifetime, and associated costs and quality of life. The model structure was based on the previously published models[19,20] that assessed the cost-effectiveness of physical activity interventions in the adult population. The model combines information from a variety of sources relating to disease and physical activity epidemiology, mortality, effectiveness, health-related quality of life, and costs.

Structuring the model

Model and population type

A simulated cohort of 10,000 healthy adolescents aged 16 entered the model. The intervention is assumed to have been delivered at the start of the first model cycle. At the end of the first cycle, based on the intervention effectiveness evidence, a proportion of cohort members move to a higher activity level. Depending on the sustainability of the intervention effect, in subsequent years cohort

For peer review only - http://bmjopen.bmj.com/site/about/guidelines.xhtml

members obtain an annual probability of remaining at the new activity level or moving to a lower physical activity state or to a disease state or death.

Model states

The health states included in the model are 'healthy' (disease-free), 'having a chronic disease' and 'dead'. At the beginning of the simulation, we assumed that all cohort members start out as healthy, i.e. disease free. Within the model, physical activity is classified into four activity levels (inactive, low, moderate and high) based on weekly moderate-to-vigorous physical activity (MVPA). The model has 11 health states in total: four physical activity levels, six chronic disease conditions and death (Figure 1). Among the healthy, the risk of developing one of the six diseases depends on age, gender and activity level. For simplicity, we assumed that health states included in the model were mutually exclusive, and cohort members did not move between disease states.

The selection of the disease conditions was based on currently available evidence describing the association between physical activity and disease risk,[21-26] and economic and health burden of diseases related to physical inactivity in the UK.[27] Disease conditions are all associated with costs and impact upon quality of life. For each of the selected exemplar interventions, we re-ran the model changing appropriate data to reflect the costs and effectiveness of these interventions. Costs and QALYs were discounted at 3.5% as recommended for the National Institute for Health and Care Excellence (NICE) reference case.[28]

Time horizon

The time horizon of the model is 65 years, i.e. the model follows the cohort of 16 year olds until they reach 81 years – the average life expectancy in the UK.[29] A half-cycle correction was applied under the assumption that each transition happened halfway during the cycle.[30]

For peer review only - http://bmjopen.bmj.com/site/about/guidelines.xhtml

Populating the model

Baseline population and activity levels

Data on age and gender distribution of the initial population were obtained from the Office for National Statistics (ONS).[31] An estimate of baseline activity level (weekly MVPA) was taken from the 2012 Health Survey for England (HSE).[32] Participants were divided into four levels (<30, 30-149, 150-420 and ≥421 MVPA min per week) of activity by age and gender, based on the UK Department of Health's physical activity recommendations.[11] The moderate activity category equates to the current recommended level of physical activity.

Transition probabilities

To estimate the annual probability of developing each disease, the annual incidence rates for the disease conditions included in the model were taken from population-based studies in the UK.[33-39] These are probabilities for the general adult population and included all four activity levels. In order to adjust the differential risk of developing these disease conditions by activity level, we first derived the probability of developing that disease among inactive people, using the method presented by Hurley et al.[40] The probabilities for each condition among low, moderate and high activity levels in the cohort were estimated by multiplying the probabilities for the inactive population by corresponding relative risks (RRs) for low, moderate and high activity.[21-24,26] Supplementary section A Table S1 provides the transitional parameters.

Mortality rates

All-cause mortality rates by age and gender were derived from the ONS.[29] Mortality consists of disease-specific mortality and mortality due to other causes. We estimated other-cause mortality by subtracting the total number of deaths due to the six disease conditions included in the model from the all-cause mortality total. The other-cause mortality rates by age and gender were assumed constant in the sensitivity analysis. The model assumes that a given proportion of coronary heart disease (CHD), stroke and heart failure (HF) events would be immediately fatal and people who

For peer review only - http://bmjopen.bmj.com/site/about/guidelines.xhtml

survived one of these events had an increased subsequent risk of death (Supplementary section A Table S2). Case fatality rates for these health states were taken from published population-based studies in the UK.[41-43]

Individuals with type 2 diabetes were assigned an increased risk of mortality using data from a published meta-analysis.[44] Based on standardised mortality ratios reported in long-term follow-up studies of first-ever patients' stroke, a 2-fold increase in the risk of death after one year[45] was applied to the general mortality rates from the life tables to reflect the higher mortality burden post-vascular event. Annual mortality rates following the first HF event were estimated from ten-year case fatality rates in patients admitted with a principal diagnosis of HF in Scotland.[43] The age-adjusted five-year net survival rates from the ONS[46] were used to estimate an annual risk of cancer death. It was assumed that the mortality rates do not increase due to cancer beyond five years after a cancer diagnosis.

Interventions

We reviewed the literature to identify physical activity interventions targeting the adolescent population. For primarily illustrative purposes, we selected two interventions to test the model and explore the health and economic impact of smaller and greater changes in physical activity. The first was a simple after-school intervention, not costly but likely with smaller benefits[47], the second a more complex, multi-component intervention – more costly intervention but with higher expected benefits[15]:

After school intervention programme. Mears and Jago[47] included six after-school interventions in their meta-analysis and reported the pooled mean difference of 4.84 (-0.94 to 10.61) min of MVPA per day. These programmes typically included structured or unstructured play, planned MVPA, single or multisport physical activity programme or adhering to specific instructions (e.g. maintaining sufficient intensity of exercise during a session).

For peer review only - http://bmjopen.bmj.com/site/about/guidelines.xhtml

Multi-component intervention. The intervention effect for school-based multi-component intervention was taken from a cluster-randomised trial[15] implemented in secondary schools in Australia. The intervention included multiple intervention strategies (e.g. active physical education lessons, enhanced school sport, supportive school physical activity policies) targeting physical activity. The reported difference in min of MVPA per day between the intervention and control arm at follow-up was 7.0 (2.7 to 11.4).

Costs, currency, price date and conversion

The annual costs incurred in each disease health state were based on previously published studies (Table 1). First-year costs and subsequent year costs are assigned for each of the health states modelled. Costs of CHD and stroke were taken from the statins health technology assessment (HTA).[48] Costs for HF were taken from the UKPDS study.[49] Costs for type 2 diabetes were based on Diabetes Glycaemic Education and Monitoring (DiGEM) trial and included medication and other healthcare costs.[50] Cost for breast cancer was taken from a screening appraisal for breast cancer.[51] The estimated cost is the weighted average treatment costs depending on the prognosis at diagnosis. Cost for colorectal cancer was based on a screening appraisal.[52] The appraisal reported the lifetime cost of colorectal cancer according to the cancer stage, and a weighted average cost was estimated using the proportion of cancers identified at each stage. All the costs were inflated to 2013-14.[53]

Health State Utility Values

Utility weights were used to value a year spent in each of the health states used in the model. A value of 1 means that the health state would be equivalent to full health and one year in that state would generate 1 QALY. For example, if an individual spent ten years in the CHD state with a utility of 0.65, they would accrue 6.5 QALYs. The same number of QALYs could be generated by spending 6.5 years in a health state of 1. The lower the utility value, the worse the health state is considered to be. The weights used to value disease health states are given in Table 1 and were taken from

For peer review only - http://bmjopen.bmj.com/site/about/guidelines.xhtml
For peer review only

Sullivan et al.[54], who used UK based community preferences to derive EQ-5D scores from the Medical Expenditure Panel Survey (MEPS). Utility weights for activity level by age- and gender were extracted from the HSE 2012 (Table 2).[55]

Table 1: Health state utilities and costs used in the model

Parameter	Value	Standard error	Distribution	Source
Health state utilities				[54]
CHD	0.65	0.0203	Beta (α=357, β=191)	
Stroke	0.52	0.0192	Beta (α=355, β=323)	
Heart failure	0.49	0.0194	Beta (α=326, β=335)	
Type 2 diabetes	0.66	0.0054	Beta (α=5032, β=2548)	
Breast cancer	0.76	0.0133	Beta (α=791, β=256)	
Colorectal cancer	0.67	0.0314	Beta (α=150, β=73)	
**Costs of health states**				
CHD 1st event	£5,562	£556	Gamma (α=100, β=56)	[48]
CHD subsequent	£214	£21	Gamma (α=100, β=2)	[48]
CHD fatal	£1,458	£146	Gamma (α=100, β=15)	[48]
Stroke 1st event	£10,062	£1,006	Gamma (α=100, β=101)	[48]
Stroke subsequent	£2,705	£270	Gamma (α=100, β=27)	[48]
Stroke fatal	£8,805	£881	Gamma (α=100, β=88)	[48]
Heart failure	£2,402	£240	Gamma (α=100, β=24)	[49]
HF subsequent	£815	£82	Gamma (α=100, β=8)	[49]
Type 2 diabetes	£1,257	£126	Gamma (α=100, β=13)	[50]
Breast cancer	£12,155	£1,215	Gamma (α=100, β=122)	[51]
Colorectal cancer	£16,978	£1,698	Gamma (α=100, β=170)	[52]

For peer review only - http://bmjopen.bmj.com/site/about/guidelines.xhtml

Table 2: Baseline utilities associated with physical activity level

Age-group	Utility values by activity level				Distribution	Source
	Inactive	Low	Moderate	High		
16-34	0.897	0.918	0.937	0.943	beta	[55]
35-44	0.770	0.889	0.914	0.927	beta	[55]
45-54	0.696	0.852	0.899	0.921	beta	[55]
55-64	0.648	0.861	0.863	0.907	beta	[55]
65-74	0.657	0.823	0.870	0.897	beta	[55]
65-74	0.701	0.829	0.850	0.876	beta	[55]

Inactive: <30; Low: 30-149; Moderate: 150-420 and High: ≥421 MVPA min per week

Modelling health benefits

We estimated the probability of moving to a higher activity level after intervention by adding intervention-specific MVPA minutes to baseline levels. We then calculated the proportion of cohort members that moved from one activity level to another, and we used this proportion as a transition probability. Members of the cohort who improved physical activity level at the end of cycle 0 were assumed to have a lower probability of developing type 2 diabets, CHD, stroke, HF or any of the cancers.

Estimation of the sustainability of intervention effect

The decline in activity levels over time in the 'no intervention'-group was modelled based on data from a recent meta-analysis examining the change in activity level from adolescence to adulthood[8] as well as using prevalence data from the 2012 HSE. The meta-analysis showed a decrease of 6.5 min/day of MVPA in boys and 5.5 min/day of MVPA in girls from adolescence to adulthood. As the review included studies reporting at least one measurement between both 13-19 years and 16-30 years, we assumed that the decrease in MVPA minutes was for a seven-year period. For the

For peer review only - http://bmjopen.bmj.com/site/about/guidelines.xhtml

intervention group, a 50% decline in intervention effect per year post-intervention was assumed. Therefore, the activity levels decreased towards the control activity level after seven years. The effect of this was that a number of individuals in the intervention group were re-categorised into higher activity groups immediately after the intervention. Over time, many of these individuals would fall back into lower activity groups, and at the end of seven-year, there was no real difference between intervention and control groups. To account for the decline in physical activity occurring with age across the life course, we estimated age-related activity levels using 2012 HSE data on physical activity prevalence by age and gender. Activity levels were estimated in three broad age groups: 24-44, 45-64 and ≥65 years to reflect activity-level differences in adulthood, middle-age and retirement.

Estimation of costs of intervention

Costs of delivering after-school intervention were taken from a cluster randomised feasibility study in the UK.[56] The authors reported cost estimates (£49 per participant, 2012-13 price) of a teaching assistant led extra-curricular physical activity intervention. We took that as an indicative cost of the after-school intervention, inflated to 2014 prices (£51 per participant). The cost includes intervention delivery costs, one-off training and non-recurrent costs such as consultation and intervention development work.

Sutherland et al. performed an economic evaluation alongside the multi-component intervention [57] and reported that the intervention costed AU$ 394 per participant. This cost includes opportunity costs for delivery of strategies by school staff and community sport and fitness providers, materials and printing. We converted this cost to 2014 pound sterling (£190 per participant) by applying the gross domestic product deflator index (GDP values) and purchasing power parities conversion rates using the CCEMG-EPPI-Centre Cost Converter (V1.5).[58] We added the intervention cost in the first year for the intervention groups.

For peer review only

For peer review only - http://bmjopen.bmj.com/site/about/guidelines.xhtml

Validation

The model structure, data sources and the effectiveness evidence used in the exemplar interventions and model results were validated by the study team comprising health economists, behavioural epidemiologists and trialists. Internal validity of the model code was ensured using several tests and by assuming a constant total population throughout the calculations. Furthermore, model predictions were examined to make sure that results from the model were consistent with the model's specifications. We specifically checked lifetime incidence and mortality, as well as physical activity prevalence by age and gender. Details can be found in the Supplementary material, section B Figures S1-S10.

Cost-effectiveness analysis

We used the NICE reference case[28] and followed existing guidelines for modelling.[59] The analysis was performed from the perspective of the NHS and personal social services. Costs and health outcomes were discounted at 3.5% per year.[28] We estimated the cost-effectiveness ratio for each intervention compared to 'no intervention'. The incremental costs and QALYs gained by the intervention were estimated and averaged across the simulated cohort. The incremental cost-effectiveness ratio (ICER) was estimated as a ratio between the additional expected cost of the intervention, and the additional expected QALYs gained, both relative to the 'no intervention' alternative. The intervention was considered cost-effective if the ICER was no more than the lower NICE recommended threshold of £20,000 per QALY. The uncertainty surrounding the estimates of cost-effectiveness is presented using cost-effectiveness acceptability curves (CEACs). A CEAC shows the probability that an intervention is cost-effective compared with alternative intervention for a range of cost-effectiveness thresholds.[60]

For peer review only

For peer review only - http://bmjopen.bmj.com/site/about/guidelines.xhtml

Sensitivity analyses

We performed sensitivity analyses by changing intervention decay rates and time horizon that affect cost-effectiveness results. Deterministic one-way, scenario and extreme value analyses were undertaken. This was complemented by probabilistic sensitivity analysis (PSA) to assess the combined effects of uncertainty in the input parameters by simultaneously sampling input parameter values from within a specified distribution using Monte Carlo simulations (2,000 iterations). Uncertainty about the sustainability of the intervention effects was assessed by varying the decay rates between 0% and 100%. In our base-case analysis, we assumed that the intervention effects are sustained for the first year but decay exponentially at a rate of 50% per annum thereafter, resulting in virtually no intervention effect after five years.

Probabilities of disease events and utilities were assumed to follow a beta distribution; costs followed a gamma distribution and risk reductions/hazard ratios a lognormal distribution.[61] The model was developed and implemented in Microsoft Excel.

Patient and public involvement

Public involvement informed the questions addressed in the overarching research project of which this study is a part. No further public involvement was sought with regards to the development of the research question, the outcome measures or the study design.

Results

Base-case analysis

In our base-case analysis, both after-school and multi-component interventions were associated with higher costs and were more effective than no intervention (

Table 3). The multi-component intervention was associated with a QALY gain of 0.002 at an incremental cost of £138, compared to the after-school intervention, yielding an ICER of £68,056.

For peer review only - http://bmjopen.bmj.com/site/about/guidelines.xhtml

Table 3: Incremental cost-effectiveness ratios in the base-case and sensitivity analyses

	Total cost (£)	Total QALYs	Incremental cost (£)	Incremental QALY	ICER
**Base-case analysis**					
No intervention	4,441	21.705	–	–	–
After-school intervention	4,491	21.710	50.64	0.004	11,486
Multi-component intervention	4,629	21.712	137.89	0.002	68,056
**No (0%) decay of intervention effects**					
No intervention	4,437	21.707	–	–	–
After-school intervention	4,451	21.898	14.32	0.191	75
Multi-component intervention	4,571	21.987	119.59	0.089	1,342
**33% decay of intervention effects**					
No intervention	4,430	21.705	–	–	–
After-school intervention	4,479	21.718	48.86	0.013	3,661
Multi-component intervention	4,616	21.726	136.94	0.008	17,661
**100% decay of intervention effects**					
No intervention	4,432	21.705	–	–	–
After-school intervention	4,484	21.707	51.42	0.002	28,838
Multi-component intervention	4,621	21.708	137.61	0.001	189,897
**Time horizon (10 years)**					
No intervention	41	7.167	–	–	–
After-school intervention	92	7.170	51.47	0.004	14,204
Multi-component intervention	231	7.172	138.60	0.002	75,100
**Time horizon (20 years)**					
No intervention	219	12.835	–	–	–
After-school intervention	270	12.838	51.09	0.004	13,414
Multi-component intervention	408	12.840	138.19	0.002	72,346

ICER, incremental cost-effectiveness ratio (incremental cost/incremental QALY); QALY, quality-adjusted life year.

For peer review only

Sensitivity analyses

Sensitivity analyses were implemented, varying the base-case assumptions and inputs, as outlined in the methods section (Table 3). Both the after-school and multi-component interventions had more favourable ICERs at lower decay rates, indicating that cost-effectiveness of physical activity interventions depends on the sustainability of intervention effects over time. The results of PSA are presented on the cost-effectiveness plane (Figure 2), with simulations found to lie predominantly in the northeast quadrant indicating – as expected – an improved health outcome but also at higher spending on physical activity interventions.

Figure 3 depicts the probability of the interventions being cost-effective. At a threshold value of £20,000 per QALY gained, the after-school intervention has the highest probability of being cost-effective (59%) while for the multi-component intervention this probability is at 8%.

Discussion

Main findings

We found that modelling the long-term effects of physical activity among adolescents is feasible, and the model developed here has the potential to estimate the long-term cost-effectiveness of such interventions. The application of the model on two exemplar physical activity interventions in adolescents – one a simple, brief intervention and the other a more complex resource-intensive one – revealed only small differences in terms of lifetime costs per QALYs between the two. Hence, more complex and resource-intensive interventions need not necessarily be better value-for-money in the longer-term compared to cheaper, more targeted approaches. Our findings underline that modelled cost-effectiveness estimates are critically sensitive to assumptions around the sustainability of intervention effects.

For peer review only - http://bmjopen.bmj.com/site/about/guidelines.xhtml

For peer review only

Comparison with previous models

Two previous UK-based modelling studies evaluating the cost-effectiveness of community based physical activity interventions included young people. Although Frew et al[20] used the same basic modelling approach, their model included only three activity categories and study participants included both young people and adults (16-70 year olds). By contrast, our model has four physical activity categories, included two more health states and health effects are modelled over a lifetime (with a time horizon of 65 years). Pringle et al[62] evaluated seven broad categories of community based physical activity interventions (one of which was related to the interventions considered here). Their analysis was based on the NICE/Matrix model[63] which used two activity categories (active or inactive) with 4 disease states. However, they did not focus on young people. Recently, Lee and colleagues[64] modelled the economic and health impact of increasing children's physical activity in the US. However, unlike in the current model, their model specifically looked at the influence of physical activity on weight status and metabolic profiles and ignored decay in intervention effects or the naturally occurring decline in physical activity associated with ageing.

Strengths and limitations

Although our model used a similar modelling framework as previous models,[19,20] we included additional health states and focused on adolescent physical activity interventions – this approach has hitherto been neglected, despite the potential importance of intervening at this key stage. We also include the most up to date available evidence on disease conditions. UK-specific incidence rates were used to ensure that patients entering the model match the likely distribution of events in the UK. We chose not to include sedentary behaviour, as there is ongoing debate around its impact on health independent of physical activity.[65]

As with all models, assumptions were required for the analysis. The model presented here is a simplification of a very complex problem. The baseline age of the cohort is 16 years and the effect of physical activity interventions are likely to differ depending on the population age at baseline. We

For peer review only - http://bmjopen.bmj.com/site/about/guidelines.xhtml

included six disease conditions that have established links with physical (in)activity. This might underestimate the potential impact of physical activity on other disease conditions, most notably mental health. The effect of physical activity on the prevention of depression is still a subject of debate[66], and a clear dose-response relationship between physical activity and reduced depression is not readily apparent.[67] Further empirical evidence is required to facilitate its inclusion in a future iteration of the model. The current model does not allow for transitions between disease states as this requires more complex modelling. However, this may underestimate the potential impact of physical activity. For example, participants with type 2 diabetes tend to have a higher risk of developing cardiovascular conditions.[68] Although the intervention was aimed at adolescents, due to the nature of the disease conditions included in the model, it would mainly be older adults who develop these diseases.

Our assumption on the decay rates of the additional effect of the intervention, which was based on previous modelling studies,[19,69,70] would mean that there would be very little difference in activity between groups at time points when individuals are starting to develop these diseases. We tested different assumptions on the maintenance of intervention effect to examine influence in cost-effectiveness results. Further research into maintenance of intervention effect would provide valuable information. Our analysis focused on physical activity and only considered direct effects that might result from changes in this health behaviour, while holding any other health behaviours constant. In the real world, physical activity would be expected to interact with other health behaviour choices, in ways that might well affect longer term cost and health outcomes. The existing, very sparse, literature on the interaction between different health behaviours suggest a complex and likely context-specific picture.[64,71]

Conclusion

Interventions to promote physical activity among adolescents represent a potentially promising public health measure to reduce the burden of cardiovascular and other non-communicable

For peer review only - http://bmjopen.bmj.com/site/about/guidelines.xhtml

diseases. Faced with limited resources, governments need to carefully weigh the costs of any proposed interventions against the associated health benefits expected to be realised over the longer term, in order to ensure that net health gains are maximised. The model developed here has the potential to assess the long-term, beyond trial duration, value-for-money of such interventions. The two purely illustrative applications of the model convey the notion that complex, resource intensive interventions may not necessarily be the ones considered the better buys compared to cheaper, more targeted ones. Maintaining the effect of any behaviour change interventions is challenging as they require personal commitment, encouragement and support over time.

Author contributions

VG designed the model, performed analysis and wrote the first draft of the manuscript. DT and MS supervised the process and provided input into the interpretation of results. AA and EvS provided critical comments on model structure and data analysis. All authors contributed to the critical revision of the manuscript and approved the final version of the manuscript.

Funding

This report is an independent research commissioned and funded by the Department of Health Policy Research Programme (opportunities within the school environment to shift the distribution of activity intensity in adolescents, PR-R5-0213-25001). The views expressed in this publication are those of the author(s) and not necessarily those of the Department of Health. This work was also supported by the Medical Research Council (unit programme number: MC_UU_12015/7). The work was undertaken under the auspices of the Centre for Diet and Activity Research (CEDAR), a UKCRC Public Health Research Centre of Excellence which is funded by the British Heart Foundation, Cancer Research UK, Economic and Social Research Council, Medical Research Council, the National Institute for Health Research, and the Wellcome Trust (MR/K023187/1).

For peer review only - http://bmjopen.bmj.com/site/about/guidelines.xhtml

Availability of data and material

The model was developed using data from publicly available sources, and all the model inputs are described in the paper.

Competing interests statement

Nothing to report.

Ethics approval and consent to participate

Not applicable.

Acknowledgements

We thank Dr Katie Morton and Dr Kirsten Corder for their comments in the early stage of model development work.

For peer review only

For peer review only - http://bmjopen.bmj.com/site/about/guidelines.xhtml

References

1. Reiner M, Niermann C, Jekauc D *et al.* Long-term health benefits of physical activity--a systematic review of longitudinal studies. *BMC Public Health* 2013; 13: 813.

2. Lee IM, Shiroma EJ, Lobelo F *et al.* Effect of physical inactivity on major non-communicable diseases worldwide: an analysis of burden of disease and life expectancy. *Lancet* 2012; 380: 219-229.

3. Ekelund U, Luan J, Sherar LB *et al.* Moderate to vigorous physical activity and sedentary time and cardiometabolic risk factors in children and adolescents. *JAMA* 2012; 307: 704-712.

4. Biddle SJ, Asare M Physical activity and mental health in children and adolescents: a review of reviews. *Br J Sports Med* 2011; 45: 886-895.

5. Singh A, Uijtdewilligen L, Twisk JW *et al.* Physical activity and performance at school: a systematic review of the literature including a methodological quality assessment. *Arch Pediatr Adolesc Med* 2012; 166: 49-55.

6. Hallal PC, Victora CG, Azevedo MR *et al.* Adolescent Physical Activity and Health. *Sports Medicine* 2006; 36: 1019-1030.

7. Telama R, Yang X, Viikari J *et al.* Physical activity from childhood to adulthood: a 21-year tracking study. *Am J Prev Med* 2005; 28: 267-273.

8. Corder K, Winpenny E, Love R *et al.* Change in physical activity from adolescence to early adulthood: a systematic review and meta-analysis of longitudinal cohort studies. *Br J Sports Med* 2017; doi:10.1136/bjsports-2016-097330.

9. Department of Health. *UK physical activity guidelines*. London: Department of Health, 2011.

10. Warburton DE, Nicol CW, Bredin SS Health benefits of physical activity: the evidence. *CMAJ* 2006; 174: 801-809.

11. Department of Health. *Start active, stay active: a report on physical activity from the four home countries' Chief Medical Officers*. Department of Health, 2011.

For peer review only - http://bmjopen.bmj.com/site/about/guidelines.xhtml

12. Evans K. The inactivity time bomb. The economic cost of physical inactivity in young people: a StreetGames/Cebr report. 2014. [accessed 13 January 2017.] Available from: http://www.streetgames.org/sites/default/files/The-Inactivity-TimeBomb-StreetGames-Cebr-report-April-2014.pdf.

13. Cooper AR, Goodman A, Page AS *et al.* Objectively measured physical activity and sedentary time in youth: the International children's accelerometry database (ICAD). *Int J Behav Nutr Phys Act* 2015; 12: 113.

14. Telama R Tracking of physical activity from childhood to adulthood: a review. *Obes Facts* 2009; 2: 187-195.

15. Sutherland RL, Campbell EM, Lubans DR *et al.* The Physical Activity 4 Everyone Cluster Randomized Trial: 2-Year Outcomes of a School Physical Activity Intervention Among Adolescents. *Am J Prev Med* 2016; 51: 195-205.

16. Grydeland M, Bergh IH, Bjelland M *et al.* Intervention effects on physical activity: the HEIA study - a cluster randomized controlled trial. *Int J Behav Nutr Phys Act* 2013; 10: 17.

17. Borde R, Smith JJ, Sutherland R *et al.* Methodological considerations and impact of school-based interventions on objectively measured physical activity in adolescents: a systematic review and meta-analysis. *Obes Rev* 2017; 18: 476-490.

18. Buxton MJ, Drummond MF, Van Hout BA *et al.* Modelling in economic evaluation: an unavoidable fact of life. *Health Econ* 1997; 6: 217-227.

19. Roux L, Pratt M, Tengs TO *et al.* Cost effectiveness of community-based physical activity interventions. *Am J Prev Med* 2008; 35: 578-588.

20. Frew EJ, Bhatti M, Win K *et al.* Cost-effectiveness of a community-based physical activity programme for adults (Be Active) in the UK: an economic analysis within a natural experiment. *Br J Sports Med* 2014; 48: 207-212.

For peer review only

For peer review only - http://bmjopen.bmj.com/site/about/guidelines.xhtml

21. Wahid A, Manek N, Nichols M *et al.* Quantifying the Association Between Physical Activity and Cardiovascular Disease and Diabetes: A Systematic Review and Meta-Analysis. *J Am Heart Assoc* 2016; 5.

22. Pandey A, Garg S, Khunger M *et al.* Dose-Response Relationship Between Physical Activity and Risk of Heart Failure: A Meta-Analysis. *Circulation* 2015; 132: 1786-1794.

23. Smith AD, Crippa A, Woodcock J *et al.* Physical activity and incident type 2 diabetes mellitus: a systematic review and dose-response meta-analysis of prospective cohort studies. *Diabetologia* 2016.

24. Kyu HH, Bachman VF, Alexander LT *et al.* Physical activity and risk of breast cancer, colon cancer, diabetes, ischemic heart disease, and ischemic stroke events: systematic review and dose-response meta-analysis for the Global Burden of Disease Study 2013. *BMJ* 2016; 354: i3857.

25. Brenner DR, Yannitsos DH, Farris MS *et al.* Leisure-time physical activity and lung cancer risk: A systematic review and meta-analysis. *Lung Cancer* 2016; 95: 17-27.

26. Tardon A, Lee WJ, Delgado-Rodriguez M *et al.* Leisure-time physical activity and lung cancer: a meta-analysis. *Cancer Causes Control* 2005; 16: 389-397.

27. Allender S, Foster C, Scarborough P *et al.* The burden of physical activity-related ill health in the UK. *J Epidemiol Community Health* 2007; 61: 344-348.

28. National Institute for Health and Care Excellence. *Guide to the methods of technology appraisal 2013*. London: National Institute for Health and Care Excellence, 2013.

29. Office for National Statistics. National Life Tables: United Kingdom. 2016. [accessed 3 November 2016.] Available from: http://www.ons.gov.uk/file?uri=/peoplepopulationandcommunity/birthsdeathsandmarriages/lifeexpectancies/datasets/nationallifetablesenglandreferencetables/current/nlteng1315reg.xls.

30. Naimark DM, Bott M, Krahn M The half-cycle correction explained: two alternative pedagogical approaches. *Med Decis Making* 2008; 28: 706-712.

For peer review only - http://bmjopen.bmj.com/site/about/guidelines.xhtml

31. Office for National Statistics. MYE1: Population Estimates Summary for the UK, mid-2014. 2016. [accessed 20 October 2016.] Available from: https://www.ons.gov.uk/file?uri=/peoplepopulationandcommunity/populationandmigration/populationestimates/datasets/populationestimatesanalysistool/mid2014/analysistoolmid2014uk.zip.

32. Scholes S, Mindell J (2013) *Physical activity in children*. In: Craig R, Mindell J, editors. Health Survey for England 2012.(2013) London: The Health and Social Care Information Centre

33. Sutcliffe SJ, Fox KF, Wood DA *et al.* Incidence of coronary heart disease in a health authority in London: review of a community register. *BMJ* 2003; 326: 20.

34. Bamford J, Sandercock P, Dennis M *et al.* A prospective study of acute cerebrovascular disease in the community: the Oxfordshire Community Stroke Project 1981-86. 1. Methodology, demography and incident cases of first-ever stroke. *J Neurol Neurosurg Psychiatry* 1988; 51: 1373-1380.

35. Cowie MR, Wood DA, Coats AJ *et al.* Incidence and aetiology of heart failure; a population-based study. *Eur Heart J* 1999; 20: 421-428.

36. Sharma M, Nazareth I, Petersen I Trends in incidence, prevalence and prescribing in type 2 diabetes mellitus between 2000 and 2013 in primary care: a retrospective cohort study. *BMJ Open* 2016; 6: e010210.

37. Cancer Research UK. Lung cancer incidence. 2016. [accessed 26 October 2016.] Available from: http://www.cancerresearchuk.org/sites/default/files/cstream-node/cases_crude_lung_2.xls.

38. Cancer Research UK. Bowel cancer incidence. 2016. [accessed 26 October 2016.] Available from: http://www.cancerresearchuk.org/sites/default/files/cstream-node/cases_crude_bowel_2.xls.

39. Cancer Research UK. Breast cancer statistics. 2016. [accessed 26 October 2016.] Available from: http://www.cancerresearchuk.org/sites/default/files/cstream-node/cases_crude_f_breast_2.xls.

For peer review only

For peer review only - http://bmjopen.bmj.com/site/about/guidelines.xhtml

40. Hurley SF, Matthews JP The Quit Benefits Model: a Markov model for assessing the health benefits and health care cost savings of quitting smoking. *Cost Eff Resour Alloc* 2007; 5: 2.

41. Smolina K, Wright FL, Rayner M *et al.* Determinants of the decline in mortality from acute myocardial infarction in England between 2002 and 2010: linked national database study. *BMJ* 2012; 344: d8059.

42. Bamford J, Sandercock P, Dennis M *et al.* A prospective study of acute cerebrovascular disease in the community: the Oxfordshire Community Stroke Project--1981-86. 2. Incidence, case fatality rates and overall outcome at one year of cerebral infarction, primary intracerebral and subarachnoid haemorrhage. *J Neurol Neurosurg Psychiatry* 1990; 53: 16-22.

43. MacIntyre K, Capewell S, Stewart S *et al.* Evidence of improving prognosis in heart failure: trends in case fatality in 66 547 patients hospitalized between 1986 and 1995. *Circulation* 2000; 102: 1126-1131.

44. Seshasai SR, Kaptoge S, Thompson A *et al.* Diabetes mellitus, fasting glucose, and risk of cause-specific death. *N Engl J Med* 2011; 364: 829-841.

45. Bronnum-Hansen H, Davidsen M, Thorvaldsen P *et al.* Long-term survival and causes of death after stroke. *Stroke* 2001; 32: 2131-2136.

46. Office for National Statistics. Cancer survival in England - adults diagnosed. 2016. [accessed 26 October 2016.] Available from: http://www.ons.gov.uk/file?uri=/peoplepopulationandcommunity/healthandsocialcare/conditionsanddiseases/datasets/cancersurvivalratescancersurvivalinenglandadultsdiagnosed/20102014/finalreferencetablev2.xls.

47. Mears R, Jago R Effectiveness of after-school interventions at increasing moderate-to-vigorous physical activity levels in 5- to 18-year olds: a systematic review and meta-analysis. *Br J Sports Med* 2016; 50: 1315-1324.

48. Ward S, Lloyd Jones M, Pandor A *et al.* A systematic review and economic evaluation of statins for the prevention of coronary events. *Health Technol Assess* 2007; 11: 1-160, iii-iv.

For peer review only - http://bmjopen.bmj.com/site/about/guidelines.xhtml

49. Clarke P, Gray A, Legood R *et al.* The impact of diabetes-related complications on healthcare costs: results from the United Kingdom Prospective Diabetes Study (UKPDS Study No. 65). *Diabet Med* 2003; 20: 442-450.

50. Farmer AJ, Wade AN, French DP *et al.* Blood glucose self-monitoring in type 2 diabetes: a randomised controlled trial. *Health Technol Assess* 2009; 13: iii-iv, ix-xi, 1-50.

51. Madan J, Rawdin A, Stevenson M *et al.* A rapid-response economic evaluation of the UK NHS Cancer Reform Strategy breast cancer screening program extension via a plausible bounds approach. *Value Health* 2010; 13: 215-221.

52. Tappenden P, Chilcott J, Eggington S *et al.* Option appraisal of population-based colorectal cancer screening programmes in England. *Gut* 2007; 56: 677-684.

53. Curtis L, Burns A, editors (2015) Unit Costs of Health and Social Care 2015. Canterbury: Personal Social Services Research Unit, University of Kent.

54. Sullivan PW, Slejko JF, Sculpher MJ *et al.* Catalogue of EQ-5D scores for the United Kingdom. *Med Decis Making* 2011; 31: 800-804.

55. Craig R, Mindell J (2013) *Health Survey for England 2012*. In: Craig R, Mindell J, editors.(2013) London: The Health and Social Care Information Centre

56. Jago R, Sebire SJ, Davies B *et al.* Randomised feasibility trial of a teaching assistant led extracurricular physical activity intervention for 9 to 11 year olds: Action 3:30. *Int J Behav Nutr Phys Act* 2014; 11: 114.

57. Sutherland R, Reeves P, Campbell E *et al.* Cost effectiveness of a multi-component school-based physical activity intervention targeting adolescents: the 'Physical Activity 4 Everyone' cluster randomized trial. *Int J Behav Nutr Phys Act* 2016; 13: 94.

58. The Campbell and Cochrane Economics Methods Group (CCEMG) and the Evidence for Policy and Practice Information and Coordinating Centre (EPPI-Centre). CCEMG - EPPI-Centre Cost Converter (v1.5). [accessed 31 January 2017.] Available from: http://eppi.ioe.ac.uk/costconversion/default.aspx.

For peer review only - http://bmjopen.bmj.com/site/about/guidelines.xhtml

59. Siebert U, Alagoz O, Bayoumi AM *et al.* State-transition modeling: a report of the ISPOR-SMDM Modeling Good Research Practices Task Force--3. *Value Health* 2012; 15: 812-820.

60. Fenwick E, Marshall DA, Levy AR *et al.* Using and interpreting cost-effectiveness acceptability curves: an example using data from a trial of management strategies for atrial fibrillation. *BMC Health Serv Res* 2006; 6: 52.

61. Briggs A, Claxton K, Sculpher MJ (2006) *Chapter 4: Making decision models probabilistic*. Decision Modelling for Health Economic Evaluation.(2006): Oxford University Press. pp. 77-120.

62. Pringle A, Cooke C, Gilson N *et al.* Cost-effectiveness of interventions to improve moderate physical activity: A study in nine UK sites. *Health Education Journal* 2010; 69: 211-224.

63. Matrix Research and Consulting. *Modeling the cost effectiveness of physical activity interventions*. London: National Institute for Health and Clinical Excellence, 2006.

64. Lee BY, Adam A, Zenkov E *et al.* Modeling The Economic And Health Impact Of Increasing Children's Physical Activity In The United States. *Health Aff (Millwood)* 2017; 36: 902-908.

65. Pearson N, Braithwaite RE, Biddle SJ *et al.* Associations between sedentary behaviour and physical activity in children and adolescents: a meta-analysis. *Obes Rev* 2014; 15: 666-675.

66. Teychenne M, Ball K, Salmon J Physical activity and likelihood of depression in adults: a review. *Prev Med* 2008; 46: 397-411.

67. Mammen G, Faulkner G Physical activity and the prevention of depression: a systematic review of prospective studies. *Am J Prev Med* 2013; 45: 649-657.

68. Shah AD, Langenberg C, Rapsomaniki E *et al.* Type 2 diabetes and incidence of a wide range of cardiovascular diseases: a cohort study in 1.9 million people. *Lancet* 2015; 385 Suppl 1: S86.

69. Cobiac LJ, Vos T, Barendregt JJ Cost-effectiveness of interventions to promote physical activity: a modelling study. *PLoS Med* 2009; 6: e1000110.

70. Gc VS, Suhrcke M, Hardeman W *et al.* Cost-Effectiveness and Value of Information Analysis of Brief Interventions to Promote Physical Activity in Primary Care. *Value Health* 2018; 21: 18-26.

For peer review only - http://bmjopen.bmj.com/site/about/guidelines.xhtml

71. Böckerman P, Hyytinen A, Kaprio J *et al.* If you drink, don't smoke: Joint associations between risky health behaviors and labor market outcomes. *Soc Sci Med* 2018; 207: 55-63.

For peer review only

For peer review only - http://bmjopen.bmj.com/site/about/guidelines.xhtml

Figure title and legend

Figure 1: Conceptual overview of the model

Figure 2: Scatter plot of incremental costs and QALYs for each intervention, relative to no intervention

Figure 3: Cost-effectiveness acceptability curves

For peer review only

For peer review only - http://bmjopen.bmj.com/site/about/guidelines.xhtml

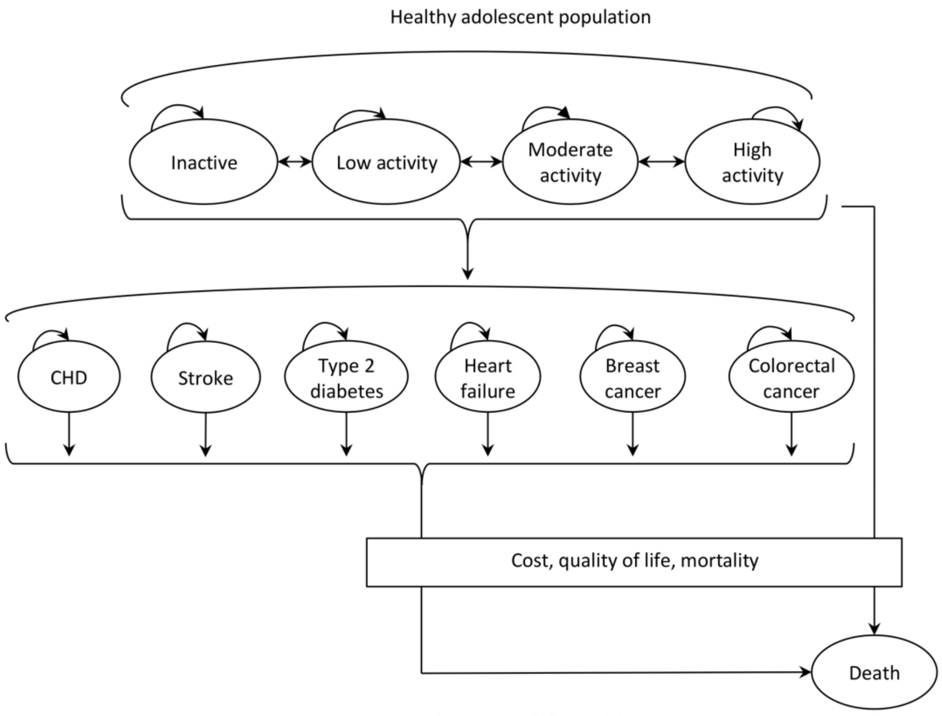

Conceptual overview of the model

168x125mm (300 x 300 DPI)

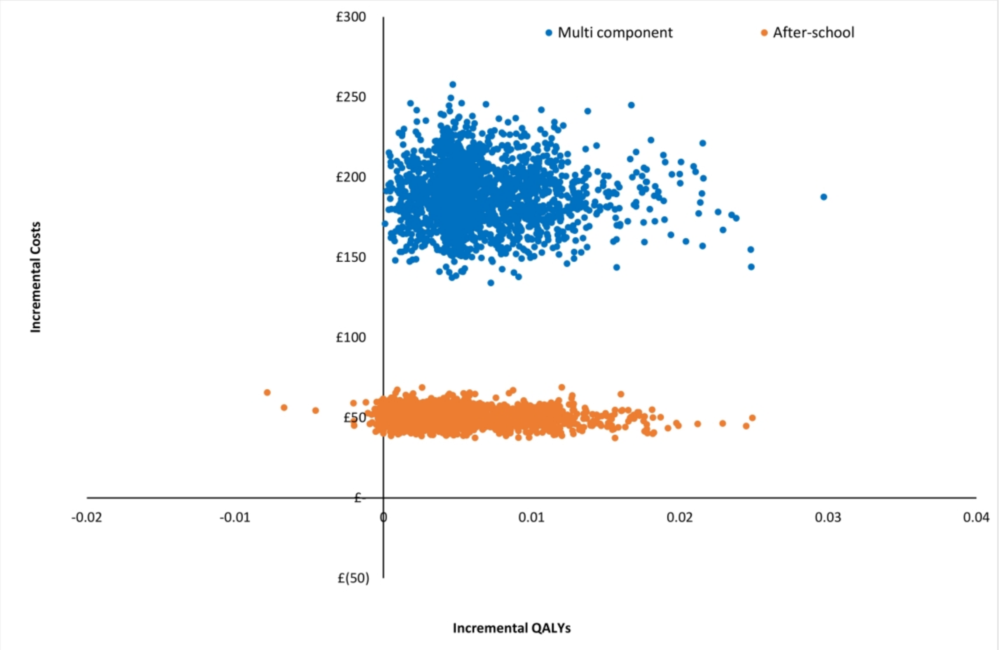

Scatter plot of incremental costs and QALYs for each intervention, relative to no intervention

259x168mm (300 x 300 DPI)

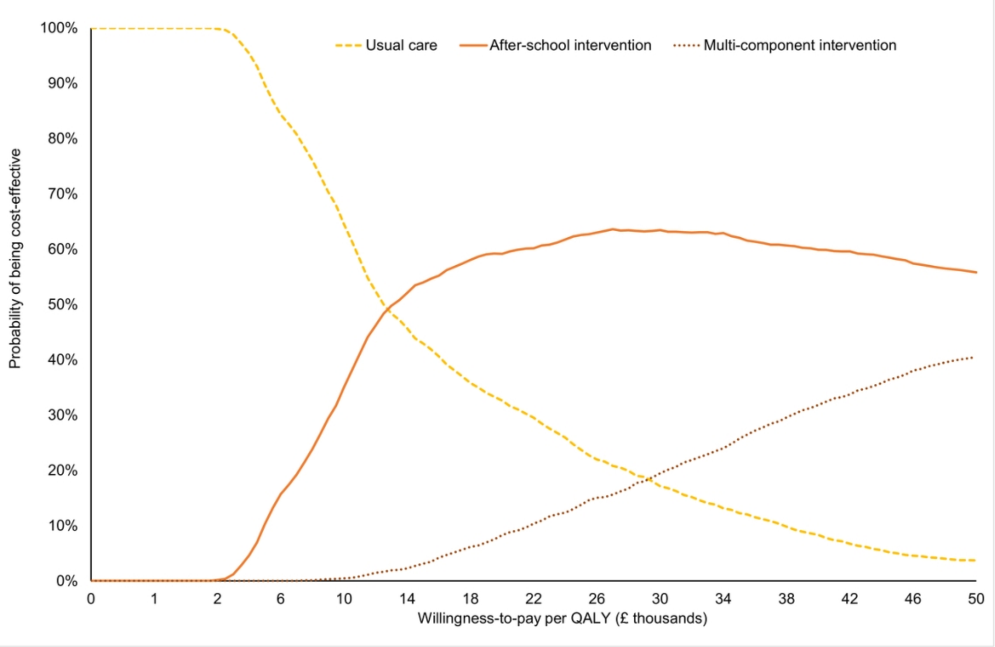

Cost-effectiveness acceptability curves

111x72mm (300 x 300 DPI)

CHEERS statement

Section/Item	Item no.	Recommendation	Reported on page no./line no.
**Title and abstract**			
Title	1	Identify the study as an economic evaluation, or use more specific terms such as ''cost-effectiveness analysis'' and describe the interventions compared.	1
Abstract	2	Provide a structured summary of objectives, perspective, setting, methods (including study design and inputs), results (including base-case and uncertainty analyses), and conclusions.	2
**Introduction**			
Background and objectives	3	Provide an explicit statement of the broader context for the study. Present the study question and its relevance for health policy or practice decisions.	4-5
**Methods**			
Target population and subgroups	4	Describe characteristics of the base-case population and subgroups analysed including why they were chosen.	5
Setting and location	5	State relevant aspects of the system(s) in which the decision(s) need(s) to be made	5
Study perspective	6	Describe the perspective of the study and relate this to the costs being evaluated.	13
Comparators	7	Describe the interventions or strategies being compared and state why they were chosen.	8-9
Time horizon	8	State the time horizon(s) over which costs and consequences are being evaluated and say why appropriate.	6
Discount rate	9	Report the choice of discount rate(s) used for costs and outcomes and say why appropriate	6
Choice of health outcomes	10	Describe what outcomes were used as the measure(s) of benefit in the evaluation and their relevance for the type of analysis performed	9-11
Measurement of effectiveness	11a	Single study–based estimates: Describe fully the design features of the single effectiveness study and why the single study was a sufficient source of clinical effectiveness data.	8-9
	11b	Synthesis-based estimates: Describe fully the methods used for the identification of included studies and synthesis of clinical effectiveness data.	8-9
Measurement and valuation of preference-based outcomes	12	If applicable, describe the population and methods used to elicit preferences for outcomes.	N/A
Estimating resources and costs	13a	Single study–based economic evaluation: Describe approaches used to estimate resource use associated with the alternative interventions. Describe primary or secondary research methods for valuing each resource item in terms of its unit cost. Describe any adjustments made to approximate to opportunity costs.	
	13b	Model-based economic evaluation: Describe approaches and data sources used to estimate resource use associated with model health states. Describe primary or secondary research methods for valuing each resource item in terms of its unit cost. Describe any adjustments made to approximate to opportunity costs.	9-10, Table 1
Currency, price date, and conversion	14	Report the dates of the estimated resource quantities and unit costs. Describe methods for adjusting estimated unit costs to the year of reported costs if necessary. Describe methods for converting costs into a common currency base and the exchange rate.	9

For peer review only - http://bmjopen.bmj.com/site/about/guidelines.xhtml

Section/Item	Item no.	Recommendation	Reported on page no./line no.
Choice of model	15	Describe and give reasons for the specific type of decision-analytic model used. Providing a figure to show model structure is strongly recommended.	5
Assumptions	16	Describe all structural or other assumptions underpinning the decision-analytic model.	5-9 & 11-12
Analytic methods	17	Describe all analytic methods supporting the evaluation. This could include methods for dealing with skewed, missing, or censored data; extrapolation methods; methods for pooling data; approaches to validate or make adjustments (e.g., half-cycle corrections) to a model; and methods for handling population heterogeneity and uncertainty.	5, 11-14
**Results**			
Study parameters	18	Report the values, ranges, references, and if used, probability distributions for all parameters. Report reasons or sources for distributions used to represent uncertainty where appropriate. Providing a table to show the input values is strongly recommended.	Table 1-2, Supplementary Table S1, S2
Incremental costs and outcomes	19	For each intervention, report mean values for the main categories of estimated costs and outcomes of interest, as well as mean differences between the comparator groups. If applicable, report incremental cost-effectiveness ratios.	14-15
Characterizing uncertainty	20a	Single study–based economic evaluation: Describe the effects of sampling uncertainty for estimated incremental cost, incremental effectiveness, and incremental cost-effectiveness, together with the impact of methodological assumptions (such as discount rate, study perspective).	N/A
	20b	Model-based economic evaluation: Describe the effects on the results of uncertainty for all input parameters, and uncertainty related to the structure of the model and assumptions.	15-16
Characterizing heterogeneity	21	If applicable, report differences in costs, outcomes, or cost-effectiveness that can be explained by variations between subgroups of patients with different baseline characteristics or other observed variability in effects that are not reducible by more information.	N/A
**Discussion**			
Study findings, limitations, generalizability, and current knowledge	22	Summarize key study findings and describe how they support the conclusions reached. Discuss limitations and the generalizability of the findings and how the findings fit with current knowledge.	16-18
**Other**			
Source of funding	23	Describe how the study was funded and the role of the funder in the identification, design, conduct, and reporting of the analysis. Describe other nonmonetary sources of support.	19
Conflicts of interest	24	Describe any potential for conflict of interest among study contributors in accordance with journal policy. In the absence of a journal policy, we recommend authors comply with International Committee of Medical Journal Editors' recommendations.	19

For consistency, the CHEERS statement checklist format is based on the format of the CONSORT statement checklist

For peer review only - http://bmjopen.bmj.com/site/about/guidelines.xhtml

Online Supplemental File

A. Model input parameters

Table S1: Physical activity at baseline and estimates of disease incidence

Parameters	Male				Female				Distribution	Source
	Inactive	Low	Moderate	High	Inactive	Low	Moderate	High		
Baseline PA levels	12%	7%	25%	56%	23%	35%	20%	22%	Fixed	[1]
Relative risk (95% CI) estimates used to adjust disease incidence										
CHD	1.0	0.87 (0.80–0.95)	0.78 (0.74–0.82)	0.70 (0.66–0.75)	1.0	0.87 (0.80–0.95)	0.78 (0.80–0.95)	0.70 (0.80–0.95)	lognormal	[2]
Stroke	1.0	0.85 (0.80–0.91)	0.81 (0.74–0.88)	0.76 (0.68–0.85)	1.0	0.85 (0.80–0.91)	0.81 (0.74–0.88)	0.76 (0.68–0.85)	lognormal	[2]
Heart failure	1.0	0.85 (0.79–0.92)	0.78 (0.75–0.82)	0.70 (0.67–0.73)	1.0	0.85 (0.79–0.92)	0.78 (0.75–0.82)	0.70 (0.67–0.73)	lognormal	[3]
Type 2 diabetes	1.0	0.93 (0.92–0.95)	0.75 (0.69–0.80)	0.60 (0.51–0.70)	1.0	0.93 (0.92–0.95)	0.75 (0.69–0.80)	0.60 (0.51–0.70)	lognormal	[4]
Breast cancer	-	-	-	-	1.0	0.97 (0.94–0.998)	0.94 (0.90–0.98)	0.86 (0.83–0.90)	lognormal	[5]
Colorectal cancer	1.0	0.90 (0.85–0.95)	0.83 (0.77–0.97)	0.79 (0.74–0.85)	1.0	0.90 (0.85–0.95)	0.83 (0.77–0.97)	0.79 (0.74–0.85)	lognormal	[5]

For peer review only - http://bmjopen.bmj.com/site/about/guidelines.xhtml
For peer review only

Parameters	Male				Female				Distribution	Source
	Inactive	Low	Moderate	High	Inactive	Low	Moderate	High		
CHD incidence										[6]
<35	<0.01%	<0.01%	<0.01%	<0.01%	<0.01%	<0.01%	<0.01%	<0.01%	beta	
35-44	0.01%	0.00%	<0.01%	<0.01%	<0.01%	<0.01%	<0.01%	<0.01%	beta	
45-54	0.10%	0.09%	0.08%	0.07%	0.02%	0.01%	0.01%	0.01%	beta	
55-64	0.41%	0.36%	0.32%	0.29%	0.11%	0.10%	0.09%	0.08%	beta	
65-74	1.08%	0.94%	0.84%	0.76%	0.44%	0.38%	0.34%	0.31%	beta	
75+	1.67%	1.45%	1.30%	1.17%	0.67%	0.59%	0.53%	0.47%	beta	
Stroke incidence										[7]
<35	<0.01%	<0.01%	<0.01%	<0.01%	<0.01%	<0.01%	<0.01%	<0.01%	beta	
35-44	<0.01%	<0.01%	<0.01%	<0.01%	0.01%	0.01%	0.01%	0.01%	beta	
45-54	0.01%	0.01%	<0.01%	<0.01%	0.01%	0.01%	0.01%	0.01%	beta	
55-64	0.03%	0.03%	0.03%	0.02%	0.02%	0.02%	0.02%	0.02%	beta	
65-74	0.08%	0.07%	0.07%	0.06%	0.05%	0.05%	0.04%	0.04%	beta	
75-84	0.41%	0.35%	0.33%	0.31%	0.28%	0.23%	0.22%	0.21%	beta	
Heart failure incidence										[8]
<35	<0.01%	<0.01%	<0.01%	<0.01%	<0.01%	<0.01%	<0.01%	<0.01%	beta	
35-44	0.03%	0.02%	0.02%	0.02%	0.03%	0.02%	0.02%	0.02%	beta	
45-54	0.04%	0.03%	0.03%	0.03%	0.01%	0.01%	0.01%	0.01%	beta	
55-64	0.21%	0.18%	0.16%	0.14%	0.08%	0.07%	0.07%	0.06%	beta	
65-74	0.48%	0.41%	0.37%	0.33%	0.28%	0.24%	0.22%	0.19%	beta	
75-84	1.14%	0.97%	0.89%	0.80%	0.64%	0.55%	0.50%	0.45%	beta	

For peer review only - http://bmjopen.bmj.com/site/about/guidelines.xhtml

Parameters	Male				Female				Distribution	Source
	Inactive	Low	Moderate	High	Inactive	Low	Moderate	High		
Type 2 diabetes incidence										[9]
<20	0.01%	0.01%	0.01%	0.01%	0.03%	0.03%	0.03%	0.03%	beta	
20–29	0.05%	0.04%	0.04%	0.03%	0.14%	0.12%	0.11%	0.10%	beta	
30–39	0.17%	0.15%	0.13%	0.13%	0.24%	0.20%	0.18%	0.17%	beta	
40–49	0.51%	0.44%	0.38%	0.37%	0.38%	0.32%	0.28%	0.27%	beta	
50–59	0.99%	0.85%	0.74%	0.71%	0.67%	0.57%	0.50%	0.48%	beta	
60–69	1.43%	1.23%	1.07%	1.03%	1.02%	0.88%	0.76%	0.74%	beta	
70–79	1.53%	1.31%	1.15%	1.11%	1.23%	1.06%	0.92%	0.89%	beta	
80–89	1.05%	0.90%	0.79%	0.76%	0.87%	0.74%	0.65%	0.63%	beta	
Breast cancer incidence										[10]
<25					<0.01%	<0.01%	<0.01%	<0.01%	beta	
25 to 29					0.01%	0.01%	0.01%	0.01%	beta	
30 to 34					0.03%	0.03%	0.03%	0.02%	beta	
35 to 39					0.07%	0.07%	0.06%	0.05%	beta	
40 to 44					0.13%	0.13%	0.12%	0.10%	beta	
45 to 49					0.24%	0.23%	0.21%	0.19%	beta	
50 to 54					0.30%	0.29%	0.27%	0.23%	beta	
55 to 59					0.29%	0.28%	0.27%	0.23%	beta	
60 to 64					0.38%	0.36%	0.34%	0.29%	beta	
65 to 69					0.44%	0.42%	0.40%	0.34%	beta	
70 to 74					0.37%	0.35%	0.33%	0.29%	beta	
75 to 79					0.40%	0.39%	0.37%	0.32%	beta	
80 to 84					0.44%	0.42%	0.40%	0.34%	beta	

For peer review only

Parameters	Male				Female				Distribution	Source
	Inactive	Low	Moderate	High	Inactive	Low	Moderate	High		
Colorectal cancer incidence										[11]
<30	<0.01%	<0.01%	<0.01%	<0.01%	<0.01%	<0.01%	<0.01%	<0.01%	beta	
30 to 34	0.01%	0.01%	0.01%	0.01%	0.01%	0.01%	0.01%	0.01%	beta	
35 to 39	0.01%	0.01%	0.01%	0.01%	0.01%	0.01%	0.01%	0.01%	beta	
40 to 44	0.01%	0.01%	0.01%	0.01%	0.01%	0.01%	0.01%	0.01%	beta	
45 to 49	0.03%	0.03%	0.02%	0.02%	0.02%	0.02%	0.02%	0.02%	beta	
50 to 54	0.06%	0.05%	0.05%	0.05%	0.04%	0.04%	0.04%	0.03%	beta	
55 to 59	0.10%	0.09%	0.08%	0.08%	0.07%	0.06%	0.06%	0.06%	beta	
60 to 64	0.19%	0.17%	0.15%	0.15%	0.11%	0.10%	0.09%	0.09%	beta	
65 to 69	0.26%	0.23%	0.21%	0.20%	0.15%	0.13%	0.12%	0.12%	beta	
70 to 74	0.37%	0.34%	0.31%	0.29%	0.22%	0.20%	0.18%	0.17%	beta	
75 to 79	0.44%	0.40%	0.37%	0.35%	0.26%	0.24%	0.22%	0.21%	beta	
80 to 84	0.54%	0.49%	0.45%	0.43%	0.34%	0.31%	0.28%	0.27%	beta	

Inactive: <30; Low: 30-149; Moderate: 150-420 and High: ≥421 MVPA min per week

For peer review only

For peer review only - http://bmjopen.bmj.com/site/about/guidelines.xhtml

Table S2: Estimates of mortality parameters

Parameter	Value				Distribution	Source
	Male Mean	Male SE	Female mean	Female SE		
CHD case fatality rate						[6]
<54 years	0.129	0.013	0.1245	0.0125	beta	
55-64 years	0.132	0.013	0.1597	0.0160	beta	
65-74 years	0.177	0.018	0.2235	0.0224	beta	
75-84 years	0.244	0.024	0.3009	0.0301	beta	
85+ years	0.315	0.032	0.3668	0.0367	beta	
Stroke case fatality rate	0.1730	0.0152	0.1730	0.0152	beta	[12]
Heart failure case fatality rate						[13]
<35 years	0.0000	0.0000	0.0000	0.0000	beta	
35-55 years	0.0989	0.0050	0.0306	0.0001	beta	
55-64 years	0.1253	0.0037	0.1094	0.0001	beta	
65-74 years	0.1666	0.0028	0.1197	0.0001	beta	
75-84 years	0.1989	0.0027	0.1262	0.0001	beta	
85+ years	0.2279	0.0041	0.1240	0.0001	beta	
Death rate after 1st nonfatal CHD						[14]
<45 years	0.0130	0.0020	0.0130	0.0050	beta	
45-54 years	0.0170	0.0010	0.0260	0.0030	beta	
55-64 years	0.0380	0.0020	0.0350	0.0020	beta	
65-74 years	0.0830	0.0020	0.0820	0.0030	beta	
75-84 years	0.1430	0.0040	0.1590	0.0040	beta	
85+ years	0.2620	0.0110	0.2660	0.0080	beta	
SMR for patients after a 1st nonfatal stroke	2.40	0.08	2.21	0.10	Lognormal	[15]
Death rate after 1st nonfatal heart failure						[13]
<55 years	0.0306	0.0001	0.0306	0.0001	beta	
55-64 years	0.1094	0.0001	0.1094	0.0001	beta	
65-74 years	0.1197	0.0001	0.1197	0.0001	beta	
75-84 years	0.1262	0.0001	0.1262	0.0001	beta	
85+ years	0.1240	0.0001	0.1240	0.0001	beta	
HR for death among patients with type 2 diabetes	1.80	0.0269	1.80	0.0269	lognormal	[16]
Breast cancer mortality	–	–	0.0290	0.0004	beta	[17]
Colorectal cancer mortality	0.1038	0.0006	0.1035	0.0006	beta	[17]
Lung cancer mortality	0.3523	0.0004	0.3112	0.0005	beta	[17]

HR = Hazard ratio; SMR = Standardised mortality ratio

For peer review only - http://bmjopen.bmj.com/site/about/guidelines.xhtml

B. Model validation results

1. Cohort profile

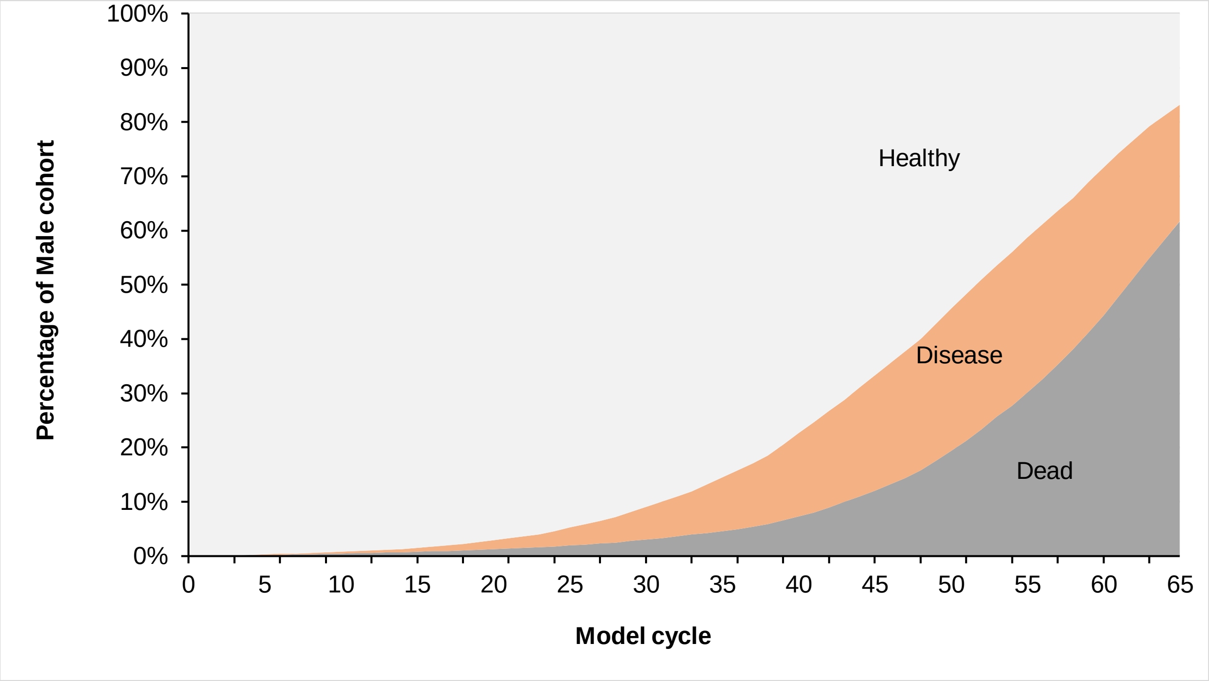

Figure S1: Cohort profile (male)

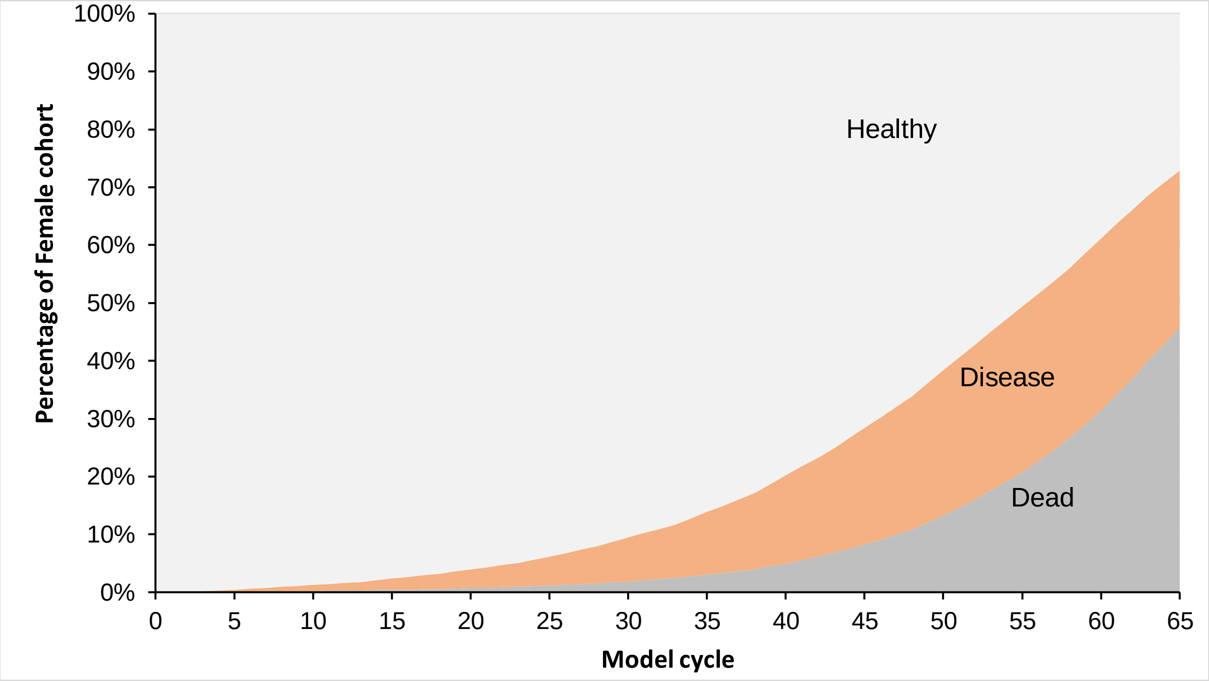

Figure S2: Cohort profile (female)

For peer review only - http://bmjopen.bmj.com/site/about/guidelines.xhtml

2. Prevalence of physical activity levels by age group and gender

Figure S3: Physical activity levels by age group in Male (HSE 2012 data vs no intervention 'control')

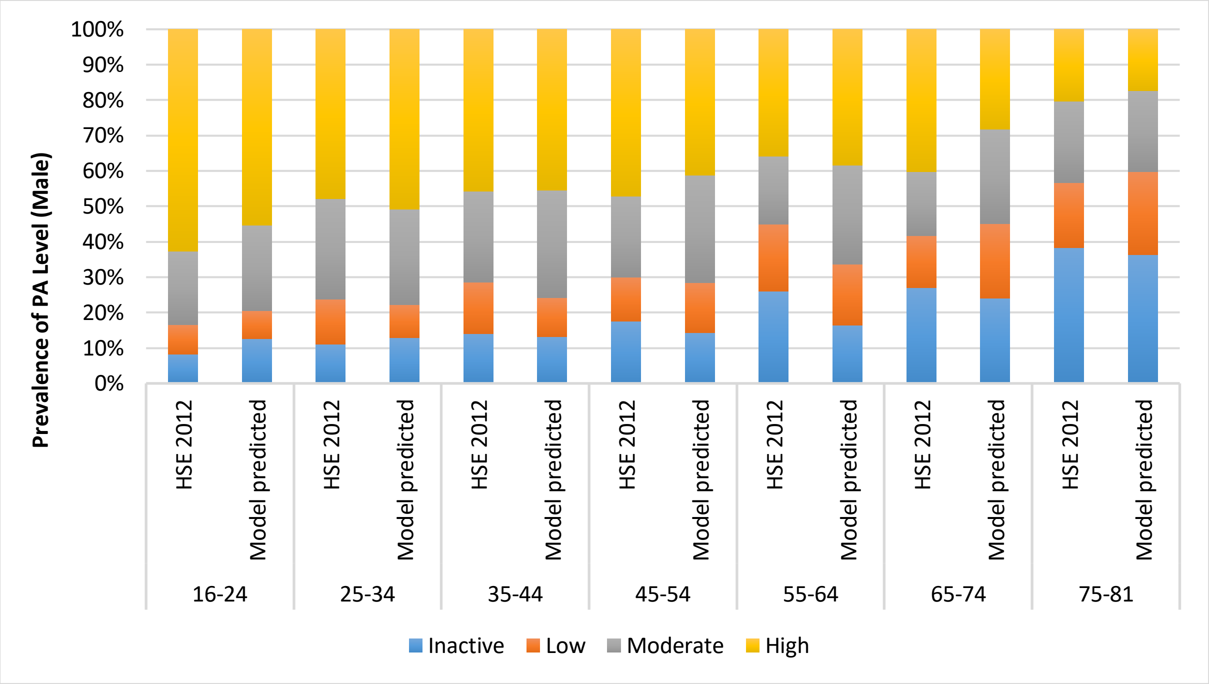

Figure S4: Physical activity levels by age group in Female (HSE 2012 data vs no intervention 'control')

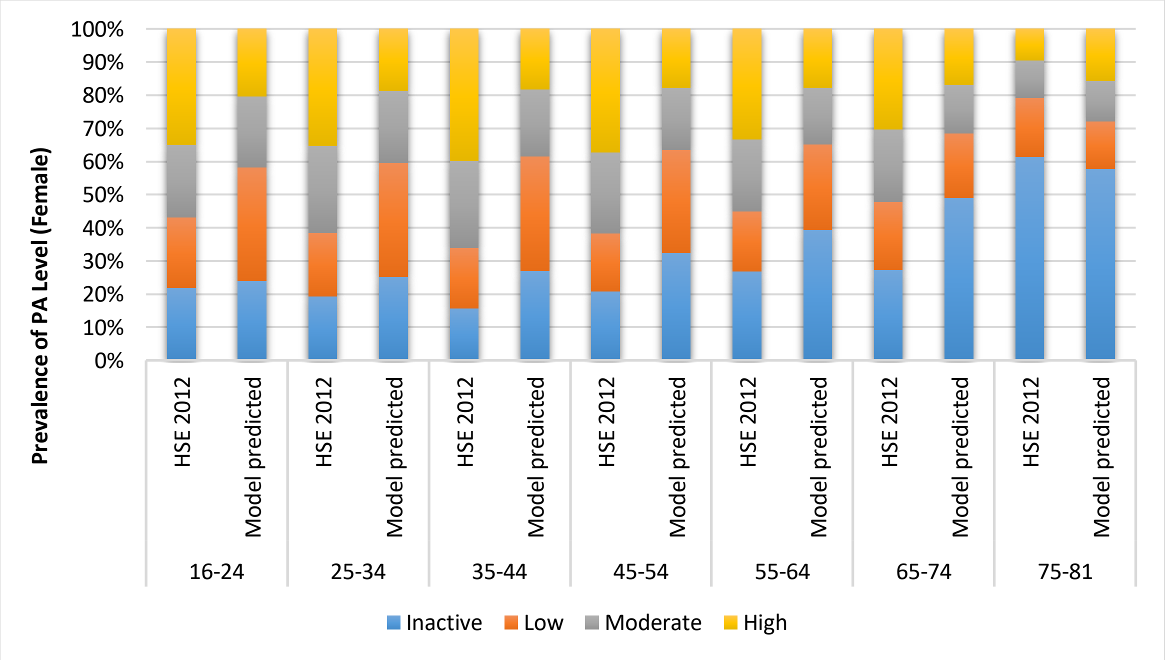

For peer review only - http://bmjopen.bmj.com/site/about/guidelines.xhtml

3. Risk of disease events

Figure S5: CHD incidence: model predicted versus data informing model parameter by age-group and gender

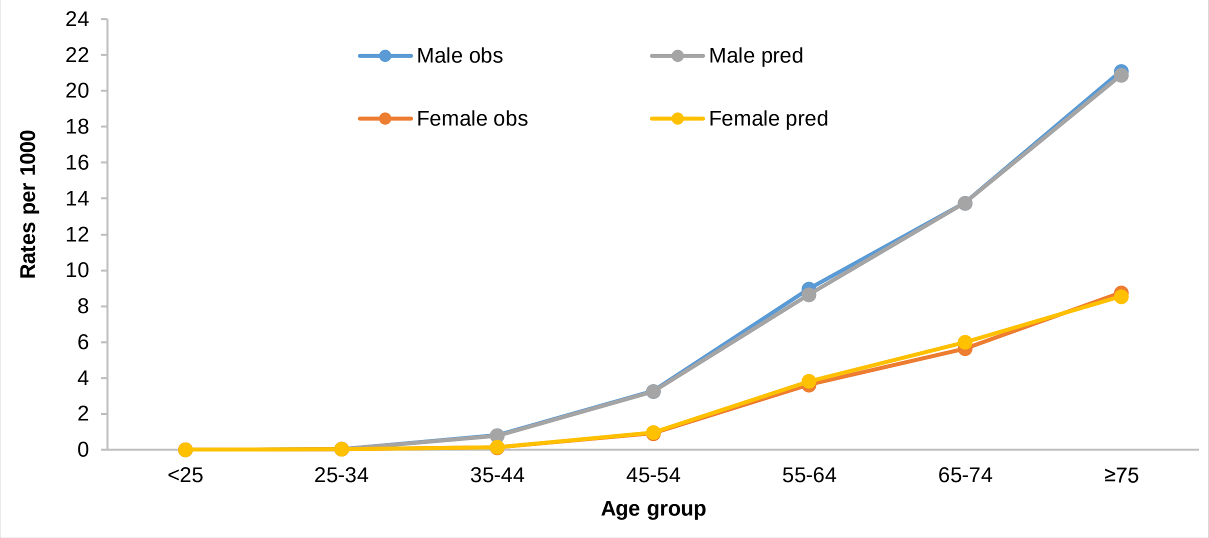

Figure S6: Stroke incidence: model predicted versus data informing model parameter by age-group and gender

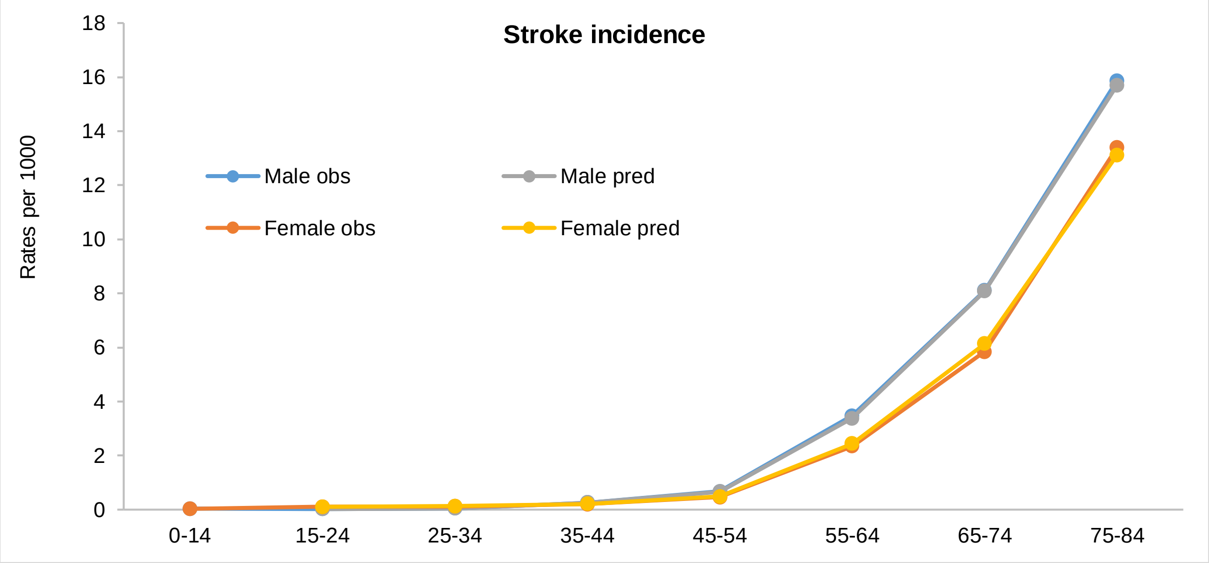

For peer review only - http://bmjopen.bmj.com/site/about/guidelines.xhtml

Figure S7: Heart failure incidence: model predicted versus data informing model parameter by age-group and gender

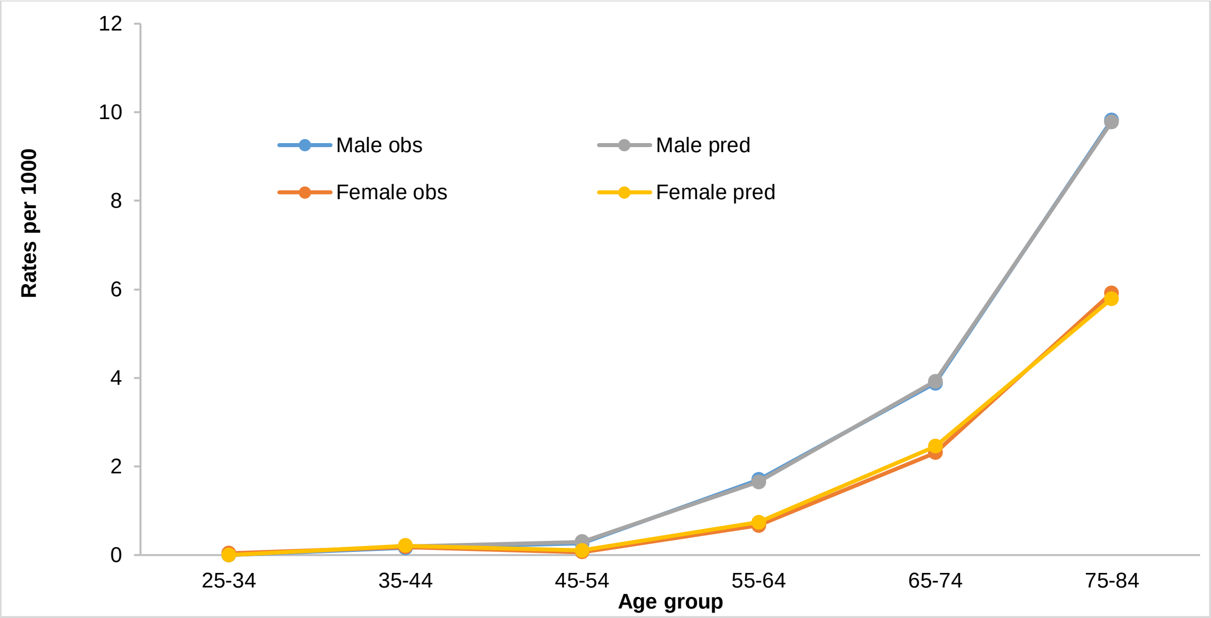

Figure S8: Incidence of type 2 diabetes: model predicted versus data informing model parameter by age-group and gender

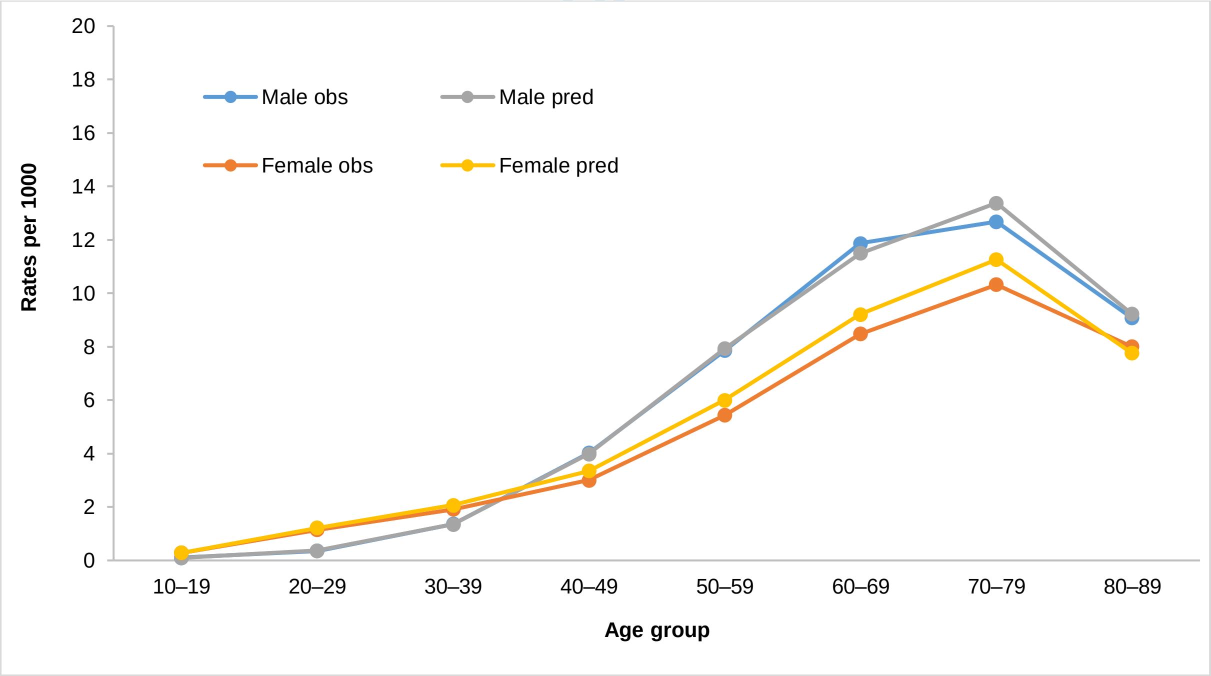

For peer review only - http://bmjopen.bmj.com/site/about/guidelines.xhtml

Figure S9: Breast cancer (female) incidence: model predicted versus data informing model parameter by age-group and gender

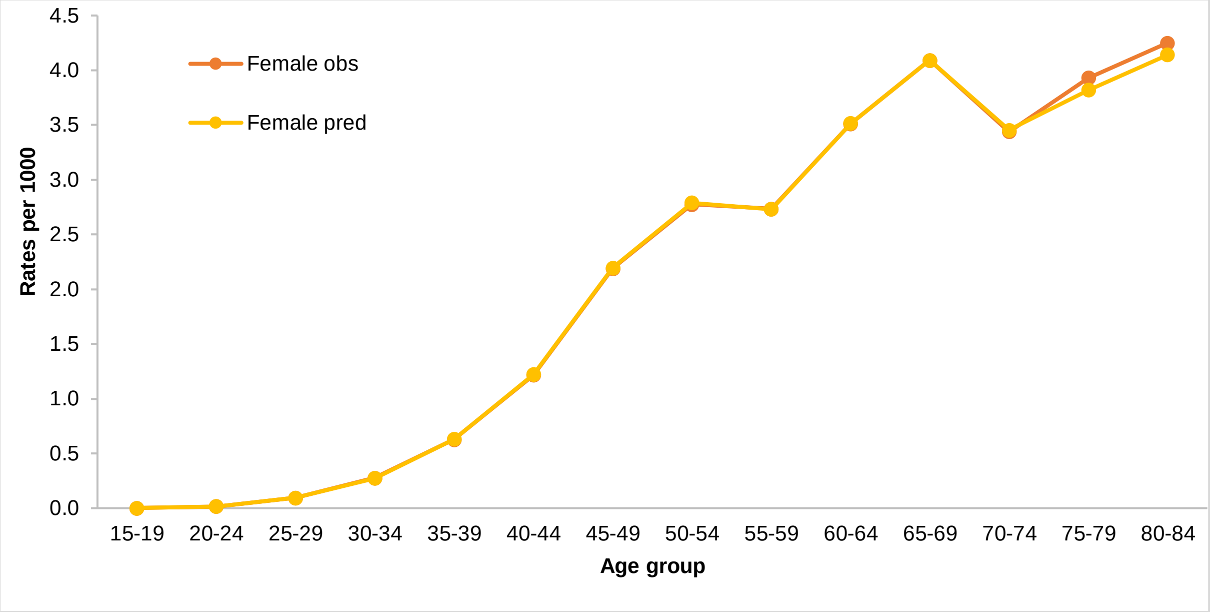

Figure S10: Colorectal cancer incidence: model predicted versus data informing model parameter by age-group and gender

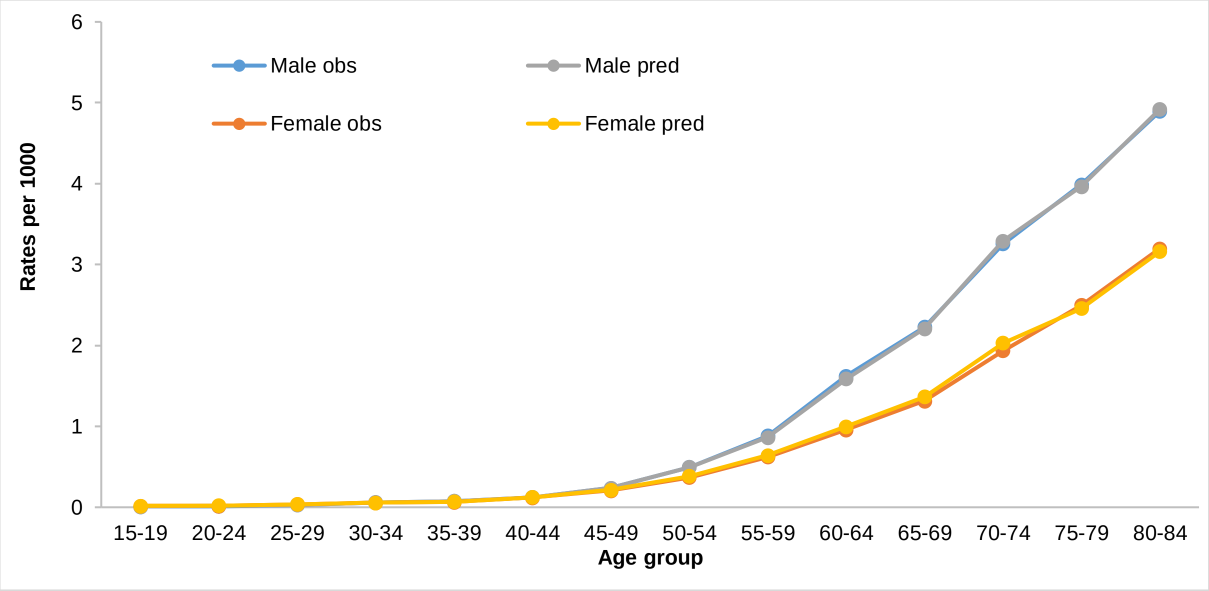

For peer review only - http://bmjopen.bmj.com/site/about/guidelines.xhtml

References

1. Scholes S, Mindell J (2013) *Physical activity in children*. In: Craig R, Mindell J, editors. Health Survey for England 2012.(2013) London: The Health and Social Care Information Centre
2. Wahid A, Manek N, Nichols M *et al.* Quantifying the Association Between Physical Activity and Cardiovascular Disease and Diabetes: A Systematic Review and Meta-Analysis. *J Am Heart Assoc* 2016; 5.
3. Pandey A, Garg S, Khunger M *et al.* Dose-Response Relationship Between Physical Activity and Risk of Heart Failure: A Meta-Analysis. *Circulation* 2015; 132: 1786-1794.
4. Smith AD, Crippa A, Woodcock J *et al.* Physical activity and incident type 2 diabetes mellitus: a systematic review and dose-response meta-analysis of prospective cohort studies. *Diabetologia* 2016.
5. Kyu HH, Bachman VF, Alexander LT *et al.* Physical activity and risk of breast cancer, colon cancer, diabetes, ischemic heart disease, and ischemic stroke events: systematic review and dose-response meta-analysis for the Global Burden of Disease Study 2013. *BMJ* 2016; 354: i3857.
6. Smolina K, Wright FL, Rayner M *et al.* Determinants of the decline in mortality from acute myocardial infarction in England between 2002 and 2010: linked national database study. *BMJ* 2012; 344: d8059.
7. Bamford J, Sandercock P, Dennis M *et al.* A prospective study of acute cerebrovascular disease in the community: the Oxfordshire Community Stroke Project 1981-86. 1. Methodology, demography and incident cases of first-ever stroke. *J Neurol Neurosurg Psychiatry* 1988; 51: 1373-1380.
8. Cowie MR, Wood DA, Coats AJ *et al.* Incidence and aetiology of heart failure; a population-based study. *Eur Heart J* 1999; 20: 421-428.
9. Sharma M, Nazareth I, Petersen I Trends in incidence, prevalence and prescribing in type 2 diabetes mellitus between 2000 and 2013 in primary care: a retrospective cohort study. *BMJ Open* 2016; 6: e010210.
10. Cancer Research UK. Breast cancer statistics. 2016. [accessed 26 October 2016.] Available from: http://www.cancerresearchuk.org/sites/default/files/cstream-node/cases_crude_f_breast_2.xls.
11. Cancer Research UK. Bowel cancer incidence. 2016. [accessed 26 October 2016.] Available from: http://www.cancerresearchuk.org/sites/default/files/cstream-node/cases_crude_bowel_2.xls.
12. Bamford J, Sandercock P, Dennis M *et al.* A prospective study of acute cerebrovascular disease in the community: the Oxfordshire Community Stroke Project--1981-86. 2. Incidence, case fatality rates and overall outcome at one year of cerebral infarction, primary intracerebral and subarachnoid haemorrhage. *J Neurol Neurosurg Psychiatry* 1990; 53: 16-22.

For peer review only - http://bmjopen.bmj.com/site/about/guidelines.xhtml

13. MacIntyre K, Capewell S, Stewart S *et al.* Evidence of improving prognosis in heart failure: trends in case fatality in 66 547 patients hospitalized between 1986 and 1995. *Circulation* 2000; 102: 1126-1131.

14. Turner D, Raftery J, Cooper K *et al.* The CHD challenge: comparing four cost-effectiveness models. *Value Health* 2011; 14: 53-60.

15. Bronnum-Hansen H, Davidsen M, Thorvaldsen P *et al.* Long-term survival and causes of death after stroke. *Stroke* 2001; 32: 2131-2136.

16. Seshasai SR, Kaptoge S, Thompson A *et al.* Diabetes mellitus, fasting glucose, and risk of cause-specific death. *N Engl J Med* 2011; 364: 829-841.

17. Office for National Statistics. Cancer survival in England - adults diagnosed. 2016. [accessed 26 October 2016.] Available from: http://www.ons.gov.uk/file?uri=/peoplepopulationandcommunity/healthandsocialcare/conditionsanddiseases/datasets/cancersurvivalratescancersurvivalinenglandadultsdiagnosed/20102014/finalreferencetablev2.xls.

For peer review only
